# Conflicting attitudes between clinicians and women regarding maternal requested caesarean section: a qualitative evidence synthesis

**DOI:** 10.1186/s12884-023-05471-2

**Published:** 2023-03-28

**Authors:** Margareta Johansson, Jonatan Alvan, Agneta Pettersson, Ingegerd Hildingsson

**Affiliations:** 1 Department of Women’s and Children’s Health, Uppsala University, Akademiska University Hospital, SE- 751 85 Uppsala, Sweden; 2 Swedish Agency for Health Technology Assessment and Assessment of Social Services, Stockholm, Sweden; 3 Department of Women’s and Children’s Health, Uppsala University, Uppsala, Sweden

**Keywords:** Caesarean section, Clinicians, Maternal request, Non-medical, Qualitative evidence synthesis

## Abstract

**Background:**

Caesarean section (CS) can be a life-saving operation but might also negatively affect the health of both the woman and the baby. The aim of this study was to synthesize and contrast women’s and clinicians’ attitudes toward maternal-requested CS, and their experiences of the decision-making process around CS.

**Methods:**

The databases of CINAHL, MEDLINE, PsycInfo and Scopus were screened. All qualitative studies that answered the study question and that were assessed to have minor or moderate methodological limitations were included. Synthesised findings were assessed using GRADE-CERQual.

**Results:**

The Qualitative Evidence Synthesis included 14 qualitative studies (published 2000–2022), involving 242 women and 141 clinicians. From the women’s perspectives, two themes arose: *women regarded CS as the safest mode of birth*; and *women’s rights to receive support and acceptance for a CS request*. From the clinicians’ perspectives, four themes emerged: *clinicians were concerned about health risks associated with CS; demanding experience to consult women with a CS request; conflicting attitudes about women’s rights to choose a CS;* and *the importance of respectful and constructive dialogue about birthing options.*

**Conclusion:**

Women and clinicians often had different perceptions regarding the right of a woman to choose CS, the risks associated with CS, and the kind of support that should be part of the decision-making process. While women expected to receive acceptance for their CS request, clinicians perceived that their role was to support the woman in the decision-making process through consultation and discussion. While clinicians thought it was important to show respect for a woman’s birth preferences, they also felt the need to resist a woman’s request for CS and encourage her to give birth vaginally due to the associated increases in health risks.

**Supplementary Information:**

The online version contains supplementary material available at 10.1186/s12884-023-05471-2.

## Introduction

The prevalence of Caesarean section (CS) births has been steadily increasing over time [1], with the largest rise in middle- and high-income countries [2]. A CS can be a life-saving operation, but might also be a harmful intervention when no medical indication is present; it might negatively affect women’s and children’s health [3, 4]. It might also be regarded as unequal, described by Miller in terms of ‘too much too soon, too little too late’, referring to the overuse of interventions (such as CS) versus delayed or inadequate care [5].

The global incidence of CS performed upon maternal request has been estimated to be 0.2–42% of all births, and the financial status of a country is the dominant factor associated with incidence, as shown in a systematic review and meta-regression of 31 scientific papers from 14 countries covering more than 5 million births [6].

Fear of birth, fear of labour pain, fear that the baby would be hurt or even die, and worries about urine incontinence and perineal damage are common reasons for requesting a CS [7]. A previous negative birth experience, a physician’s suggestion, worries about losing control, worries about prolonged labour or large babies, and anticipated lack of support from clinicians were other reasons reported in a recent systematic review of 28 scientific papers by Jenabi et al. [7]. Some demographic characteristics were also more often seen among women who requested a CS, such as older age, higher body mass index, lower level of education, and not living with a partner [7].

O’ Donovan and O’Donovan [8] compiled 16 qualitative studies into a systematic review on the reasons behind caesarean delivery on maternal request and identified three overall themes, the first being *Birth preferences are influenced by social norms*; these norms come from family values, friends, and media, but also cultural aspects and women’s reproductive rights to choose their mode of birth. The theme *Emotional experiences* involves fear of birth, which includes fearing pain, loss of dignity, and the unpredictable; the theme also involved an assessment that a CS was a safer and less risky alternative that provided control. The third theme involved *Personal experiences*, wherein a negative birth experience was most prominent: this experience could be caused by a previous vaginal birth or a previous emergency CS. The opinions of clinicians also influenced women, whether they were encouraged by medical doctors to choose CS or they had experienced lack of support from clinicians during previous births.

Opinions about CS among clinicians working in maternity care are diverse. A multi-country study was conducted in 2001–2002 in 150 maternity units with 1530 obstetricians, asking the latter about their willingness to perform a CS upon maternal request. Obstetricians from Spain were least likely (15%) and obstetricians from the UK were most likely (79%) to agree to do so [9]. A recent Australian survey showed divergent attitudes between midwives and obstetricians on some indications for CSs but also some agreement on maternal request for a CS, such as the idea that women should be informed and counselled about the risks and benefits (pros and cons) of CS [10].

Another systematic review and meta-synthesis, based on 34 studies (quantitative, qualitative, and mixed-methods) reported obstetricians’ and midwives’ views on the factors that influence the decision to perform a caesarean section by maternal request. The study involved 7785 obstetricians and 1197 midwives from 20 countries, and showed that the personal beliefs of clinicians, the healthcare system, and the clinicians’ characteristics were linked to obstetricians’ and midwives’ views on the factors that influence the decision to perform a CS [11]. Perception of risk and viewing CS as a safe option was one influential factor; fear of litigation, lack of resources (such as physicians not facilitating a normal birth), and private or public insurances and payment systems all contributed to increases in performing CS.

When it comes to mode of birth, a woman’s choice is hotly debated, and it depends on the legal situation (including rights legislation that allows women to make an informed choice about the mode of birth) [12]; the organization of health care, in terms of being publicly or privately run [13]; remuneration systems [14]; convenience [15]; and the management of vaginal birth after a CS [16]. It is obvious that performing CS upon maternal request is a complex issue that involves social norms and legal issues about choice, maternal characteristics, and maternal preferences, as well as clinicians’ characteristics and views. The literature is missing studies comparing women’s and clinicians’ opinions about CS. Therefore, the aim of the Qualitative Evidence Synthesis was to synthesize and contrast women’s and clinicians’ attitudes toward maternal-requested CS, as well as the experiences of the decision-making process around CS.

## Methods

### Study design

**Fig. 1:**
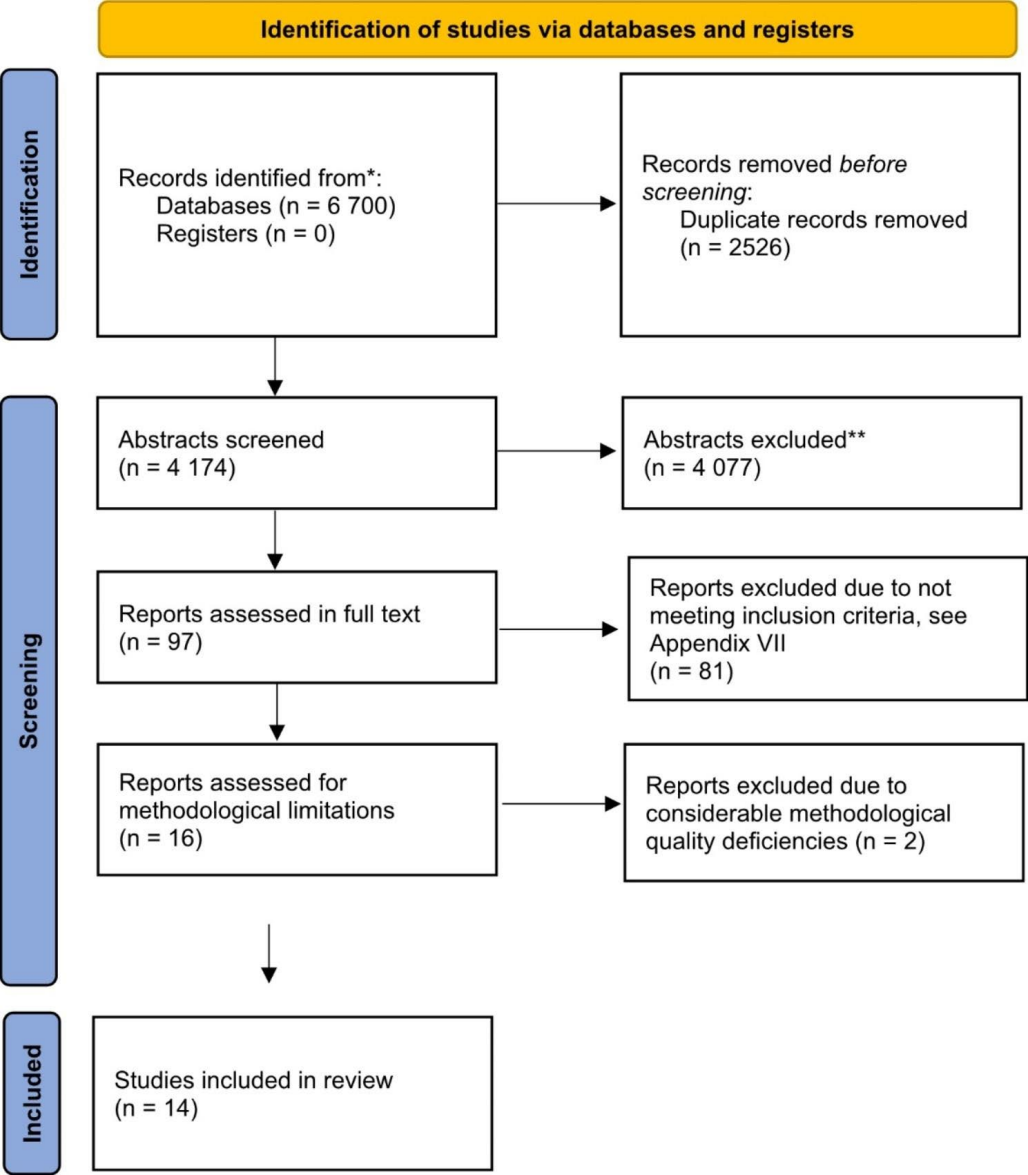
PRISMA 2020 flow diagram for new systematic reviews which included searches of databases and registers only *Number of records identified from each database are specified in Appendix I-IV **No automation tools were used, all records were excluded manually *From*: Page MJ, McKenzie JE, Bossuyt PM, Boutron I, Hoffmann TC, Mulrow CD, et al. The PRISMA 2020 statement: an updated guideline for reporting systematic reviews. BMJ 2021;372:n71. doi: 10.1136/bmj.n71 more information, visit: http://www.prisma-statement.org/

The Qualitative Evidence Synthesis (QES) was part of a full Health Technology Assessment (HTA) report by the Swedish agency for health technology assessment and social service (SBU). The focus of this study was maternally-requested elective CS from two different perspectives: the women’s and the clinicians’ [17]. For transparency, the protocol for the systematic review was registered in the International Prospective Register of Systematic Reviews PROSPERO in 2020, available at https://www.crd.york.ac.uk/prospero/display_record.php?ID=CRD42020207049. The Preferred Reporting Items for Systematic Reviews and Meta-Analyses (PRISMA) guideline [18] (Fig. 1) and the Enhancing Transparency in Reporting the Synthesis of Qualitative Research (ENTREQ) statement were both followed [19].

### Search strategy and selection criteria

The literature search and the selection of studies was based on the *Setting, Perspective, Interest, Comparison and Evaluation* (SPICE) method, which includes structured questions to investigate human experiences and attitudes [20, 21]. For this study, the answers to these questions were: (S): *Where?* All kinds of maternity care; (P): *For whom?* Women who turn to maternity care with a request for a primary or repeat CS, and clinicians who handle the request; (I): *What is under investigation?* Women requested a CS, and clinicians involved in CS discussions with women; (C): *If appropriate, or something else?* N/A; and (E): *What kinds of results?* Attitudes and experiences.

A literature search was conducted by a librarian in the databases of CINAHL, MEDLINE, PsycInfo, and Scopus in November 2022; it combined search blocks of appropriate search terms with regard to the phenomena of interest (childbirth via CS upon the pregnant woman’s request) and the evaluation parameters (experiences and attitudes). The search was limited to studies containing the methodological search terms often used in studies with a qualitative methodology; see Appendix I-IV for the full search strategy. Studies considered to be eligible for this QES were qualitative studies corresponding with the SPICE criteria, published in peer-reviewed journals between 2000 and 2022 (in order to cover as many aspects of the phenomena in interest as possible), and written in English, Swedish, Danish, or Norwegian. Additional search methods included a manual screening of the reference lists of eligible studies and consulting experts for relevant articles in the area.

### Selection of studies

Three of the authors (MJ,IH,AP) independently screened the abstracts and subsequently separately assessed the full texts for relevance. Discrepancies in screening/assessment were resolved through discussion, with all authors if necessary, until consensus was reached.

### Quality assessment

Articles that fulfilled the eligibility criteria were independently assessed for quality by three authors (MJ,IH,JA); discrepancies in assessments were resolved by discussion until consensus was reached. In order to facilitate confidence in the evidence, a checklist focusing on risks of the identified limitations (rather than the limitations per se) was used. The checklist was developed by SBU and comprises five domains: accordance of philosophical theory and study methodology, selection of participants, collection of data, analyses of data and role of the researcher (including relations with participants and handling of pre-understanding). The domains were assessed separately. Finally, a total assessment was made wherein limitations were classified as minor or moderate for the included studies (Appendix V). Two studies were assessed with considerable methodological quality limitations and were excluded from further analyses.

### Synthesis

**Fig. 2:**
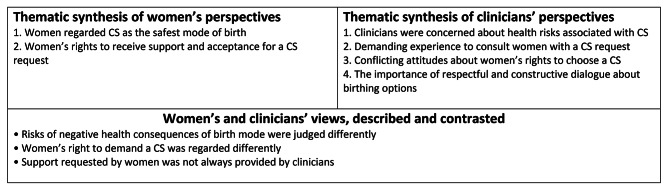
Explored themes from the women’s and clinicians’ perspectives which were contrasted

A Qualitative Evidence Synthesis (QES) was conducted, involving two descriptive thematic syntheses (inspired by the work of Thomas and Harden [22]) from women’s perspectives and clinicians’ perspectives, respectively. The analyses were performed stepwise. First, meaning units and content that corresponded to the aim of our review question were extracted. Then, sentences or sections of study findings with similar content were identified and were grouped into sub-themes. In the third step, the sub-themes were organised into six themes (Appendix VI and VII; Fig. 2). All levels of the thematic synthesis were descriptive, and no theoretical interpretation was performed—i.e., all analyses remained close to the original data. Two researchers (IH,MJ) conducted the extraction and synthesis for women’s perspectives, and two (MJ,IH) did the same for clinician’s perspectives. All four researchers participated in continuously refining the themes, to ascertain no loss of information and appropriate descriptions of themes. In the last step, women’s experiences were described and contrasted with clinicians’ experiences in order to gain a comprehensive understanding. This was an interpretative step, going beyond the original data. Thereafter, one external and two internal reviewers from SBU scrutinized the synthesis step-by-step, and discrepant views of correct coherence and descriptions of themes were resolved through discussion between the authors and the reviewers.

The four researchers contributed through their different backgrounds and experiences. Two of the researchers (MJ,IH) have extensive experience as clinical midwives and as researchers with knowledge of qualitative research methods; the other two researchers (JA,AP) were project managers at SBU, with experience in conducting QESs but without clinical experience in maternity health care. The composition of the team minimized the risk that findings would be biased by pre-understandings.

### Assessment of confidence in the evidence

The confidence in the findings was assessed using GRADE-CERQual [20, 21]. As defined by GRADE-CERQual, four aspects were considered: methodological limitations, relevance, data adequacy, and coherence. Confidence was classified as very low (⊕), low (⊕⊕), moderate (⊕⊕⊕), or high (⊕⊕⊕⊕) [20, 21].

## Results

The literature search for original studies resulted in 4 174 abstracts; of these, the full texts of 97 articles were read. (The manual search did not result in any included studies.) Eighty-one articles were excluded because they did not correspond to the inclusion criteria, and two articles were excluded due to extensive methodological limitations [23, 24] (Appendix VIII). In total, 14 studies were included in the data analyses (Fig. 1), twelve which were assessed as having minor and two as having moderate methodological quality limitations. For descriptions of the authors, publication year, country, aim, setting/recruitment, participants, data collection, and data analyses of the studies, see Table 1. Eight studies focused on women’s perspectives [25–32], two studies focused on clinicians’ perspectives [33, 34] and four studies investigated both women’s and clinicians’ perspectives [35–38].

The two thematic syntheses included 14 qualitative studies, involving 242 women and 141 clinicians from five countries (Table 1). Four studies were conducted in the United Kingdom [25, 33, 37, 38], four in Australia [26, 27, 29, 32], three in Norway [30, 35, 36], two in Sweden [31, 34], and one in Canada [28]. The participants were recruited from hospitals [28–36, 38], from prenatal clinics [33, 34, 37, 38], and through advertisements in newspapers [26, 27].

The studies investigating women’s perspectives had a variety of aims. Eight studies explicitly investigated women’s request for a CS when no medical indication was present [26–31, 35, 36], one study investigated pregnant women who considered a CS [38], one study explored the decision-making process around mode of birth in women with a previous CS [25], and two studies included women with and without medical indications for a CS [32, 37].

Similarly, the studies focusing on clinicians’ perspectives had different aims. Two studies investigated clinicians’ experiences of their encounters with pregnant women who requested a CS in the absence of a medical reason [35, 36], and one study focused on clinicians’ experiences of women who had considered CS as a mode of birth [38]. One study examined factors associated with maternal request for a CS after a previous surgical birth [33], one study investigated attitudes about performing CS upon maternal request [34], and one study addressed midwives’ experiences of non-medical and medical indications for CS [38].

Three studies were based on some type of theory [28, 29, 33] and one drew on *experience-based co-design* [37]; the rest of the studies presented no philosophical approach, theory, or model [25–27, 30, 31, 34–36, 38]. Data in the included studies were analyzed according to the principles of text systematic condensation [35, 36], thematic analysis [25, 27, 32, 38], constant comparison [26, 34], content analysis [31, 34], experience-based co-design [37], grounded theory [28], descriptive phenomenology [29], and interpretative biographical method [30] (Table 1).

**Table 1:** Details for the characteristics of the included studies

Authors/Year/Country	Aim	Setting/Recruitment	Participants	Data collection	Data analyses
Eide et al. (2019) Norway [35]	To provide a qualitative exploration of maternal requests for a planned caesarean section in Norway, in the absence of obstetric indications.	University hospital in Norway. Women recruited consecutively. Referred for birth counselling with a CS request. Purposive sample of midwives.	17 Women 27–42 years of age (14 multipara and 3 primipara). 9 midwives 11 obstetricians.	Women: Semi-structured in-depth interviews. Professionals: focus group discussions.	Systematic Text Condensation.
Eide et al. (2020) Norway [36]	To explore women’s access to patient-centred counselling for concerns initiating caesarean requests in absence of obstetric indications in pregnancy, and to identify tensions, barriers and facilitators affecting such care.	University hospital in Norway. Informants recruited consecutively. Purposive sample.	17 women (1 nullipara and 16 multipara). 9 midwives 11 obstetricians.	Women: Semi-structured in-depth interviews. Caregivers: focus group discussions.	Systematic Text Condensation a method for thematic analysis presented within the frames of Levesque et al.
Emmett et al. (2006) UK [25]	To explore women’s experiences of decision making about mode of delivery after previous caesarean section.	Identified through medical records Maternity units in England and Scotland.	21 women with previous CS experience.	Semi-structured interviews.	Thematic framework.
Fenwick et al. (2006) Australia [26]	To describe the childbirth expectations, influences and knowledge of a group of Western Australian women who experienced a caesarean section (CS) and would prefer a CS in a subsequent pregnancy.	Advertisement in newspapers in one Australian city (Perth).	49 women with previous experience of CS and preferred CS in subsequent pregnancy.	Telephone interviews.	Constant comparison method by the principles of grounded theory.
Fenwick et al. (2010) Australia [27]	To describe Australian women’s request for caesarean section in the absence of medical indicators in their first pregnancy.	Advertisement in regional and local newspapers in the states of QLD and WA, Australia.	14 women requested CS during first pregnancy absence of known medical indication.	Telephone interviews.	Thematic analysis.
Kamal et al. (2005) UK [33]	To explore the views of health professionals on the factors influencing repeat caesarean section.	Two hospitals with maternity care and from midwifery teams.	25 midwives and doctors.	Interviews	Constant comparative method by the principles of grounded theory.
Karlström et al. (2009) Sweden [34]	To describes obstetricians’ and midwives’ attitudes towards CS on maternal request.	Purposive sample of midwives and obstetricians from 3 hospitals and antenatal clinics in Sweden.	16 midwives and 9 obstetricians.	Focus group discussions.	Content analysis.
Kenyon et al. (2016) UK [37]	This article documents a project that was undertaken as collaboration between Birmingham Women’s NHS Foundation Trust, the University of Birmingham and women who had used the BWNFT service.	The experience of both clinical staff and women who had experienced maternal request for CS pathway.	15 women, 10 obstetricians, 12 midwives, 17 health care professionals in a workshop (midwives, midwifery managers, student, research midwives and obstetric consultants).	Semi-structured interviews for women. Professionals in a joint workshop.	Framework by experience-based co-design methodology.
Kornelsen et al. (2010) Canada [28]	To explore women’s experiences of the decision-making process leading to elective operative delivery without medical indication.	Five hospitals in British Columbia. Third party recruitment, identified with (i) chart notation by antepartum and labour ward nurse, (ii) community-based public health postpartum visits nurses, (iii) poster advertisement in 25 obstetrician gynaecologists’ offices, (iv) advertisement in parenting magazine.	17 primiparous women who had undergone a patient-initiated elective Caesarean section in the absence of any medical indication.	Explorative in-depth interviews.	Grounded theory techniques.
McGrath et al. (2009) Australia [29]	To explore, from the mothers’ perspective, the process of decision-making about mode of delivery for a subsequent birth after a previous Caesarean Section.	Obstetric department at a hospital. Women consecutively enrolled from RH hospital list, who had all had a previous CS and a subsequent birth at RH six years prior to the interviews.	16 multiparous women who chose to birth by elective caesarean.	Interviews.	Thematic analysis.
Ramvi et al. (2011) Norway [30]	To investigate specifically women who requested a caesarean section due to fear, but who still gave birth vaginally despite this fear. The fear, the decision-making process, and the vaginal birth experience were explored from the women’s perspective.	A part of an intervention study “Team Midwifery”. Recruited from a hospital.	5 women.	Narrative interviews.	Biographical, narrative, interpretative method.
Sahlin et al. (2013) Sweden [31]	To describe the underlying reasons for the desire for a caesarean section in the absence of medical indication in pregnant first-time mothers.	One Swedish hospital. Recruited at the obstetrician visit after CS decision was taken.	12 first-time mothers.	Individual interviews.	Qualitative content analysis.
Thirukumar et al. (2021) Australia [32]	To understand women’s mode-of-birth preferences and shared decision-making experiences during planned cesarean birth	Eight Australian metropolitan hospitals Women who would undergo planned CB were given the option to indicate their willingness to participate in interviews.	33 women who had undergone a planned CB	Telephone audio-recorded Interviews.	Inductive thematic analysis.
Weaver et al. (2007) UK [38]	To examine whether, and in what context, maternal requests for caesarean section are made.	Participants were recruited from antenatal clinics and hospitals.	44 women who had considered, or been asked to consider, caesarean section during pregnancy were interviewed postnatally. 29 obstetricians.	Interviews.	Thematic analysis.

### Thematic synthesis of women’s perspectives

The thematic synthesis from the women’s perspectives was based on twelve studies [25–32, 35–38], involving 242 study participants. From the women’s perspectives, two themes arose, with six sub-themes. The first theme was *Women regarded CS as the safest mode of birth*, and the second theme was *Women’s rights to receive support and acceptance for a CS request.* The themes are presented with the sub-themes below.

### Theme 1: women regarded CS as the safest mode of birth

Eleven studies with 237 informants contributed to the theme [25–29, 31, 32, 35–38] (moderate confidence in the evidence ⊕⊕⊕), which reveals that women who preferred a CS often regarded vaginal birth as risky and CS as a safe mode of birth associated with little or no risk. Potential risks of CS were ignored or minimized, but after having had a CS, women sometimes re-evaluated their views of the risk. Information about risks and benefits was described as adequate, limited, or contradictory. The women would rather receive acceptance in response their request rather than information. This theme consists of three sub-themes.

The first sub-theme was based on information from six studies [25, 27–29, 32, 38]. It reveals how women viewed CS as a safe mode of birth with minor risks and vaginal birth as a riskier alternative. The women ignored or minimized the probability and seriousness of risks associated with CS. They reported having trust in the competence of the surgical team, which also decreased the perception of risk and relinquished responsibility of the birth to the clinicians. An illustrative quote from one study explained: *Megan articulated that a caesarean section allowed her to have ’a perfect orchestrated birth’ with all the right people in the right place at the right time* [27].

The second sub-theme was based on nine studies [25–29, 31, 35, 36, 38]. It describes how women felt that there are risks with both vaginal birth and CS. Some women had factual knowledge about the risks associated with CS, but they were willing to take the risk: they anticipated a healthy baby and thought that CS was a more predictable and controlled mode of birth. Discussions with medical doctors reinforced their hesitance for vaginal birth. After a previous CS, labour and birth was viewed as unpredictable and insecure. Women could also feel guilty toward the baby or vulnerable due to poor mental health, as reported: *The women’s discourses indicated that birth was about ’getting a baby’ rather than ‘having a baby’… it did not matter how the baby came out and it was unnecessary to feel ‘fulfilled’* [27]. Another study explained by the quote: *Did not want more information of the section because I already knew what to expect, so I knew what was coming if I had another section, and regards to vaginal birth, I didn’t want to know anyway, I didn’t want to ‘go down that path’* [25].

The third sub-theme was based on seven studies [25, 27–29, 32, 36, 37]. Women shared that they had received relevant information about risks, but the information was not given routinely: rather, it had to be requested. The information was also perceived as contradictory or deficient, and information was sometimes collected from non-medical sources. Women with a previous CS would rather receive acceptance for their decision than information. Below are three quotes from the included studies:

When I was asking medical people, what are the risk? It was rather’ oh, you know, there wouldn’t really be any’. I did want a bit more information. [25]

At one stage the hospital was actually very good, they gave me a lot of information to go both ways- And I was sort of reluctant to look at it because I knew the decision I had made. [29]

I did not do any research at all on my own because. . I was afraid of reading fake news about it. [32]

### Theme 2: women’s rights to receive support and acceptance for a CS request

Twelve studies with 242 informants formed the basis of the theme [25–32, 35–38] (moderate confidence in the evidence ⊕⊕⊕), which reveals that women considered it both important and their right to receive support and acceptance for their request for a CS. Many women received support from clinicians for their decision to have a CS, sometimes after negotiation. Sometimes a woman’s request for CS was denied, and sometimes the decision process was regarded as frivolous and lacked support. Women often had to repeat and defend their (in their eyes) well-motivated decisions about the mode of birth. Some women renegotiated their attitude toward mode of birth after previous birth experiences or professional support in the decision-making process. This theme is substantiated by three sub-themes that describe women’s motives and views concerning their request for CS, their experiences of support and treatment, and the strategies they developed when their requests were questioned or denied.

The first sub-theme was based on eight studies [25–28, 31, 35, 36, 38]. Women had the opinion that their motives were well-founded and that they actually had medical risk factors that were indications for CS. They wanted to be in control and feel safe, and they viewed CS as the only option, as they believed that they were unable to give birth vaginally. The women also emphasized their inviolable right to self-determination over their bodies and their mode of birth, although some women raised the problems with complete autonomy. One study explained: *Several women based their request on what they personally considered medical risk factors. They were concerned about complicated births running in their families, previous protracted labor/emergency CS, and perception of having a narrow pelvis or expecting a big baby* [35]. Another study reported that the women had always known that they would not give birth vaginally. One woman said: *… My wish for a caesarean section has been there for a long time. Even before I knew that, I wanted children*. [31]

The second sub-theme was built on ten studies [25, 27–32, 36–38]. Women sometimes perceived unconditional support or encouragement for their birth preferences, but they also faced lack of engagement and time during consultations, feeling questioned, or denial of their CS request. Support from clinicians was important and could lead to women daring to give birth on their own terms. The decision-making process was perceived as inaccurate or unserious when women were forced to undergo counselling, which made them feel condemned, violated, or pressured; this could also occur when the decision about mode of birth was made late in the pregnancy, when documentation of their motives for CS was incomplete, or when it mirrored the clinicians’ own preferences. Two illustrative quotes explain:

She (GP) was actually supportive of a caesarean from the day I found out I was pregnant. Yes, she was like, ‘well I would be having a Caesar if I was you’. [29]

When I tried to explain my situation, I was not heard, I simply had no choice…it was an inhumane decision. [30]

The third sub-theme was based on seven studies [25–28, 30, 35, 37]. The sub-theme explains how if women’s requests or wishes were denied or neglected, they developed strategies to obtain their CS anyways, through increased determination or repeating and defending their requests. Women with a previous CS sometimes became more open, sometimes more ambivalent toward CS in connection with their next pregnancy. One illustrative example: *Denying a request for a caesarean section, would encourage women to find another doctor who would perform the procedure* [27]. One woman explained her experience:

It was fine in itself, she [the consultant midwife] was a perfectly nice lady, I just knew it was something that I had to do to get to the next step in the process of them saying yes to the elective section. So it was kind of like we talked for an hour but to me there was no point, she wasn’t going to say anything that made me change my mind. [37]

### Thematic synthesis of clinicians’ perspectives

The thematic synthesis from the clinicians’ perspectives was based on six studies [33, 34, 36–38], involving 141 study participants. From the clinicians’ perspectives, four themes emerged, with ten sub-themes. The themes were: *Clinicians were concerned about health risks associated with CS, Demanding experience to consult women with a CS request, Conflicting attitudes about women’s rights to choose a CS*, and *The importance of respectful and constructive dialogue about birthing options.* The themes are presented with their sub-themes below.

### Theme 1: clinicians were concerned about health risks associated **with CS**

Three studies with 70 informants contributed to the theme [33, 34, 36] (moderate confidence in the evidence ⊕⊕⊕), in which is evident that clinicians were concerned about the higher health risks associated with CS delivery compared to giving birth vaginally, and they were worried about the increasing prevalence of CS. In the decision-making process, they felt it was important to include considerations of health risks/consequences and to share these with the women. This theme involved two sub-themes.

The first sub-theme was explained by three studies [33, 34, 36]. It notes that the clinicians had concerns about the increased prevalence of non-medical CS, because it was regarded as involving greater risk of complications compared to a vaginal birth [33, 34], and the increased number of CS reduced the capacity for treating other obstetrical and gynecological conditions [36]. Clinicians’ experiences were reported as: *Some obstetricians saw it as (…) especially in low-risk pregnancies where the evidence suggest a VD* (vaginal delivery) *was undeniably the safest option for mother and child.* [36]; and *The doctors were also concerned about the medical consequences of a high CS rate in terms of more complicated births in the future.* [34]

The second sub-theme was based on two studies [34, 36]. Clinicians were of the opinion that the consequences and risks of CS must be considered and must be communicated with women, without violating their integrity, during the decision-making process [34, 36]. Clinicians’ experiences can been seen in the following description: *Participants also discussed the responsibility of both caregivers and expecting parents to consider the medical implications on an increasing CS rate* [34].

### Theme 2: demanding experience to consult women with a CS request

Six studies with 141 informants contributed to the theme [33–38] (moderate confidence in the evidence ⊕⊕⊕), which describes how women’s requests for CS were regarded by clinicians as based on misunderstandings about the advantages and disadvantages of a CS. Clinicians perceived it to be a demanding and time-consuming experience to handle a woman’s CS request. Clinicians felt that a high workload often led to complications during childbirth or negative birth experiences, that—when combined with deficient postnatal care—often led women to request delivery by CS in their next pregnancy. This theme consists of three sub-themes.

The first sub-theme was based on five studies [34–38]. Clinicians described that it was demanding to handle a woman’s request for a CS, and they could be resistant to or reluctant about the request. They believed it was a balancing act to direct women’s expectations with regard to the professionals’ and society’s expectations and the preference of a vaginal birth [34–38]. Clinicians regarded the consultations in which women requested CS as time-consuming and mentally exhausting [36]. *Consultant midwives* experienced their role as involving conflict when they had to persuade women to undergo a vaginal birth, instead of supporting their mode of birth preference [36]. The clinicians shared: *… did not see it worth their time and resources when it came to a patient dispute, which was regarded, time-consuming and mentally exhausting* [36].

The second sub-theme was based on four studies [34–37]. Clinicians found it to be difficult to inform women about the health risks of CS without frightening them [34, 35]. Clinicians perceived women’s information as inconsistent [37] and not always based on scientific evidence [34, 36, 37]. Women’s requests for CS were believed to be based on misunderstandings about advantages and disadvantages of a CS [34–37]. Below is one illustrative example: *Midwives acknowledged the difficult balance of providing enough information without creating unnecessary fear* [35].

The third sub-theme was based on five studies [33–35, 37, 38]. Clinicians concluded that a heavy workload made it difficult to create a supportive and safe environment for women in labour [33, 34, 37], with the consequences of complicated childbirths [34, 38], negative birth experiences [34], insufficient time for postnatal debriefing with women [33], and thus maternal requests for CS in subsequent pregnancies [33, 34]. Below is one example describing one clinician’s experience:

Such stress in the intrapartum care was perceived to complicate births, leave the woman with a negative birth experience and be the cause of maternal request in a subsequent pregnancy [34].

### Theme 3: conflicting attitudes about women’s rights to choose a CS

Five studies with 121 informants contributed to the theme [33, 34, 36–38] (moderate confidence in the evidence ⊕⊕⊕), which reveals that clinicians hold conflicting attitudes about women’s right to choose CS for their mode of birth. The clinicians had different beliefs about the definition of a medically indicated CS, including whether childbirth fear was a medical or a non-medical indication for CS. Clinicians felt it was important to have scientific evidence for the decision-making process. Health care organization and the capacity of the health care system were believed to influence a CS decision. This theme encompasses three sub-themes.

The first sub-theme was based on three studies [33, 34, 36]. It reveals that clinicians hold conflicting attitudes about women’s right to choose CS for their mode of birth [33, 34, 36]. The clinicians felt that a woman has the right to choose CS if she is well informed [33, 36] or that a woman has no right to choose CS [33]. Some illustrative findings from the studies:

Furthermore, the obstetricians noted that they …. accept(ed) women’s request for CS rather than dealing with a ‘worst case scenario’ if they promoted a vaginal birth [34].

Some obstetricians saw it as their main responsibility to inform the patient and help the patient make an informed choice about mode of delivery. If she were able to make an informed choice, her choice should be respected. [36]

The second sub-theme was based on four studies [33, 34, 36, 38]. Clinicians felt it was important to have standardized definitions for medical and non-medical CS [33], but these are absent [33]. Some clinicians regarded childbirth fear as an indication for CS, while others did not [38]. Clinicians regarded it as important that the decision about mode of birth was made based on the individual woman’s unique circumstances rather than on a strict protocol [33]. One example of a clinician’s experience is given here: *Although a role for guidelines and protocols was recognised, caesarean section was identified in participants’ accounts as an area that was especially ill suited to being managed by strict protocols that would apply in each and every situation* [33].

The third sub-theme was based on three studies [33, 36, 37]. Scientific evidence was described as an influential factor in the decision of mode of birth, but not the only factor in a CS decision. Clinicians argued whether available scientific evidence was of high methodological quality or not; sometimes, the evidence was difficult to assess and judge. Other influential factors in a CS decision were described as professional boundaries, content of care, structure of maternity care organization, and financial aspects [33, 36, 37]. One clinician shared: *However, most accounts also emphasised that ‘evidence’ was not the sole basis for decision making* [33]; and another one: *The health budget for delivery clinics is performance based and paradoxically pays more for a CS than a VD* (vaginal delivery) [36].

### Theme 4: the importance of respectful and constructive dialogue about birthing options

Six studies with 141 informants contributed to the theme [33–38] (moderate confidence in the evidence ⊕⊕⊕), which describes how the clinicians believed that support in the decision-making process involves engaging in respectful and constructive dialogue about birthing options. They considered it important to give women different kinds of support when consulting with them about a CS request. Support was preferably provided through evidence-based information, consultation in early pregnancy, and showing respect and understanding for the women’s request. Including a discussion about CS alternatives in the consultation was regarded as significant by the clinicians. Supporting women was regarded as enhancing women’s ability give birth vaginally, reducing women’s CS requests, and lower the prevalence of CS. This theme encompasses two sub-themes.

The first sub-theme is based on six studies [33–38]. It reveals that the clinicians found it important to support women before and during their pregnancy as well as postnatally [33, 34, 36–38]. It was important to offer women different kinds of support [34], such as evidence-based information [34] and consultations with women early in pregnancy. Professional support enhanced clinicians’ ability to give women the confidence to give birth vaginally [34]. Clinicians’ experiences can be seen in the following description: *Participants believed that information at an early stage and to a sufficient degree could change women’s preferences towards vaginal birth* [34].

The second sub-theme was based on three studies [34, 36, 38]. It was important to provide women with enough consulting time [38], respect, and understanding [34, 36, 38]. Promoting a constructive dialogue [36] and including discussion of a vaginal birth alternative were also important in the consulting process [34, 36, 38]. Professional support enhanced clinicians’ ability to strengthen women’s confidence in giving birth vaginally [34], decreased women’s CS requests [34], and lowered the prevalence of CS [34]. Clinicians’ experiences can be seen in the following findings reported:

Showing respect and taking women seriously often helped them re-establish trust, which had commonly been lost in an earlier birth experience. [36]

Many of the doctors stressed the importance of taking time with women to find out what was behind the request, and where appropriate, exploring safe and acceptable alternatives. [38]

### Women’s and clinicians’ views, described and contrasted

#### Risks of negative health consequences of birth mode were judged differently

Women who requested CS without medical indication regarded it to be the safest mode of birth when the risks and benefits were considered. Potential risks of CS were ignored or minimized, but after having had a CS, they sometimes re-evaluated their view of the risk. Information about risks and benefits were described as adequate, limited, or contradictory. In contrast, the clinicians explained that CS entails higher health risks compared to vaginal birth, and they expressed worries over the increasing CS prevalence. In the decision-making process, it was seen as important to include the considerations of health risks consequences. Women’s requests for CS were considered by the clinicians to be based on misunderstandings about the advantages and disadvantages of CS. Health care organizations were described as having an effect on CS decisions.

#### Women’s right to demand a CS was regarded differently

The women considered themselves as having a right to demand a CS and have their request for mode of birth be accepted, and they often had to repeat and defend their (in their eyes) well-motivated decisions about mode of birth. The clinicians held widely varying and conflicting views regarding the extent to which a woman has a right to choose the mode of birth herself. The clinicians had different opinions about the definition of a medically indicated CS and about whether childbirth fear was a medical or a non-medical indication for CS. It was regarded as important to have scientific evidence for the decision-making process around mode of birth.

#### Support requested by women was not always provided by clinicians

The decision-making process for mode of birth was sometimes experienced by women as inattentive. They felt it was important to get support for their birthing preference, but that was not always provided. For some women, support for their request was received after negotiation. Some women re-considered their attitude toward mode of birth as a result of previous birth experiences or professional support in the decision-making process. From the clinicians’ perspective, women’s CS requests were perceived as a demanding and time-consuming task. Clinicians felt it was important to provide different kinds of support when consulting women with a CS request. Support was preferably provided through evidence-based information, consultation in early pregnancy, and showing respect and understanding for a women’s request. Clinicians felt it was important to include a discussion about CS alternatives in the consultation. Supporting women was regarded as enhancing women’s ability give birth vaginally, reducing women’s CS requests, and lowering the prevalence of CS. The clinicians thought that the high workload in the labour wards might complicate the birthing process, resulting in negative birth experiences, limiting the possibility of follow-up after birth, and thus leading to requests for CS in subsequent pregnancies.

### Assessment of confidence according to GRADE-CERQual

Confidence in the evidence regarding the thematic syntheses from the women’s perspectives [25–32, 35–38] and the clinicians’ perspectives [33, 34, 36–38] was assessed at theme level. We assessed the confidence in the evidence as moderate for all six themes, according to GRADE-CERQual [20, 21]. The rationale for the assessments were as follows.

Regarding methodological limitations, two of the 14 included studies were assessed to have moderate methodological limitations, and twelve were assessed to have minor methodological limitations. This could be a limitation in confidence in the evidence for the themes that contained data from the two studies with moderate limitations [20, 21].

In the matter of relevance, several of the included studies were conducted in countries that have rights legislation and private actors in the maternity care system, which may influence the general view of CS. This might be a limitation in confidence in the evidence regarding transferability to a broader birth context [20, 21].

Concerning data adequacy, the data were regarded as rich, with many details. In addition, the quantity of data (number of studies, participants, and meaning units) was considered adequate. However, some of the included studies had a different, broader, or more narrow purpose than the aim of our review, which constitutes a risk that some aspects of the phenomena were not brought up during interviews which might decrease the confidence in the evidence [20, 21].

There were no serious limitations in coherence. The synthesis was descriptive keeping close to the original data. Findings were validated and re-validated at all steps in the process by three separate reviewers.

From our experiences, the findings show great resemblance to the clinical situation in the context of interest. The confidence in the evidence is further strengthened by the fact that the attitudes and experiences of both women and clinicians were rather similar, regardless of which country the study was conducted in [20, 21]. Therefore, our overall conclusion was that each of the possible limitations do not seem to have seriously affected the findings. We assessed that they together led to a lower confidence rating in confidence by one level. See Appendix V for details about the assessments of confidence.

## Discussion

The main findings of this study are based on two qualitative thematic syntheses that were contrasted; they revealed that women and clinicians often had different perceptions regarding the right of a woman to choose CS, the risks associated with CS, and the kind of support that should be part of the decision-making process. While women expected to receive acceptance and support in their decision to undergo a CS, clinicians perceived that their role was to support the woman in the decision-making process through consultation and discussion. While clinicians thought it was important to show respect for a woman’s mode of birth preference, they also felt the need to resist a woman’s request for CS and encourage her to give birth vaginally, due to the associated increases in health risks with CS.

Women felt that they received support for their request when it was accepted. Clinicians viewed support as encouraging vaginal birth, having time for consultative discussions, and showing respect, but also being resistant to a mode of birth that might increase health risks. Clinicians also felt that a high workload would increase birth complications, which might lead to negative birth experiences; that, combined with lack of time to follow up with women’s postnatal experiences might result in requests for CS in subsequent pregnancies. The results also showed a disparity in clinicians’ approaches in the decision-making process, primarily based on their own attitudes and opinions, which were likely to be transferred to the women. It is unknown whether the discrepancy is based on lack of communication or limited understanding.

### Women believed they had an unquestionable right to choose a CS but were directed toward a vaginal birth

*The right to choose a Caesarean section* was seen in the results of the syntheses as being important to women but doubted by clinicians. This might be a manifestation of the power imbalance between women and clinicians. Such an imbalance might result in a feeling of not being taken seriously and not being worth considering [39]. Although women in Sweden do not have any legal right to choose CS, there are probably few women who are denied a CS, especially if they present with a fear of birth [40]. The women in the synthesis regarded an approved CS request as the ultimate support. From the clinicians’ perspective, the aspect of making a well-founded decision about mode of birth and the importance of their professional right to refuse an operation on a healthy woman were considered important. A systematic review comprising 55 scientific papers on clinicians’ views about women’s right to choose mode of birth [41] showed that obstetricians were more willing than midwives to comply with such requests, as were clinicians in countries with private health care systems, male clinicians, and clinicians who themselves preferred CS [41]. Women with a previous CS might be a more vulnerable group. Historically, the expression ‘once a Caesarean, always a Caesarean’ was the golden standard; however, in present day, women are expected to give birth vaginally if the indication for a CS in the former pregnancy is not present in the current pregnancy. A Cochrane review of Vaginal Birth After Caesarean (VBAC), based on three scientific studies with 2270 women, concluded that 65% of the women who preferred a VBAC gave birth vaginally and 97% of those who preferred a CS had a CS [16], which might be seen as unequal.

Another discrepancy was found in *what constitutes a medical indication*. In several studies, women considered themselves to have well-founded motives and medical risk factors that required a CS. Some examples were given, such as it being common with complicated births in the family, a previous protracted labour, a previous emergency CS, a narrow pelvis, an expected large baby, or having diabetes [29, 35]. There was also disagreement among clinicians as to what counts as an absolute indication for a CS: some argued that fear of birth was an indication for a CS, while others argued that it was not [33, 34, 36, 38].

### Health risks considerations in the decision-making process around CS

The result showed that women and clinicians discussed the risks of different modes of birth, sometimes from diametrically opposed positions. Women viewed vaginal birth as being associated with greater risks compared to CS, while clinicians emphasized greater risks with CS. Lack of communication and information was a problem found in both syntheses. It was difficult for clinicians to find a balance between affirming women’s autonomy and taking on the possible risks that an operation can entail. This struggle for balance was found in several of the included articles and was described as ‘meeting women’s request[s] for a CS with resistance *and* respect’. Previous studies from Norway have shown that when midwives in counselling services challenge women’s CS requests while simultaneously providing women with tools to manage their childbirth fears, women tended to withdraw their request [42, 43]. It is also important to bear in mind the problems built into discussing risk. Evidence is based on the total state of knowledge, with data presented at the group level; thus, communicating risks to an individual woman can involve trade-offs, due to the fact that she is totally affected or unaffected by a risk or a complication.

### Women regarded the decision-making process as inadequate, despite good intentions of clinicians

The decision-making process was described by the women as being long and obscure, with late referral and decisions about the mode of birth. This situation increased psychological stress during pregnancy. The intention of the clinicians was to establish a relationship with the woman and to have a dialogue about the decision. Many women felt that the clinicians were supportive during the process. It is well known that attitudes of clinicians affect women’s choices and desires [9]. The results of this synthesis showed that clinicians sometimes tried to reverse women’s positions, to preferring a vaginal birth. This view was grounded in evidence about vaginal birth being less risky while CS is more risky—especially when no medical indication is present.

In the present study, the focus was not on the reasons behind preferences for CS, but previous studies have shown that fear of birth and/or a previous negative birth experience are the most common reasons for a CS request, as shown in several systematic reviews [7, 8, 44]. No study thus far has been able to prove that fear of birth is cured by an operation.

### The question of autonomy

A model of autonomy might explain the complexity in the case of CS where no medical indication is present and the discrepancy between women’s and clinicians’ attitudes and experiences [44]. The model was developed through a concept analysis in order to understand women’s choices regarding place of birth, and it can be transferred to the choice regarding mode of birth. In light of the results found in these thematic syntheses, the problem about performing CS upon maternal request could be embedded between the concepts ‘Do no harm’ and ‘Take responsibility for the outcome’, discussed in terms of *Information, Capacity*, and *Freedom* [45].

*Information* and knowledge about risks are important to consider before making any decision about mode of birth. If one specific mode of birth would increase the risks for the baby, some people might argue that the woman’s autonomy should be restricted [46]. Pregnant women should be offered information about available choices, together with the risks and benefits of different modes of birth, but the results from the thematic syntheses show that women do not always value the information, or that the information was neglected or minimized. Both syntheses showed that non-evidence-based information could affect women in a variety of ways and that information needs to be provided at an early stage. A previous meta-ethnographic study of 20 scientific papers about women’s experiences of VBAC showed that women who sought a VBAC were likely to incorporate available evidence and thereby manage the risks associated with VBAC [47]. In the present thematic syntheses, this question was often discussed in a reverse situation, when women thought that they had medical risk factors that were not confirmed by the clinicians. It was also noticed that the clinicians became frustrated when they met demanding women. Clinicians’ attitudes certainly affect their patients and might result in the women feeling less empowered [47]. Evidence-based decision aid has been suggested as a way to help women maintain their autonomy [48]; despite the limited effect of using shared decision aids, it might limit conflicts in the decision-making process and familiarize women with the terminology used in health care settings [49].

*Capacity* refers to a woman’s ability to understand and process information. It is obvious that holding the role of ‘patient’ might result in subordination, but it is important to remember that pregnant women are usually not ill and are rather able to make their own informed decisions. However, situations may occur where requests are founded on misunderstandings or misleading information, or primarily based on fear or anxiety [50]. It was also evident from the thematic syntheses that sometimes women gathered information from non-medical sources; whether this information is evidence-based or not is unclear.

*Freedom of choice* is an essential and delicate component of autonomy that might be internal or external. Laws, regulations, and traditions might restrict external freedom, while internal freedom could be restricted by, e.g., anxiety [45]. There are some ethical aspects connected to freedom of choice. On the one hand, it is important to respect women’s autonomy; on the other hand, it is also important to consider the health aspects and risks for both the woman and the unborn baby. The baby’s right to the best mode of birth must be also considered, which might contradict some of the results in the synthesis wherein women argued for their right to their bodies. This autonomy could potentially increase risks to the baby. When considering the baby’s rights, one might agree or disagree with an editorial in *The Lancet* [51] discussing place of birth: the author claimed that “women have the right to choose where to give birth, but have no right to expose the baby to any risk?” The discussion about personal and professional autonomy will hopefully continue, but ethical aspects, paternalism, and the consequences of women’s and clinicians’ recommendations must also be incorporated.

### Strengths and limitations

The two thematic syntheses addressed women’s and clinicians’ perspectives on mode of birth, with a focus on maternal request for CS. The primary studies varied and comprised both experiences of CS as a mode of birth, perceptions about CS, and factors associated with the decision-making process, as well as CS as a mode of birth after a previous CS. The findings seem to be robust, as the syntheses included steps to ensure confirmability and credibility. The scientific base was 14 studies, with an acceptable number of participants and rich material. A potential limitation is that the included studies did not have the same focus for research as us; thus, there might have been some perspectives that were not brought up in the data collection.

The strength of the results might face some restrictions when it comes to transferability. Five of the 14 original studies were performed in Sweden and Norway, with a tax-financed health care system in which private care providers are rare; in the other nine studies included in the thematic syntheses, private care is more common (UK, Australia, and Canada). In Sweden, women have no legal right to demand a CS: the decision is made by an obstetrician, often in collaboration with the woman. In other countries, women are allowed to independently choose the mode of birth themselves. Moreover, in Sweden, women are usually recommended a vaginal birth after a previous CS, unless certain risk factors are identified; in other countries, it is more common to recommend a repeat CS. The above-listed factors might result in different views in comparisons of Sweden with other countries. It is also possible that the other reasons for performing a CS differ.

The limitations of transferability and relevance were considered in the evaluation process. Despite the differences between countries, the experiences were quite similar regardless of where the studies were conducted, which strengthens the reliability of the results. The included studies had only low or moderate methodological limitations, which also contributes to the good reliability of the results. Transparency was enhanced by the protocol’s registration in PROSPERO and by the fact that PRISMA [18] and ENTREQ [19] guided the report of our syntheses [19].

## Conclusion

Women and clinicians often had different perceptions regarding the right of a woman to choose CS, the risks associated with CS, and the kind of support that should be part of the decision-making process. While women expected to receive acceptance for their CS request, clinicians perceived that their role was to support the woman in the decision-making process through consultation and discussion. While clinicians thought it was important to show respect for a woman’s birth preferences, they also felt the need to resist a woman’s request for CS and encourage her to give birth vaginally due to the associated increases in health risks. Recommendation for further research is to develop and test a decision-making protocol for healthcare professionals to use when pregnant women ask for planned caesarean section as mode of birth where no medical reason is presented.

## Electronic supplementary material

Below is the link to the electronic supplementary material.

**Appendix I:** CINAHL via EBSCO 12 November 2022

**Appendix II:** MEDLINE via OvidSP 22 November 2022

**Appendix III:** PsycInfo via EBSCO 22 November 2022

**Appendix IV:** Scopus via Elsevier 22 November 2022

**Appendix V.** The quality assessments for each included paper

**Appendix VI.** Data analysis process for women’s perspectives

**Appendix VII.** Data analysis process for clinicians’ perspectives

**Appendix VIII.** Excluded studies

**Appendix IX.** PRISMA 2020 Checklist

## Data Availability

The data supporting the conclusions of this article is available in the published articles: Eide et al. 2019 [35], Eide et al. 2020 [36], Emmett et al. 2006 [25], Fenwick et al. 2006 [26], Fenwick et al. 2010 [27], Kamal et al. 2005 [33], Karlström et al. 2009 [34], Kenyon et al. 2016 [37], Kornelsen et al. 2010 [28], McGrath et al. 2009 [29], Ramvi et al. 2011 [30], Sahlin et al. 2013 [31], Thirukumar et al. 2021 [32], Weaver et al. 2007 [38].

## Abbreviations

CS: Caesarean section
ENTREQ: Enhancing Transparency in Reporting the Synthesis of Qualitative Research
HTA: Health Technology Assessment
QES: Qualitative Evidence Synthesis
SBU: Swedish agency for health technology assessment and social service
SPICE: Setting, Perspective, Interest, Comparison and Evaluation
VBAC: Vaginal Birth After Caesarean
