# Supplementary material for: Conflicting attitudes between clinicians and women regarding maternal requested caesarean section: a qualitative evidence synthesis
Source: BMC Pregnancy Childbirth. 2023 Mar 28;23:210. doi: 10.1186/s12884-023-05471-2 (PMC10044365; doi:10.1186/s12884-023-05471-2)
Supplement: Supplementary file 1 — Appendix I: CINAHL via EBSCO 12 November 2022 [file 12884_2023_5471_MOESM1_ESM.docx]

CINAHL via EBSCO 12 November 2022

| Search terms | | Items found |
| --- | --- | --- |
| Childbirth with Caesarean section on the pregnant woman's request | | |
|  | ((MH "Cesarean Section, Elective" OR MH "Cesarean Section, Repeat" OR MH "Cesarean Section+/EI/EV/NU/PF/TD/UT") OR TI("abdominal deliver*" OR "c-section*" OR cesarean OR caesarean OR cesarian OR caesarian OR cesarien OR caesarien OR "non-labour" OR "non-labor") OR AB(("abdominal deliver*" OR "c-section*" OR cesarean OR caesarean OR cesarian OR caesarian OR cesarien OR caesarien OR "non-labour" OR "non-labor") N15 ("no clinical" OR "non clinical" OR "no medical" OR "non-medical" OR "non-urgent" OR "on demand" OR overuse OR "over use" OR option* OR request* OR unnecessary OR "without medical indication")) | 13,938 |
|  | TI(choice* OR decision* OR demand* OR preference* OR request* OR wish OR wishes OR "no clinical" OR "non clinical" OR "no medical" OR "non-medical" OR "non-urgent" OR "on demand" OR overus* OR "over use" OR request* OR unnecessary OR "without medical" OR (absence W3 medical)) OR (AB((maternal OR mother* OR women*) N3 (choice* OR decision* OR demand* OR preference* OR request* OR wish OR wishes)) OR ("no clinical" OR "non clinical" OR "no medical" OR "non-medical" OR "non-urgent" OR "on demand" OR overus* OR "over use" OR request* OR unnecessary OR "without medical" OR (absence W3 medical))) | 203,066 |
|  | *1 AND 2* | *1,456* |
|  | ((MH "Cesarean Section+") AND (MH "Decision Making" OR MH "Decision Making, Patient" OR MH "Decision Making, Shared" OR MH "Decision Making, Ethical" OR MH "Maternal Attitudes" OR MH "Patient Autonomy" OR MH "Patient Attitudes" OR MH "Patient Preference" OR MH "Unnecessary Procedures")) | 1,292 |
|  | *3 OR 4* | *2,284* |
| Causes delivery choice | |  |
|  | ((MH "Childbirth+/EI/NU/PF" OR MH "Delivery, Obstetric/EI/NU/PF" OR MH "Pregnancy/PF") OR TI(((birth OR delivery) N3 (mode OR preference*)) OR (child N3 birth*) OR childbirth* OR parturition*)) AND ((MH "Anxiety" OR MH "Fear" OR MH "Frustration" OR MH "Maternal Attitudes"  OR MH "Patient Autonomy" OR MH "Rape/EI/NU/PF" OR MH "Stress Disorders, Post-Traumatic/PF/NU/EI/EH" OR MH "Unnecessary Procedures") OR TI(anxiet* OR fear* OR FOC OR pain* OR tokophobia OR counselling OR counseling)) | 1,750 |
| Encounters, experiences | | |
|  | MH "Anxiety+" OR MH "Attitude to Pregnancy" OR MH "Caregiver Support" OR MH "Decision Making" OR MH "Decision Making, Patient" OR MH "Decision Making, Shared" OR MH "Decision Making, Ethical" OR MH "Emotions+" OR MH "Fear" OR MH "Maternal Attitudes"  OR MH "Nurse-Patient Relations" OR MH "Physician-Patient Relations" OR MH "Professional-Patient Relations" OR MH "Obstetrics" OR MH "Patient Assessment" OR MH "Patient Attitudes" OR MH "Patient Preference" OR MH "Patient Satisfaction" OR MH "Professional-Client Relations+" OR MH "Professional-Family Relations" OR MH "Psychological Well-Being" OR MH "Social Norms" OR MH "Support, Psychosocial" OR MH "Unnecessary Procedures" | 569,546 |
|  | TI((anxiet* OR attitude* OR distress* OR encounter* OR expectation* OR experience* OR fear* OR interaction* OR judgement* OR perception* OR posttraumatic* OR "post traumatic" OR "psychological trauma*" OR rape OR relation* OR resistance* OR respect* OR trust OR wellbeing OR "well being" OR worry OR worries) AND (birth* OR childbirth* OR delivery OR labor OR pregnan* OR women)) OR TI((maternal OR mother* OR patient* OR pregnant*) AND (caregiver* OR clinical OR midwi?e* OR nurs* OR obstetric* OR personnel OR physician* OR profession* OR provider*)) | 126,851 |
|  | AB((anxiet* OR attitude* OR belief* OR comfort* OR concern* OR encounter* OR expectation* OR experience* OR fear* OR interaction* OR judgement* OR perception* OR posttraumatic* OR "post traumatic" OR "psychological trauma*" OR rape OR relation* OR relief* OR resistance* OR respect*) OR wellbeing OR "well being" OR worry OR worries) N15 (birth* OR childbirth* OR delivery OR labor OR pregnan* OR women)) OR AB((belief* OR empath* OR encounter* OR expectation* OR experience* OR interaction* OR judgement* OR perception* OR relation* OR resistance* OR respect*) N15 (caregiver* OR clinical OR midwi?e* OR nurs* OR obstetric* OR personnel OR physician* OR profession* OR provider*)) | *375,951* |
|  | *7 OR 8 OR 9* | *938,234* |
| Study types: Qualitative Research | |  |
|  | MH "Qualitative Studies" OR MH "Ethnographic Research" OR MH "Ethnological Research" OR MH "Ethnonursing Research" OR MH "Grounded Theory" OR MH "Phenomenological Research" OR MH "Field Studies" OR MH "Focus Groups" OR MH "Interviews" OR MH "Semi-Structured Interview" OR MH "Structured Interview" OR MH "Unstructured Interview" OR MH "Narratives" OR MH "Discourse Analysis" OR MH "Thematic Analysis" OR MH "Content Analysis" OR MH "Multimethod Studies" OR MH "Purposive Sample" OR MH "Quota Sample" OR MH "Snowball Sample" OR MH "Theoretical Sample" OR MH "Convenience Sample" OR MH "Phenomenology" OR TI "constant comparison" OR TI "constant comparative" OR TI ethnon* OR TI "human science" OR TI "discourse analy*" OR TI "focus group*" OR TI "grounded research" OR TI "grounded studies" OR TI "grounded study" OR TI "grounded theor*" OR TI hermeneutic* OR TI interview* OR TI "life experiences" OR TI "lived experience*" OR TI "meta-ethno*" OR TI "mixed method*" OR TI "narrative analy*" OR TI "purposive sampl*" OR TI phenomenol* OR TI qualitative OR TI questionnaire* OR AB "constant comparison" OR AB "constant comparative" OR AB ethnon* OR AB "human science" OR AB "discourse analy*" OR AB "focus group*" OR AB "grounded research" OR AB "grounded studies" OR AB "grounded study" OR AB "grounded theor*" OR AB hermeneutic* OR AB interview* OR AB "life experiences" OR AB "lived experience*" OR AB "meta-ethno*" OR AB"mixed method*" OR AB "narrative analy*" OR AB "purposive sampl*" OR AB phenomenol* OR AB qualitative OR AB questionnaire* | 785,030 |
|  | 10 OR 11 | *1,437,846* |
| **Combined sets** | | |
|  | 5 AND 12 | *1,721* |
|  | 6 AND 12 | *1,598* |
| **Final sets** | | |
|  | **13 OR 14** **Limiters - Peer Reviewed; Publication Type: Journal Article; Language: Danish, English, Norwegian, Swedish** | **1,641** |
|  | **13 OR 14**  **Limiters - Publication Type: Meta Synthesis, Review, Systematic Review; Language: Danish, English, Norwegian, Swedish** | **199** |

The search result, usually found at the end of the documentation, forms the list of abstracts.

AB = Abstract

AU = Author

DE = Term from the thesaurus

MM = Major Concept

TI = Title

TX = All Text; performs a keyword search of all the database's searchable fields

ZC = Methodology Index

* = Truncation

“ “ = Citation Marks; searches for an exact phrase
