# Supplementary material for: Conflicting attitudes between clinicians and women regarding maternal requested caesarean section: a qualitative evidence synthesis
Source: BMC Pregnancy Childbirth. 2023 Mar 28;23:210. doi: 10.1186/s12884-023-05471-2 (PMC10044365; doi:10.1186/s12884-023-05471-2)
Supplement: Supplementary file 2 — Appendix II: MEDLINE via OvidSP 22 November 2022 [file 12884_2023_5471_MOESM2_ESM.docx]

MEDLINE via OvidSP 22 November 2022

| Search terms | | Items found |
| --- | --- | --- |
| Childbirth with Caesarean section on the pregnant woman's request | | |
|  | (exp Cesarean Section/es, nu, px or Cesarean Section, Repeat/es, nu, px, td or Vaginal Birth after Cesarean/es, nu, px, td) or (abdominal deliver* or c-section* or cesarean or caesarean or cesarian or caesarian or cesarien or caesarien or elective or "non-labour" or "non- labor").ti,kf. | 47912 |
|  | ("no clinical" or "non clinical" or "no medical" OR "non-medical" or "on demand" or overus* or request* or unnecessary or "without medical" or (absence adj3 medical)).ti,kf. or ((choice* or decision* or demand* or preference* or request* or wish or wishes) adj3 (birth* or childbirth* or deliver* or parturition*)).ti,kf. | 19105 |
|  | ((maternal or mother* or women*) adj3 (decision* or demand* or preference* or request* or wish or wishes)).ab. or ((choice* or decision* or preference*) adj3 (birth* or childbirth* or deliver* or parturition*)).ab. or (("no clinical" or "non clinical" or "no medical" or "non-medical" or "non-urgent" or "on demand" or overus* or request* or unnecessary or "without medical" or (absence adj3 medical)) and (abdominal deliver* or c-section* or cesarean or caesarean or cesarian or caesarian or cesarien or caesarien or "non-labour" or "non-labor")).ab. | 14375 |
|  | limit 3 to "pubmed not medline" | 1468 |
|  | *2 OR 4* | *20507* |
|  | *1 AND 5* | *872* |
|  | ((Cesarean Section/ or Cesarean Section, Repeat/ or Vaginal Birth after Cesarean/)  AND (Choice Behavior/or exp decision making/ or Decision Making, Shared/ or maternal behavior/ or patient preference/ or Professional-Patient Relations/ or exp health services misuse/)) | 1468 |
|  | *6 OR 7* | *2078* |
|  | (exp Delivery, Obstetric/es, nu, px or Parturition/es, px or Pregnancy/px or (birth* or childbirth* or parturition*).ti,kf.) and ((Anxiety/px or Fear/es, px or frustration/ or psychological distress/ or Psychological Trauma/eh, nu, px or Stress Disorders, Post-Traumatic/nu, px) or (anxiet* or distress* or fear* or FOC or pain* or tokophobia or counselling or counseling).ti,kf) | 3312 |
|  | *8 OR 9* | 5298 |
| Encounters, experiences | | |
|  | anxiety/ or "Attitude of Health Personnel"/ or attitude to health/ or bereavement/ or Choice Behavior/ or exp decision making/ or Decision Making, Shared/ or emotions/ or fear/ or frustration/ or exp health services misuse/ or health knowledge, attitudes, practice/ or maternal behavior/ or Patient Acceptance of Health Care/ or patient satisfaction/ or patient preference/ or Professional-Patient Relations/ or professional-family relations/ or nurse-patient relations/ or physician-patient relations/ or prejudice/ or psychosocial deprivation/ or psychological distress/ or sadness/ or social norms/ or social values/ or therapeutic alliance/ or "Quality of Life"/px or trust/ | 1031682 |
|  | (anxiet* or attitude* or comfort* or concern* or distress or encounter* or expectation* or experience* or fear* or FOC or interaction* or judgement* or perception* or posttraumatic* or "post traumatic" or psycholog* or rape or relation* or relief* or tokophobia or trust or wellbeing OR "well being" or worry or worries).ti,kf. | 1646640 |
|  | ((anxiet* or attitude* or comfort* or concern* or distress or encounter* or expectation* or experience* or fear* or FOC or interaction* or judgement* or perception* or posttraumatic* or "post traumatic" or psycholog* or rape or relation* or relief* or tokophobia or trust or wellbeing OR "well being" or worry or worries) AND (maternal or mother* or patient* or pregnant* or women*)).ab. | 1915460 |
|  | limit 13 to "pubmed not medline" | 44828 |
|  | *11 OR 12 OR 14* | *2439183* |
| Study types: Qualitative research | | |
|  | (Focus Groups/ or Interviews as Topic/ or interview/ or Interview, Psychological/ or grounded theory/ or Nursing Methodology Research/ or phenomenology/ or qualitative research/ or Questionnaires/) or (content analys* or cluster sampl* or constant comparative method* or discourse analys* or ethnographic research or ethnological research or ethnonursing research or field stud* or field work* or grounded theory or hermeneutic* or interview* or lived experience* or mixed method* or narrativ* or phenomenological or purposive sample or qualitative or thematic analys* or theoretical sampl* or questionnaire*).ab,ti,kf. | 1566989 |
|  | *15 OR 16* | *3503614* |
| Study types: meta-syntesis/review | |  |
|  | (exp qualitative research/ and "review"/) or ("mixed methods".ti,ab. and "review"/) or ((review or systematic or meta or synthes*) adj3 (qualitative or narrative OR interpret* or integrative OR evidence)).af. or (meta adj3 (study OR studies OR synthes* OR ethnograph* OR "data analys*" OR summary)).af. or ((realist OR framework OR thematic OR "mixed method*") adj3 (synthes* OR review)).af. or ((meta* OR review OR synthes* OR systematic) adj3 "grounded theory").af. or ((information OR data) adj2 synthes*).af. or (metasynthes* OR metaethnograph* OR "qualitative cross-case analysis" OR "aggregated analys*" OR "critical interpretive synthesis" or "meta-narrative").af. | 166897 |
| Combined sets | | |
|  | *10 AND 18* | *106* |
|  | *10 AND 17* | *4326* |
| Final sets | | |
|  | **limit 19 to ((danish or english or norwegian or swedish) and journal article)** | **105** |
|  | **limit 20 to ((danish or english or norwegian or swedish) and journal article)** | **3,846** |

The search result, usually found at the end of the documentation, forms the list of abstracts.

.ab. =Abstract

.ab,ti. = Abstract or title

.kf.= Author keywords

Exp= Term from the Medline controlled vocabulary, including terms found below this term in the MeSH hierarchy

.sh.= Term from the Medline controlled vocabulary

.ti. = Title

/ = Term from the Medline controlled vocabulary, but does not include terms found below this term in the MeSH hierarchy

* = Focus (if preceding a MeSH term)

* or $= Truncation (if found at the end of a free text term)

.mp=text, heading word, subject area node, title
