# Supplementary material for: Conflicting attitudes between clinicians and women regarding maternal requested caesarean section: a qualitative evidence synthesis
Source: BMC Pregnancy Childbirth. 2023 Mar 28;23:210. doi: 10.1186/s12884-023-05471-2 (PMC10044365; doi:10.1186/s12884-023-05471-2)
Supplement: Supplementary file 3 — Appendix III: PsycInfo via EBSCO 22 November 2022 [file 12884_2023_5471_MOESM3_ESM.docx]

PsycInfo via EBSCO 22 November 2022

| Search term | | Items found |
| --- | --- | --- |
| Childbirth with Caesarean section on the pregnant woman's request | |  |
|  | DE "Caesarean Birth" OR TI("abdominal deliver*" OR "c-section*" OR cesarean or caesarean or cesarian or caesarian or cesarien or caesarien OR "non-labour") OR AB ("abdominal deliver*" OR "c-section*" OR cesarean or caesarean or cesarian or caesarian or cesarien or caesarien OR "non-labour")) | 2,322 |
|  | TI(("no clinical" OR "non clinical" OR "no medical" OR "non-medical" OR "non-urgent" OR "non-urgent" OR "on demand" OR overuse OR "over use" OR request* OR unnecessary OR "without medical" OR (absence W3 medical)) OR ((maternal OR mother* OR wom?n*) AND (decision* OR demand* OR preference* OR request* OR wish OR wishes))) OR AB("no clinical" OR "non clinical" OR "no medical" OR "non-medical" OR "non-urgent" OR "non-urgent" OR "on demand" OR overuse OR "over use" OR request* OR unnecessary OR "without medical" OR (absence W3 medical)) | 82,419 |
|  | *1 AND 2* | *235* |
| Causes delivery choice | |  |
|  | (DE "Birth" OR TI(birth* OR childbirth* OR deliver* OR parturition*)) AND TI(choice* OR counselling OR counseling OR decision* OR mode OR preference* OR request* OR tokophobia) | 1,042 |
|  | *3 OR 4* | *1,195* |
| Encounters Experiences | |  |
|  | DE "Anxiety" OR DE "Birth Trauma" OR DE "Distress" OR DE "Emotional States" OR DE "Anxiety Sensitivity" OR DE "Fear" OR DE "Health Anxiety" OR DE "Health Personnel Attitudes" OR DE "Neurosis" OR DE "Panic Attack" OR DE "Counselor Attitudes" OR DE "Post-Traumatic Stress" OR DE "Psychologist Attitudes" OR DE "Rape" OR DE "Social Interaction" OR DE "Stress Reactions" OR DE "Trauma" | 278,584 |
|  | TI(anxiet* OR attitude* OR encounter* OR expectation* OR experience* OR fear* OR interact* OR judgement* OR perception* OR posttraumatic* OR "post traumatic" OR "psychological trauma*" OR rape OR preference* OR relation* OR resistance* OR wellbeing OR "well being" OR worry OR worries) OR AB(anxiet* OR attitude* OR encounter* OR expectation* OR experience* OR fear* OR interact* OR judgement* OR perception* OR posttraumatic* OR "post traumatic" OR "psychological trauma*" OR rape OR preference* OR relation* OR resistance* OR tokophobia OR wellbeing OR "well being" OR worry OR worries) | 2,497,261 |
|  | *6 OR 7* | *2,549,608* |
| Study types | | |
|  | (DE "Qualitative Methods" OR DE "Focus Group" OR DE "Grounded Theory" OR DE "Interpretative Phenomenological Analysis" OR DE "Narrative Analysis" OR DE "Semi-Structured Interview" OR DE "Thematic Analysis" OR DE "Interviews" OR DE "Mixed Methods Research" OR DE "Observation Methods" OR DE "Phenomenology" OR DE "Qualitative Measures") OR (TX "constant comparative" OR TX ethnon* OR TX "human science" OR TX "discourse analy*" OR TX "focus group*" OR TX "grounded research" OR TX "grounded studies" OR TX "grounded study" OR TX "grounded theor*" OR TX hermeneutic* OR TX interview* OR TX "life experiences" OR TX "lived experience*" OR TX "meta-synthesis" or TX "meta-ethno*" OR "mixed method*" OR TX "narrative analy*" OR TX "purposive sampl*" OR TX phenomenol* OR TX qualitative OR TX questionnaire*) | 1,208,738 |
|  | *8 OR 9* | *2,957,498* |
| Combined sets | | |
| **Final sets** | | |
|  | ***5 AND 10***  ***Limiters - Peer Reviewed; Language: Danish, English, Norwegian, Swedish; Document Type: Journal Article*** | ***688*** |
|  | ***5 AND 10 AND Limiters - Peer Reviewed; Language: Danish, English, Norwegian, Swedish; Methodology: LITERATURE REVIEW, -Systematic Review, METASYNTHESIS*** | ***23*** |

The search result, usually found at the end of the documentation, forms the list of abstracts.

AB = Abstract

AU = Author

DE = Term from the thesaurus

MM = Major Concept

TI = Title

TX = All Text. Performs a keyword search of all the database's searchable fields

ZC = Methodology Index

* = Truncation

“ “ = Citation Marks; searches for an exact phrase
