# Supplementary material for: Conflicting attitudes between clinicians and women regarding maternal requested caesarean section: a qualitative evidence synthesis
Source: BMC Pregnancy Childbirth. 2023 Mar 28;23:210. doi: 10.1186/s12884-023-05471-2 (PMC10044365; doi:10.1186/s12884-023-05471-2)
Supplement: Supplementary file 4 — Appendix IV: Scopus via Elsevier 22 November 2022 [file 12884_2023_5471_MOESM4_ESM.docx]

Scopus via Elsevier 22 November 2022

| Search terms | | Items found |
| --- | --- | --- |
| Childbirth with Caesarean section on the pregnant woman's request | | |
|  | TITLE((abdominal PRE/2 delivey) OR "c-section" OR cesarean OR caesarean OR cesarian OR caesarian OR cesarien OR caesarien OR "non-labour" OR "non-labor") | 30,139 |
|  | TITLE((maternal OR mother* OR women*) W/3 (choice* OR decision* OR demand* OR preference* OR request* OR wish OR wishes)) | 4,232 |
|  | TITLE("no clinical" OR "non clinical" OR "no medical" OR "non-medical" OR "non-urgent" OR "on demand" OR overus* OR "over use" OR request* OR unnecessary OR "without medical" OR (absence PRE/3 medical)) | 35,397 |
|  | 2 OR 3 | 39,281 |
|  | 1 AND 4 | 513 |
|  | TITLE((birth* or childbirth* or deliver* or parturition) W/3 (choice* or decision* or preference* OR tokophobia or counseling OR counselling)) | 1,416 |
|  | 5 OR 6 | 1,894 |
| Encounters, experiences | | |
|  | TITLE-ABS-KEY(anxiet* OR attitude* OR distress* OR encounter* OR expectation* OR experience* OR fear* OR frustration* OR interaction* OR judgement* OR perception* OR posttraumatic* OR "post traumatic" OR preferenc* OR psycholog* OR rape OR relation*) | 15,444,975 |
|  | TITLE-ABS-KEY (resistance* OR respect* OR tokophobia OR trust OR wellbeing OR "well being" OR worry OR worries) | 8,474,592 |
|  | *8 OR 9* | *21,925,888* |
|  | TITLE-ABS-KEY ((content PRE/0 analys*) OR (discourse PRE/0 analys*) OR (comparative PRE/0 method*) OR "discourse analysis" OR "field work” OR (focus PRE/0 group*) OR "grounded theory" OR hermeneutic* OR interview* OR "lived experience" OR (mixed PRE/0 method*) OR narrative*) | 1,218,993 |
|  | TITLE-ABS-KEY (phenomenolog* OR qualitat* OR (thematic PRE/0 analys*) OR questionnaire*) | 2,198,496 |
|  | *11 OR 12* | *3,001,116* |
|  | *10 OR 13* | *23,165,221* |
| Combined sets | | |
|  | 7 AND 14 | 796 |
| **Final set** | | |
|  | **15 AND ( LIMIT-TO ( PUBYEAR ,  2000-2022 )   AND  ( LIMIT TO ( SUBJAREA ,  "MEDI"  OR "NURS" )  OR  "SOCI" )  OR  "PSYC" )  OR    "MULT"  OR "HEAL"   AND  ( LIMITTO ( DOCTYPE ,  "ar" ) )  AND  ( LIMITTO ( LANGUAGE ,  "English"  OR  "Danish"  OR "Norwegian" AND  ( LIMIT-TO ( SRCTYPE ,  "j" ) )** | **525** |

The search result, usually found at the end of the documentation, forms the list of abstracts.

**TITLE-ABS-KEY**  = Title or abstract or keywords
**ALL =** All fields
**PRE/n =** "precedes by". The first term in the search must precede the second by a specified number of terms (n).

**W/n =** "within". The terms in the search must be within a specified number of terms (n) in any order.
***** = Truncation

**“ “** = Citation Marks; searches for an exact phrase

LIMIT-TO ( SRCTYPE ,  "j"  = Limit to source type journal
LIMIT-TO ( DOCTYPE ,  "ar"  = Limit to document type article
LIMIT-TO ( DOCTYPE ,  "re"   = Limit to document type review
