# Supplementary material for: Conflicting attitudes between clinicians and women regarding maternal requested caesarean section: a qualitative evidence synthesis
Source: BMC Pregnancy Childbirth. 2023 Mar 28;23:210. doi: 10.1186/s12884-023-05471-2 (PMC10044365; doi:10.1186/s12884-023-05471-2)
Supplement: Supplementary file 5 — Appendix V. The quality assessments for each included paper [file 12884_2023_5471_MOESM5_ESM.docx]

## **Appendix V.** The quality assessments for each included paper.

The quality assessments for each included paper according to the Swedish agency for health technology assessment and assessment of social service recommendations for qualitative studies (<https://www.sbu.se/globalassets/ebm/bedomning_studier_kvalitativ_metodik.pdf>).

| **Domain** | Accordance of philosophical theory | Participants | Data collection | Data analyses | Role of the researcher | Equipoise of the methodological quality limitations |
| --- | --- | --- | --- | --- | --- | --- |
| *Question* | *Are study aim and question in accordance of philosophical theory?* | *Are there any considerable deficiencies?* | *Are there any considerable deficiencies?* | *Are there any considerable deficiencies?* | *Are there any considerable deficiencies?* |  |
| Eide et al. 2019 [35] | Yes | No | No | No | No | Minor quality limitations |
| Eide et al. 2020 [36] | Yes | No | No | No | No | Minor quality limitations |
| Emmett et al. 2006 [25] | Yes | No | No | No | No | Minor quality limitations |
| Fenwick et al.2006 [26] | Yes | No | No | No | No | Minor quality limitations |
| Fenwick et al. 2010 [27] | Yes | No | No | No | No | Minor quality limitations |
| Kamal et al. 2005 [33] | Yes | No | Unclear | Unclear | Unclear | Moderate quality limitations |
| Karlström et al. 2009 [34] | Yes | Unclear | No | No | Unclear | Moderate quality limitations |
| Kenyon et al. 2016 [37] | Ja | No | No | No | No | Minor quality limitations |
| Kornelsen et al. 2010 [28] | Ja | No | No | No | No | Minor quality limitations |
| McGrath et al. 2009 [29] | Ja | No | No | Unclear | No | Minor quality limitations |
| Ramvi et al. 2011 [30] | Ja | No | No | No | No | Minor quality limitations |
| Sahlin et al. 2013 [31] | Ja | Unclear | No | No | No | Minor quality limitations |
| Thirukumar et al. 2021 [32] | Ja | No | No | No | No | Minor quality limitations |
| Weaver et al. 2007 [38] | Ja | No | No | No | No | Minor quality limitations |
