# Supplementary material for: Conflicting attitudes between clinicians and women regarding maternal requested caesarean section: a qualitative evidence synthesis
Source: BMC Pregnancy Childbirth. 2023 Mar 28;23:210. doi: 10.1186/s12884-023-05471-2 (PMC10044365; doi:10.1186/s12884-023-05471-2)
Supplement: Supplementary file 6 — Appendix VI. Data analysis process for women’s perspectives [file 12884_2023_5471_MOESM6_ESM.docx]

**Appendix VI.** Data analysis process for women’s perspectives.

| **Theme 1:**  **Women regarded CS as the safest mode of birth** | | |
| --- | --- | --- |
| ***Women who preferred a CS often regarded vaginal birth as risky and CS as a safe mode of birth associated with little or no risk. Potential risks of CS were ignored or minimized, but after having had a CS, women sometimes re-evaluated their views of the risk. Information about risks and benefits was described as adequate, limited, or contradictory. The women would rather receive acceptance in response their request rather than information.*** | | |
| **Sub-theme 1: Women viewed CS as a safe mode of birth with minor risks and vaginal birth as a riskier alternative. The women ignored or minimized the probability and seriousness of risks associated with CS. They reported having trust in the competence of the surgical team, which also decreased the perception of risk and relinquished responsibility of the birth to the clinicians [25,27-29,32,38].** | | |
| **Author**  **Year**  **[Reference]** | **Meaning unit** | **Swedish translation** |
| Emmet et al.  2006  [25] | Some women felt that potential health risks were not an important influence on decision making because they were seen as very small. | Några omföderskor med tidigare erfarenhet av kejsarsnitt ansåg att potentiella risker inte hade så stor betydelse för beslutet eftersom de var ovanliga. |
| Fenwick et al.  2010  [27] | Women reported that they minimised the severity of risk. | Förstföderskor med önskemål om kejsarsnitt utan medicinsk indikation minimerade allvarligheten i risker med kejsarsnitt. |
| Fenwick et al.  2010  [27] | Women reported that they minimized the likelihood of risk. | Förstföderskor med önskemål om kejsarsnitt utan medicinsk indikation minimerade sannolikheten av risk vid kejsarsnitt. |
| Fenwick et al.  2010  [27] | Women generally described 'switching off to the risks by filtering. | Förstföderskor med önskemål om kejsarsnitt utan medicinsk indikation stängde av och filtrerade risker. |
| Fenwick et al.  2010  [27] | Others admitted to not wanting to know. | Förstföderskor med önskemål om kejsarsnitt utan medicinsk indikation medgav att de inte ville veta om risker. |
| Fenwick et al.  2010  [27] | Where risks were acknowledged, women often absolved themselves and reassigned responsibility for them to their doctor. | Då deltagarna erkände att det fanns risker (med KS) frikände de sig från dessa och omfördelade ansvaret för riskerna till sin läkare. |
| Fenwick et al.  2010  [27] | Women explicitly commented that medical skill and experience in a controlled environment protected them and made the chance of an adverse incident remote. | Deltagarna uttryckte explicit att medicinsk kunskap och erfarenhet i en kontrollerad miljö skyddade dem och gjorde så att risken för en komplikation var liten. |
| Fenwick et al.  2010  [27] | Most women expressed a sense of trust and faith in their doctor as reflected in statements such as Deanne's; 'I trusted them. I handed control of myself over to them. I was completely in their hands'. | De flesta deltagarna uttryckte att de hade fullt förtroende för sin läkare och att de överlät sig själva att stå helt under läkarens kontroll. |
| Fenwick et al.  2010  [27] | She articulated how she had 'faith and trust in them enough to let them control things'. | Hon uttryckte att hon litade på dem tillräckligt för att låta dem kontrollera saker och ting. |
| Kornelsen et al.  2010  [28] | Many participants perceived that the risks to their baby of a vaginal birth were greater than those attributed to Caesarean section. | Många förstföderskor uppfattade att vaginal födsel var förknippat med större risk än de förknippade med kejsarsnitt. |
| McGrath et al.  2009  [29] | Further reducing the mothers’ sense of risk is confidence in the skill of the operating team. | Omföderskor med tidigare erfarenhet av kejsarsnitt hade tillit till operationspersonalens skicklighet vilket minskade känslan av risk. |
| McGrath et al.  2009  [29] | They (the Operating team) were seen as experienced and unlikely to make mistakes. | Omföderskor med tidigare erfarenhet av kejsarsnitt ansåg att operationspersonalen var erfaren och att det är osannolikt att de gör misstag. |
| McGrath et al.  2009  [29] | The perception of the minimal risk associated with an EC was further reinforced by the knowledge that staff will be available and focused on the birth. | De minimala riskerna med kejsarsnitt förstärktes av vetskapen att personalen är fokuserad på förlossningen enligt omföderskor med tidigare erfarenhet av kejsarsnitt. |
| McGrath et al.  2009  [29] | Caesarean section was also considered safer by this group of EC mothers. | Omföderskor med tidigare erfarenhet av kejsarsnitt ansåg kejsarsnitt vara säkrare än vaginal förlossning. |
| McGrath et al.  2009  [29] | And another indicated she did not even consider the risks. | En omföderska med tidigare erfarenhet av kejsarsnitt övervägde inte risker i förhållande till olika förlossningssätt. |
| McGrath et al.  2009  [29] | The majority of mothers who chose EC strongly believed that VBAC carried the highest risk. | Majoriteten av omföderskorna med tidigare erfarenhet av kejsarsnitt hade en stark åsikt om att vaginal förlossning är mer riskfylld än kejsarsnitt. |
| Thirukumar et al. 2021 [32] | Even though many women would have preferred a VB, they were willing to have a CB if this was recommended by the clinician as safest for the baby or mother: | Många kvinnor skulle ha föredragit vaginal förlossning men de accepterade planerat kejsarsnitt om klinikerna rekommenderade det som det säkraste förlossningssättet för barn och mamma. |
| Thirukumar et al. 2021 [32] | Every birth is good, as long as your baby is here and safe and healthy. (Amirah, requested repeat CB) | Omföderska med önskat planerat kejsarsnitt sa att alla förlossningssätt är bra, så länge sitt barn är tryggt och att barnet är friskt. |
| Thirukumar et al. 2021 [32] | Ideally I would have to give birth naturally, but you know [CSJ is safest thing for me and the baby ... so that's the way I would go. I wouldn't want to put me or the baby at risk. (Carmen, repeat CB due to past negative experience) | En omföderska med tidigare negativ förlossningsupplevelse ansåg naturlig förlossning skulle ha varit idealt, men ansåg för henne och barnet att planerat kejsarsnitt var säkrast. Hon ville inte sätta sig eller sitt barn i en riskfylld situation. |
| Weaver et al.  2007  [38] | Cesarean section was widely perceived as safe. | Kvinnor ansåg i allmänhet att kejsarsnitt var ett säkert förlossningssätt. |
| Weaver et al.  2007  [38] | Cesarean section was widely perceived as safer than vaginal birth for babies. | Kvinnor ansåg kejsarsnitt vara ett säkrare förlossningssätt för barnet jämfört med vaginal födsel. |
| Weaver et al.  2007  [38] | Any risks associated with cesarean section were usually minimized. | Riskerna vid kejsarsnitt ansågs oftast av kvinnorna vara så små att de oftast kunde förbises. |
| **Sub-theme 2:**  **Women felt that there are risks with both vaginal birth and CS. Some women had factual knowledge about the risks associated with CS, but they were willing to take the risk: they anticipated a healthy baby and thought that CS was a more predictable and controlled mode of birth. Discussions with medical doctors reinforced their hesitance for vaginal birth. After a previous CS, labor and birth was viewed as unpredictable and insecure. Women could also feel guilty toward the baby or vulnerable due to poor mental health [25-29,31,35,36,38].** | | |
| **Author**  **Year**  **[Reference]** | **Meaning unit** | **Swedish translation** |
| Eide et al.  2019  [35] | Many women anticipated the mental benefit after a planned CS was worth the longer recovery time in physical terms. | Flera kvinnor med önskemål om kejsarsnitt förväntade sig att den mentala fördelen med ett planerat kejsarsnitt var värt den längre fysiska återhämtningstiden. |
| Eide et al.  2019  [35] | Overall, many women in this category regarded a planned CS would facilitate a mentally stable puerperal period. | Kvinnor med önskemål om kejsarsnitt utan medicinsk indikation uppfattade att ett planerat kejsarsnitt skulle underlätta eftervårdstiden mentalt. |
| Eide et al.  2019  [35] | Overall, many women in this category regarded a planned CS as a predictable. | Flera av kvinnorna med önskemål om kejsarsnitt utan medicinsk indikation ansåg ett planerat kejsarsnitt vara förutsägbart. |
| Eide et al.  2019  [35] | Overall, many women in this category regarded a planned CS as a and calm birth experience. | Kvinnor med önskemål om kejsarsnitt utan medicinsk indikation uppfattade ett planerat kejsarsnitt som en lugn förlossningsupplevelse. |
| Eide et al.  2020  [36] | Women were aware that a VD was preferable for the child, and a planned CS was not regarded as an easy way out | Kvinnor med medvetna om att vaginal födsel var att föredra för barnet, och att ett planerat kejsarsnitt inte sågs som en enkel utväg |
| Eide, et al.  2020  [36] | Many women indicated that they would prefer a VD had it not been for the circumstances underlying their request. Several felt a bad conscience towards the child for not being able to manage a VD. | Många kvinnor sa att de skulle föredra en vaginal födsel om det inte varit för de omständigheter som låg till grund för deras krav. Många fick dåligt samvete gentemot barnet när de inte kunde klara av en vaginal födsel. |
| Eide et al.  2020  [36] | A few women had felt ashamed and vulnerable when having to engage with the health system for a mental health reason. | Några få kvinnor hade känt skam och känt sig sårbara när de varit tvungna att engagera hälso-och sjukvården på grund av mental hälsa. |
| Emmet et al.  2006  [25] | Some women felt that potential health risks were not an important influence on decision making because they were equivalent for the different delivery options. | Kvinnor med tidigare erfarenhet av kejsarsnitt ansåg att potentiella risker inte hade så stor betydelse för beslutet eftersom det var lika vanligt oavsett förlossningssätt. |
| Fenwick et al.  2006  [26] | These women now considered birth to be unpredictable and uncertain. | Kvinnor med tidigare kejsarsnitt uttryckte att förlossning är oförutsägbart och osäkert. |
| Fenwick et al.  2006  [26] | For these women (previous cs) the concept of birth, especially vaginal birth, was now associated with pain. | Kvinnor med tidigare kejsarsnitt ansåg nu att vaginal födsel var associerat med smärta. |
| Fenwick et al.  2006  [26] | For these women (previous cs) the concept of birth associated with the risk of complications. | Kvinnor med tidigare kejsarsnitt ansåg nu att barnafödande var associerat med komplikationer. |
| Fenwick et al.  2006  [26] | Birth is now scary (after a previous CS). | Kvinnor med tidigare kejsarsnitt ansåg nu att förlossning kunde vara skrämmande. |
| Fenwick et al.  2006  [26] | Many women described how they now perceived having a healthy baby to be the *most* important reasonable expectation for childbirth. | Flera kvinnor med tidigare kejsarsnitt rapporterade nu att ett friskt barn var nu den viktigaste förväntningen kring förlossningen. |
| Fenwick et al.  2006  [26] | Many women described how they now perceived having a healthy baby ‘only’ reasonable expectation for childbirth. | Flera kvinnor med tidigare kejsarsnitt rapporterade nu att ett friskt barn var nu den enda förväntningen kring förlossningen. |
| Fenwick et al.  2006  [26] | The final change relates to a move from process to outcome-related expectations. | Kvinnor med tidigare kejsarsnitt rapporterade nu en förändring i sina förväntningar, från att ha sett födelsen som en process till att mer fokusera på utfallet av förlossningen. |
| Fenwick et al.  2010  [27] | Although all women knew that bleeding, infection, and epidural complications were immediate risks to themselves. | Alla kvinnor med önskemål om kejsarsnitt utan medicinsk indikation var medvetna om att det fanns vissa risker med kejsarsnitt såsom blödning, infektioner och komplikationer till ryggbedövning. |
| Fenwick et al.  2010  [27] | Women reported that they were prepared to take their chances (of risks). | Kvinnor rapporterade att de var förberedda på att ta chansen att risk skulle uppstå. |
| Fenwick et al.  2010  [27] | Participants reported that conversations with their doctors about their choice for a non-medically indicated caesarean section reinforced their doubts and fears about labour and vaginal birth. | Kvinnor med önskemål om kejsarsnitt utan medicinsk indikation upplevde att diskussioner med läkare förstärkte deras tvekan och rädslor inför vaginal födsel. |
| Kornelsen et al.  2010  [28] | (…) led to the desire to ensure what they felt would be the best possible outcome, and to reduce risks for the baby. | Många förstföderskor ville försäkra sig om det bästa möjliga utfallet och att minska riskerna för barnet [genom att få ett kejsarsnitt]. |
| McGrath et al.  2009  [29] | The predictability of booking in for the EC and not being open to the unpredictability of a natural delivery. | Omföderskor med tidigare erfarenhet av kejsarsnitt uppfattade kejsarsnitt som förutsägbart och ville inte genomgå en oförutsägbar naturlig (vaginal) förlossning. |
| McGrath et al.  2009  [29] | One of the concerns that mothers confront in making birthing choices after a prior caesarean is the issue of risk associated with either mode of delivery. | Kvinnor med tidigare erfarenhet av kejsarsnitt var bekymrade över risker oavsett förlossningssätt. |
| McGrath et al.  2009  [29] | The key concern for the mothers was expressed to be the risk of rupture of the scar from previous CS. | Kvinnor med tidigare erfarenhet av kejsarsnitt upplevde framförallt en oro för risk för uterusruptur i samband med en vaginal förlossning efter ett tidigare kejsarsnitt. |
| McGrath et al  2009  [29] | Only one mother who chose an EC viewed caesarean as riskier than a VBAC. | Endast en kvinna med tidigare erfarenhet av kejsarsnitt ansåg kejsarsnitt vara mer riskfyllt än vaginal förlossning efter ett kejsarsnitt. |
| McGrath et al.  2009  [29] | However, one mother who chose an EC noted that there are just as many risks with both options. | En kvinna som valde ett planerat kejsarsnitt ansåg riskerna likvärdiga för kejsarsnitt och vaginal förlossning. |
| McGrath et al  2009  [29] | Some mothers reported that the health professionals presented the risks associated with both birthing choices without favouring either choice. | Omföderskor med tidigare erfarenhet av kejsarsnitt upplevde att personalen beskrev risker med båda förlossningssätten utan att förorda något av dessa. |
| Sahlin et al.  2013  [31] | For the interviewed women a caesarean section seemed to be a more controlled and safe way of having a baby compared to vaginal delivery, especially for the fetus. | För deltagarna verkade kejsarsnitt vara ett mer kontrollerat och säkert alternativ jämfört med vaginal förlossning, speciellt för barnet. |
| Sahlin et al.  2013  [31] | Many of the women reported that they feared that a vaginal birth would result in hypoxia for the fetus. | Många av förstföderskorna berättade att de var rädda för att vaginal förlossning skulle resultera i syrebrist hos barnet. |
| Sahlin et al.  2013  [31] | In many interviews the women said that they felt like the situation in general is more controlled during a planned caesarean than a vaginal delivery. | I många intervjuer ansåg kvinnorna att de kände att situationen generellt är mer kontrollerad under ett planerat KS jämfört med en VF. |
| Sahlin et al.  2013  [31] | Several said that a caesarean section means a given date for birth, there is no risk of being referred from 'your hospital'. | Flera av kvinnorna sa att ett kejsarsnitt innebär ett givet datum för förlossning, att det inte finns någon risk att bli remitterad från "ditt" sjukhus. |
| Sahlin et al.  2013  [31] | The fact that there was medical team there waiting for the woman gave a sense of being in control. | Faktumet att det fanns ett medicinskt team där som väntade på kvinnan gav en känsla av att vara i kontroll. |
| Sahlin et al.  2013  [31] | The following quote describes how a woman looks at the safety of the child: "I know it will be fine for the child, who will be born in a calm manner. It will go quickly and the baby will not suffer from hypoxia. Nothing bad will happen to my baby during the delivery itself. Possibly it is not as good for me, but it will go well for the baby in all cases.” | En kvinna sa: " Jag vet att det kommer gå bra för barnet, som kommer att födas på ett lugnt sätt. Det kommer gå snabbt och barnet kommer inte lida av syrebrist. Inget kommer hända mitt barn under själva förlossningen. Möjligtvis går det inte lika bra för mig, men det kommer gå bra för barnet i alla fall." |
| Sahlin et al.  2013  [31] | These quotes describe how some women look at the control and safety from the point of view: I know how it will be and also when it will happen. lt's not that it comes as an unpleasant surprise down the road that can cause complications for the baby and for me. That is the greatest incentive to make me want to have a planned caesarean section, that it is the predictability. I do not feel at all comfortable with not knowing when, and how my body will handle labour and I do not know how I would react? This is where it is going to happen during this given day and time. You would not wake up in the middle of the night and wonder what is going on and where you should go. It is more organized with a planned caesarean section. | De här citaten beskriver hur vissa kvinnor såg på kontroll och säkerhet: Jag vet hur det kommer bli och när det kommer hända. Det kommer inte komma som en otäck överraskning som kan orsaka komplikationer. Förutsägbarheten är det största incitamentet till att jag ska genomgå ett KS. Jag känner mig inte alls bekväm med att inte veta när, och hur min kropp kommer hantera förlossningen och jag vet inte hur jag kommer reagera. Här är där det kommer hända på den här givna dagen och tidpunkten. Du kommer inte vakna mitt i naten och undra vad som händer och vart du ska åka. Det är mer organiserat med ett planerat KS. |
| Weaver et al.  2007  [38] | Any risks associated with cesarean section were described as risks to the mother. | Risker vid kejsarsnitt ansågs av kvinnorna vara relaterade till modern. |
| **Sub-theme 3: Women shared that they had received relevant information about risks, but the information was not given routinely: rather, it had to be requested. The information was also perceived as contradictory or deficient, and information was sometimes collected from non-medical sources. Women with a previous CS would rather receive acceptance for their decision than information [25,27-29,32,36,37].** | | |
| **Author**  **Year**  **[Reference]** | **Meaning unit** | **Swedish translation** |
| Eide et al.  2020  [36] | One woman thought that someone should inform her about the increased risks in the forthcoming delivery and the risks and benefits of the available delivery options, given her previous CS. | En kvinna tyckte att någon skulle ha informerat henne om de ökade riskerna vid den kommande födseln och de risker, och fördelar med de olika förlossningsalternativen, givet hennes tidigare kejsarsnitt. |
| Eide et al.  2020  [36] | Lack of outreach and information from the health care system made her even more concerned about the upcoming birth. | Bristande uppföljning och information från hälso- och sjukvården gjorde henne ännu mer oroad inför den kommande födseln. |
| Eide et al.  2020  [36] | While some women felt well-informed before and during the counselling process, others expressed an unmet need for information. | Medan några kvinnor upplevde sig välinformerade innan och under beslutsprocessen uttryckte andra att deras behov av information inte tillmötesgåtts. |
| Eide et al.  2020  [36] | Some women wanted more facts presented in numbers and percentages and adapted to their specific obstetric history. | Vissa kvinnor önskade mer fakta I form av siffror och procentsatser anpassade till deras specifika obstetriska historia. |
| Eide et al.  2020  [36] | Most women had initiated the help-seeking process themselves. One of the women questioned why there was no screening or discussion of birth with women during pregnancy. | De flesta kvinnor hade initierat den hjälpsökande processen själva. En av kvinnorna ifrågasatte varför det inte fanns någon screeningprocedur eller diskussion om förlossning med kvinnor under graviditeten. |
| Eide et al.  2020  [36] | No written information was given in the decision-making process. After the decision was made women scheduled for planned CS were sent a standard information sheet about the procedure and its risks. | Ingen skriftlig information gavs under beslutsprocessen. Efter att beslut tagits skickade ett standardbrev om proceduren och dess risker, till kvinnor som var planerade för kejsarsnitt. |
| Emmet et al.  2006  [25] | This (lack of info) meant that the women could tailor the information they received to include only what they felt was relevant to them. | Kvinnor valde den information de ansåg vara relevant när informationen som gavs var bristfällig. |
| Emmet et al.  2006  [25] | Hospital doctors were described as the external influence most involved in women’s decision-making. | Förlossningsläkaren hade det största inflytandet på kvinnors beslut om förlossningssätt. |
| Emmet et al.  2006  [25] | Some women still felt ill-informed about these procedural aspects. | Några kvinnor upplevde sig dåligt informerade om procedurer kring kejsarsnitt. |
| Emmet et al.  2006  [25] | However, as several women highlighted, this also meant that to get the information they wanted they needed to know the right questions to ask. | Flera kvinnor med tidigare erfarenhet av kejsarsnitt upplevde att de behövde ställa de rätta frågorna för att få information. |
| Emmet et al.  2006  [25] | Discussion of health risks and benefits did not feature strongly in accounts of information provision. | Diskussioner om hälsorisker och fördelar kom inte fram tillräckligt tydligt i informationsförmedlingen. |
| Emmet et al.  2006  [25] | Women felt that information was not routinely provided by health professionals, but rather was available on request. | Kvinnor med tidigare erfarenhet av kejsarsnitt upplevde att de inte fick information rutinmässigt utan fick efterfråga detta. |
| Emmet et al.  2006  [25] | (health risks)…with the exception of uterine rupture, which many women recalled being discussed. | Flera av kvinnor med tidigare erfarenhet av kejsarsnitt mindes inte att risker diskuterades, med undantag från uterusruptur. |
| Emmet et al.  2006  [25] | To get the information they wanted they needed to be confident enough to ask them (the right questions). | Kvinnor med tidigare erfarenhet av kejsarsnitt ansåg att de behövde vara tillräckligt trygga att ställa de rätta frågorna, för att få den information de önskade. |
| Emmet et al.  2006  [25] | Issues around the timing of the information were also discussed, with some women feeling more information given directly after their first caesarean section would have been helpful. | Tidpunkt för att få information diskuterades och några kvinnor upplevde att information direkt efter det första kejsarsnittet hade varit önskvärt. |
| Fenwick et al.  2010  [27] | Only two were aware of possible longer-term effects. | Få kvinnor med önskemål om kejsarsnitt utan medicinsk indikation var medvetna om långtidskomplikationer efter kejsarsnitt. |
| Fenwick et al.  2010  [27] | …and only three could identify risks to the baby. | Endast tre kvinnor var medvetna om risker för barnet med kejsarsnitt. |
| Fenwick et al.  2010  [27] | Women generally described information' from the doctor stating 'that was not going to happen (to her)' (risk). | Kvinnor beskrev information från läkare som att någon risk inte kommer hända (just henne). |
| Fenwick et al.  2010  [27] | Women recalled various discussions with their doctor about the risks of a surgical birth. | Kvinnor drog sig till minnes olika diskussioner med sin läkare angående risker med kejsarsnitt. |
| Kenyon et al.  2016  [37] | …and verbal information from midwives and consultants varied. | Kvinnor upplevde att den muntliga informationen om kejsarsnitt som delgavs av personalen inte var samstämmig. |
| Kenyon et al.  2016  [37] | Missing information (and this) included both short and long-term risks and benefits of elective Caesarean section. | Kvinnor ansåg att information saknades om kortsiktiga och långsiktiga risker samt fördelar med planerat kejsarsnitt. |
| Kenyon et al.  2016  [37] | Written information was related to Caesarean section generally and not specifically for women requesting caesarean section. | Kvinnor ansåg att erhållen skriftlig information var i relaterad till kejsarsnitt i allmänhet snarare än för de med önskemål om kejsarsnitt. |
| Kenyon et al.  2016  [37] | Some women found that discussing their request for Caesarean section with their community midwife could be a difficult experience. In these cases, they found there was little clarity on the process and little information given to them to help make the decision. | Kvinnor upplevde det svårt att diskutera sina önskemål om kejsarsnitt med barnmorskan. Beslutsprocessen upplevdes oklar och informationen som bristfällig. |
| Kenyon et al.  2016  [37] | It was also agreed that there was a lack of information about what the Caesarean section surgery experience is like and of the risk information comparing vaginal birth, emergency, and elective Caesarean section and that these areas. | Kvinnor upplevde informationen som bristfällig gällande upplevelsen av en kejsarsnittsförlossning samt risker vid olika förlossningssätt. |
| Kornelsen et al.  2010  [28] | Many talked of the clarity with which their obstetricians explained the risks and benefits. | Många uttryckte att förlossningsläkarna förklarade risker och fördelar med kejsarsnitt på ett tydligt sätt. |
| Kornelsen et al.  2010  [28] | Many talked of and their (OB) willingness to answer questions in a straightforward and supportive way. | Många kvinnor uttryckte att förlossningsläkarna svarade på frågor på ett tydligt och stödjande sätt. |
| Kornelsen et al.  2010  [28] | Detailed knowledge of the range of potential risks was expressed by participants in this study from non-medical sources. | Detaljerade kunskap om potentiella risker med kejsarsnitt kom från icke-medicinska källor. |
| Kornelsen et al.  2010  [28] | Detailed knowledge of the prevalence of morbidities was expressed by participants in this study from non-medical sources. | Detaljerade kunskap om förekomst av sjuklighet efter kejsarsnitt kom från icke-medicinska källor. |
| Kornelsen et al.  2010  [28] | Detailed knowledge and alongside interpretations of knowledge of risks of vaginal delivery from non-medical sources. | Detaljerad kunskap och tolkning av risker med vaginal födsel kom från icke-medicinska källor. |
| Kornelsen et al.  2010  [28] | …popular books and magazine articles were highlighted as most influential within the decision-making process. | Böcker och tidningar hade störst påverkan i beslutsprocessen. |
| Kornelsen et al.  2010  [28] | Often women brought this information (non-medical sources) to their care providers to confirm its validity. | Ofta stämde kvinnorna av informationen från icke-medicinska källor med vårdgivarna. |
| Kornelsen et al.  2010  [28] | In such instances (bringing non-medical info), the informed consent process worked in reverse and was a matter of discussing the risks and benefits of different modes of delivery. | Avstämning av informationen från icke-medicinska och informerat samtycke handlade om att diskutera risker och fördelar med olika förlossningssätt. |
| McGrath et al.  2009  [29] | Mothers may not even read the information provided if their minds are already made up. | Kvinnor valde ibland att inte läsa erhållen information om de redan hade bestämt sig för kejsarsnitt. |
| McGrath et al.  2009  [29] | The health professionals may not even offer information if they think the mother is predetermined in her choice. | Kvinnor upplevde att de inte alltid erbjöds information om personalen trodde att de var övertygade i sitt val av förlossningssätt. |
| McGrath et al.  2009  [29] | With regard to the risks associated with different modes of delivery, the process was not simply one of the health professionals providing information that was absorbed and acted on by the mother. | Med hänsyn till risker associerade med olika förlossningssätt bestod beslutsprocessen inte enbart av att personalen informerade och att kvinnorna tog till sig informationen och agerade utifrån den. |
| McGrath et al.  2009  [29] | Again, the important point from the perspective of this group of mothers, who were committed to having an EC, is that it was not the information per se but rather the way it was communicated that affirmed the mother’s pre-determined choice. | Omföderskor med tidigare erfarenhet av kejsarsnitt och hade önskemål om planerat kejsarsnitt ansåg att informationen i sig inte var det viktigaste utan det var hur informationen kommunicerades som bekräftade deras förutbestämda önskemål. |
| Thirukumar et al. 2021 [32] | To make a MOB decision, most women relied on the information they received from health-care staff and midwives (n = 29) and trusted this information. | I beslutet av förlossningssätt litade de flesta kvinnorna på informationen som gavs av personalen. |
| Thirukumar et al. 2021 [32] | However, while most women looked to their clinician for information and guidance, many supplemented this with advice from friends and family members (n = 16), their own previous experience (n= 7), and their own research (n = 21). When conducting their own research, they looked online (n =21) for journal articles and forums and read books (n = 1). | Förutom information och vägledning från personalen så sökte många av kvinnorna råd från vänner och familjemedlemmar i beslutsprocessen av förlossningssätt. Kvinnornas tidigare erfarenheter, informationssökning via artiklar publicerade digitalt, forum på Internet och information via böcker guidade kvinnorna i beslut av förlossningssätt. |
| Thirukumar et al. 2021 [32] | Some women appreciated how the online research could provide detail to the hospital-provided information | Några kvinnor uppskattade att eftersöka information på Internet som komplement till den sjukhusbaserade information om förlossningssätt. |
| Thirukumar et al. 2021 [32] | Other women were against online research, in fear of reading contradictory or confusing information, and thus primarily utilized the trusted hospital-provided information: | Andra kvinnor motsatte sig att söka information på nätet i rädsla för att erhålla motsägande eller förvirrande information om förlossningssätt, och primärt litade de på den betrodda sjukhusbaserade informationen. |
| Thirukumar et al. 2021 [32] | So I only got the information from the hospital staff. I did not do any research at all on my own because . . . I was afraid of reading fake news about it. So I just asked the midwives. (Janet, requested repeat CB) | Omföderska med kejsarsnittserfarenhet och som önskat planerat kejsarsnitt sa att den enda informationen som hon hade tagit del av var från sjukhuspersonalen. Hon hade efterfrågat information av barnmorskorna då hon var rädd för att få felaktig information om hon själv skulle ha sökt information om förlossningssätt. |
| Thirukumar et al. 2021 [32] | I think there's a lot of information out there and you can look. And everyone's got an opinion and I wanted to minimize it as much as possible so I think I tried to keep as small a circle of engagement as I could. (Mary, requested due to previous traumatic CB) | Kvinna med tidigare traumatisk förlossningsupplevelse ansåg att det finns mycket information kring kejsarsnitt. Då alla har en åsikt om kejsarsnitt så efterfrågade hon information endast från en liten grupp av människor gällande hennes önskemål om planerat kejsarsnitt. |
| Thirukumar et al. 2021 [32] | Some women also got information from childbirth classes (n = 6), but this did not always address their information needs | Några kvinnor fick information via förberedande förlossningskurser, men dessa kurser adresserade inte alltid deras informationsbehov om förlossningssätt. |
| Thirukumar et al. 2021 [32] | The majority of women described positive experiences of decision-making with their obstetrician and/or midwife (Table 2), and reported that they were given the required information (n = 19) and felt listened to (n = 10). | De flesta av kvinnorna hade en positiv upplevelse av beslutsprocessen av förlossningssätt i samtalet med obstetrikern och/eller barnmorskan. Kvinnorna ansågs ha fått tillräcklig information och att de blev lyssnade på i beslutsprocessen av förlossningssätt. |
| Thirukumar et al. 2021 [32] | Comments such as "I received the information I needed" were common. | Flera kvinnor som fött barn med planerat kejsarsnitt beskrev att de hade fått den information de behövde kring förlossningssätt under graviditet. |
| Thirukumar et al. 2021 [32] | "I was very comfortable and very much listened to by my doctor" were common. | Flera kvinnor som fött barn med planerat kejsarsnitt var trygga och upplevde sig lyssnade på av sin läkare i beslutsprocessen av förlossningssätt. |
| Thirukumar et al. 2021 [32] | However, while many women reported receiving the information they needed to make informed decisions, others reported feeling pressured into having or not having a CB (n = 11), or conversely being expected to make a decision by themselves with little information or guidance from medical staff. | Emedan många kvinnor beskrev att de hade fått tillräcklig information för att göra ett informerat val kring förlossningssätt så ansåg andra kvinnor att de var tvungna att acceptera eller inte acceptera planerat kejsarsnitt. Andra kvinnor var tvärtemot förväntade att ta egna beslut om förlossningssätt utifrån begränsas information eller vägledning från medicinsk personal. |
| Thirukumar et al. 2021 [32] | These women emphasized the importance of clinicians maintaining a neutral stance when facilitating decision-making. | Kvinnor gav eftertryck till vikten av att personal ska inta en neutral ståndpunkt i beslutsprocessen av förlossningssätt. |
| Thirukumar et al. 2021 [32] | Women also reported that they were left to make their own decision, with little guidance from medical staff (n = 4) or access to evidence-based information to help inform their decision (n = 8). | Kvinnor beskrev också att de blev lämnade att själva besluta om förlossningssätt utifrån begränsad vägledning av medicinsk personal eller tillgång till evidensbaserad information som hjälp för att göra ett informerat val. |
| Thirukumar et al. 2021 [32] | To be able to make informed decisions, women valued recommendations from clinicians based on the best available evidence. | För att kunna göra ett informerat val av förlossningssätt så uppskattade kvinnorna om personalen gav rekommendation utifrån den bästa tillgängliga evidensen. |
| Thirukumar et al. 2021 [32] | For many, the information they had been given was "too simple and basic" (n = 9), often presented in pamphlets, and they would have preferred an in-depth discussion of how the information was personally applicable. | Flertalet kvinnor ansåg att de hade fått en för enkel och för begränsad information om förlossningssätt som oftast presenterades via broschyr. Kvinnorna hade föredragit att få individanpassad och detaljerad information via diskussioner. |
| Thirukumar et al. 2021 [32] | I could have had more information. Well ...actually I think I just wanted the information to apply to me. I just wanted it to apply to me. (Lauren, requested repeat CB) | Omföderska med kejsarsnittserfarenhet som efterfrågat planerat kejsarsnitt beskrev att hon kunde ha fått mer information om förlossningssätt men hon hade helst önskat individanpassad information utifrån henne situation. |
| Thirukumar et al. 2021 [32] | In relation to the actual information, women would have liked, women commented that staff spent the majority of the consultation explaining the actual procedure, and that they would have liked more information in relation to how the birth would progress (n = 4), skin-lo-skin contact (n = 3), MOB options (n = 5), pain relief (n = 7), and recovery (n= 10). | Kvinnorna skulle ha föredragit att konsultationen om förlossningssätt framförallt hade innefattat information om förlossningssätt, förlossningsprogress, hud-mot-hud kontakt med barnet, val av förlossningssätt, smärtlindring och återhämtning efter förlossning. |
| Thirukumar et al. 2021 [32] | Multiple women voiced that the information they were given antenatally, did not line up with unexpected events that occurred during birth and recovery. | Flera kvinnor som genomgått planerat kejsarsnitt beskrev att information given under graviditet inte inkluderade oväntade händelser under förlossning och återhämtning efter förlossningen. |
| Thirukumar et al. 2021 [32] | I probably didn't understand the post-recovery enough, and what happens after the 6 weeks. I knew that it had a 6 week recovery time but I probably didn't understand how major the surgery actually was in terms of just you know, slicing you open, dicing and then shoving your abdominals back together? {. .. ] Recovery from natural/ labor is different from recovery from a c-section. (Adrienne, previous traumatic VB) | Omföderska med tidigare traumatisk vaginal förlossning sa att hon troligtvis inte hade förstått innebörden av återhämtningsperioden och vad som händer under de första 6 veckorna efter ett planerat kejsarsnitt. Hon visste att återhämtningsperioden skulle vara 6 veckor efter kejsarsnittet men förstod inte fullt ut hur omfattande kirurgin egentligen var, omfattningen av att bli uppskuren, sedan öppna och därefter återställa bukmusklerna. Återhämtning efter en naturlig förlossning ansågs skilja sig åt från kejsarsnitt. |
| **Theme 2:**  **Women’s rights to receive support and acceptance for a CS request** | | |
| ***Women considered it both important and their right to receive support and acceptance for their request for a CS. Many women received support from clinicians for their decision to have a CS, sometimes after negotiation. Sometimes a woman’s request for CS was denied, and sometimes the decision process was regarded as frivolous and lacked support. Women often had to repeat and defend their (in their eyes) well-motivated decisions about the mode of birth. Some women renegotiated their attitude toward mode of birth after previous birth experiences or professional support in the decision-making process.*** | | |
| **Sub-theme 1: Women had the opinion that their motives were well-founded and that they actually had medical risk factors that were indications for CS. They wanted to be in control and feel safe, and they viewed CS as the only option, as they believed that they were unable to give birth vaginally. The women also emphasized their inviolable right to self-determination over their bodies and their mode of birth, although some women raised the problems with complete autonomy [25-28,31,35,36,38].** | | |
| **Author**  **Year**  **[Reference]** | **Meaning unit** | **Swedish translation** |
| Eide et al.  2019  [35] | Several women based their request on what they personally considered medical risk factors. They were concerned about complicated births running in their families, previous protracted labor/emergency CS, perception of having a narrow pelvis or expecting a big baby. | Flera omföderskor med önskemål om kejsarsnitt utan medicinsk indikation baserade sina önskemål på, vad de själva ansåg var, medicinska riskfaktorer. |
| Eide et al.  2019  [35] | The conviction of not being able to deliver vaginally was recurring. | Gravida kvinnor med önskemål om kejsarsnitt utan medicinsk indikation var övertygade om att de var oförmögna att föda vaginalt. |
| Eide et al.  2020  [36] | Women’s views on autonomy were divided. A minority of the women thought the final choice should be taken by the woman herself. | Kvinnors syn på autonomi var tudelad. En minoritet av kvinnorna ansåg att det slutgiltiga beslutet skulle tas av kvinnan själv. |
| Eide et al.  2020  [36] | Arguments presented were: It was her body and should be her decision, she knowns her own body and psyche the best, she was the one bearing the consequences and the outcome of an attempt for VD was uncertain. | De argument som presenterades var: det var hennes egen kropp och beslutet borde vara hennes, hon känner sin kropp och psyke bäst, hon är den som bär konsekvenserna vid ett osäkert försök att föda vaginalt. |
| Eide et al.  2020  [36] | The fact that it was a surgical procedure, with elevated risks for the mother and child, a medical choice, a possibility that women would have CS for reasons of convenience or because it was misunderstood as an ‘easy way out, were arguments presented for why complete autonomy would be problematic | Det faktum att det är en kirurgisk procedur, med ökade risker för mor och barn, ett medicinskt val, en möjlighet att kvinnor vill ha ett kejsarsnitt av bekvämlighetsskäl eller missförstånd, som ’en lätt väg ut’, var argument som presenterades när det gällde varför komplett autonomi skulle vara problematisk. |
| Emmett et al.  2006  [25] | The certainty of women who wanted an elective caesarean section was a lack of confidence that a vaginal delivery was possible for them. | Omföderskors övertygelse om kejsarsnitt var grundad i bristande tillit och trygghet inför vaginal födsel. |
| Fenwick et al.  2006  [26] | Many women believed they had no option. | Många kvinnor upplevde att de inte hade någon annan möjlighet än att föda med kejsarsnitt. |
| Fenwick et al.  2006  [26] | Many women believed they had no choice. | Många kvinnor upplevde att de inte hade någon valmöjlighet när det gällde förlossningssätt. Kejsarsnitt var det enda alternativet. |
| Fenwick et al.  2010  [27] | The women reported that as it was their choice (CSMR). | Kvinnor rapporterade att kejsarsnitt utan medicinsk indikation var deras eget val. |
| Kornelsen et al.  2010  [28] | Participants perceived the choice of CS to be their inviolable reproductive rights. | Deltagarna ansåg att valet av kejsarsnitt hörde till deras okränkbara reproduktiva rättighet. |
| Kornelsen et al.  2010  [28] | Participants perceived the choice of CS to be their inviolable consumer rights. | Kvinnor ansåg att valet av kejsarsnitt hörde till kvinnors konsumenträttighet. |
| Kornelsen et al.  2010  [28] | For many of the participants in this study, the fact that it was even possible to request a Caesarean section without a medical indication represented forward progress in women’s reproductive rights. | För många deltagare representerade faktumet att man kan önska kejsarsnitt utan medicinsk indikation ett framsteg för kvinnors reproduktionsrättigheter. |
| Kornelsen et al.  2010  [28] | As this consumer right had become the baseline expectation for participants, some were critical of hospitals that had chosen not to allow the procedure. | Deltagarna förväntade sig en konsumenträttighet att få kejsarsnitt. Vissa deltagare var kritiska mot sjukhus som hade valt att inte tillåta ingreppet. |
| Sahlin et al.  2013  [31] | "It feels like you have to have a medical explanation for a caesarean section." | En kvinna sa att det känns som om man måste ha en medicinsk orsak för att få ett kejsarsnitt. |
| Sahlin et al.  2013  [31] | Several women in the study stated that it is up to every single individual to decide mode of delivery. | Flera deltagare konstaterade att det är upp till varje enskild individ att bestämma förlossningssätt. |
| Sahlin et al.  2013  [31] | Many of them expressed that it is a modern way of thinking. | Många av dem sa att det är att tänka på ett modernt sätt. |
| Sahlin et al.  2013  [31] | Several of the interviewed women said that you are able to choose almost anything in life and there for it is your privilege to choose whether you want a vaginal delivery or a caesarean section. | Flera deltagare sa att man kan välja nästan allting i livet och därför är det ditt privilegium att välja förlossningssätt. |
| Sahlin et al.  2013  [31] | "And I think that in today's society in advocating individualism, as it were, it is obvious that it will spread in maternity care that people will demand that I want it like this or like this." | En kvinna sa: "Jag tänker att i dagens samhälle där individualism förespråkas, är det tydligt att detta tankesätt kommer spridas till förlossningsvården och att människor kommer kräva att de vill ha det på det ena eller det andra sättet." |
| Sahlin et al.  2013  [31] | "It is my body and if I want you to cut into me even though I am perfectly healthy, then you should do it I think." | ”Det är min kropp och om jag vill att du ska skära upp mig även om jag är fullt frisk, så tycker jag att du ska göra det” |
| Sahlin et al.  2013  [31] | Almost all stated that the reason for their wish for a caesarean section is not about fear of giving birth, it is deeper than that. | Nästa alla deltagare konstaterade att orsaken till deras önskemål var något som är djupare än förlossningsrädsla. |
| Weaver et al.  2007  [38] | Two interview participants described wanting to ask for cesarean section to have some control over the timing of the birth. | Två deltagare beskrev att de ville efterfråga kejsarsnitt för att ha kontroll över tidpunkten för förlossningen. |
| Weaver et al.  2007  [38] | The 8 women who asked for and underwent caesarean section believed that they had clinical justifications for the operation. | Kvinnor ansåg sig ha kliniskt motiverad orsak för kejsarsnittet. |
| **Sub-theme 2:**  **Women sometimes perceived unconditional support or encouragement for their birth preferences, but they also faced lack of engagement and time during consultations, feeling questioned, or denial of their CS request. Support from clinicians was important and could lead to women daring to give birth on their own terms. The decision-making process was perceived as inaccurate or unserious when women were forced to undergo counselling, which made them feel condemned, violated, or pressured; this could also occur when the decision about mode of birth was made late in the pregnancy, when documentation of their motives for CS was incomplete, or when it mirrored the clinicians’ own preferences [25,27-32,36-38].** | | |
| **Author**  **Year**  **[Reference]** | **Meaning unit** | **Swedish translation** |
| Eide et al.  2020  [36] | Many felt included in the decision-making, either by being able to make the final choice for themselves or having the opportunity to say no to a vaginal birth. | Många kände sig inkluderade I beslutsprocessen, aningen genom att ta det avgörande beslutet själva eller få möjligheten att säga nej till vaginal födsel. |
| Eide et al.  2020  [36] | Other felt as if they were presented to a judge or committee of doctors evaluating their case, without being present to defend themselves or being able to influence the decision. | Andra kände det som att de stod inför en domare eller en kommitté av läkare som utvärderade deras skäl, utan att få möjligheten att försvara sig eller påverka beslutet. |
| Eide et al.  2020  [36] | Most women would prefer a shared process between the woman and caregiver or a conditional autonomous choice depending on reason for the request, where ungrounded requests could be denied. | De flesta kvinnor skulle föredra en gemensam process mellan kvinnan och personalen eller en villkorad autonom valmöjlighet, beroende på orsaken till kraven, där ogrundade krav skulle förvägras. |
| Eide et al.  2020  [36] | Many women experienced the accessibility of counselling for their caesarean request to be challenged by late referrals to counselling, a strong ideal of vaginal delivery, a long-lasting process of and late decision-making. | Många kvinnor upplevde tillgången till rådgivning för deras kejsarsnittsönskemål som utmanande genom sen remittering, ett starkt ideal för vaginal födsel, en långdragen process och ett sent beslut. |
| Eide et al.  2020  [36] | Several women had the impression that caregivers in primary and specialized care usually advocated for VD. For some it appeared less trustworthy, and lacking of neutral ground. | Många kvinnor hade känslan av att vårdgivare inom primär-och specialiserad vård vanligtvis argumenterade för vaginal födsel. För några upplevdes detta som mindre trovärdigt och som att det saknades ett neutralt förhållningssätt. |
| Eide et al.  2020  [36] | There was a common understanding among women that the clinic was very restrictive in its policy towards CS. | Det var vanligt att kvinnor ansåg att kliniken var väldigt restriktiva I sin policy gentemot kejsarsnitt. |
| Eide et al.  2020  [36] | A common complaint among women was that the birth counselling process was too long, and that the decision was made too late in pregnancy, escalating psychological stress and uncertainty during pregnancy. | Ett vanligt klagomål bland kvinnor var att rådgivningsprocessen var för lång och att beslutet togs allt för sent, vilket ökade den psykologiska stressen och osäkerheten under graviditeten. |
| Eide et al.  2020  [36] | Prior to counselling, many feared they would not be understood or taken seriously, and many were relieved to find the opposite. | Innan rådgivningen var många rädda att de inte skulle bli förstådda eller tagna på allvar men blev lättade när de upplevde det motsatta. |
| Eide et al.  2020  [36] | Several women were not able to enjoy their pregnancy until the decision on delivery mode was taken. | Många kvinnor kunde inte njuta av graviditeten innan beslutet om förlossningssätt hade tagits. |
| Eide et al.  2020  [36] | Women were generally pleased with the birth counselling provided by midwives. They usually felt seen, heard, respected, and trusted on their stories. They appreciated going through previous birth records and cleaning up misunderstanding and questions. | Kvinnor var vanligtvis nöjda med förlossningsrådgivningen de erhöll av barnmorskor. De kände sig sedda, hörda, respekterade och trodda. De uppskattade att gå igenom tidigare förlossningsjournaler och reda ut missförstånd och frågor. |
| Emmett et al.  2006  [25] | Generally, health professionals were described as being accepting or supportive of the woman’s decision whichever type of delivery she chose. | Omföderskor med tidigare erfarenhet av kejsarsnitt ansåg att personalen var i allmänhet accepterande eller stödjande, oavsett förlossningssätt. |
| Emmett et al.  2006  [25] | The majority of women felt that their health professionals had allowed them to make their own choice about mode of delivery. | De flesta omföderskorna med tidigare erfarenhet av kejsarsnitt upplevde att personalen tillät dem att själva besluta om förlossningssätt. |
| Emmett et al.  2006  [25] | The role of some community midwives was very helpful. | Omföderskor med tidigare erfarenhet av kejsarsnitt uppfattade att vissa barnmorskor var hjälpsamma i beslutet av förlossningssätt. |
| Emmet, et al.  2006  [25] | Often, this preference (to try vaginal birth) was not explicitly stated by the health professionals, but rather was something women perceived. | Vårdgivares preferenser för vaginal födsel var inte alltid uttalat, men uppfattades ofta av omföderskor med tidigare erfarenhet av kejsarsnitt. |
| Emmett et al.  2006  [25] | For some, the uncertainty resulted from conflicting opinions about the best choice, among their health professionals. | Omföderskors osäkerhet angående förlossningssätt bottnade i motstridiga åsikter om förlossningssätt mellan personalen. |
| Emmett et al.  2006  [25] | Others (community midwives) provided information contradictory to the hospital doctor. | Barnmorskor upplevdes ge motstridig information om förlossningssätt jämfört med läkarna. |
| Emmett et al.  2006  [25] | The role of community midwives … some seemed reluctant to get involved at all. | Vissa barnmorskor upplevdes av omföderskorna som ovilliga att engagera sig i beslutsfrågan om förlossningssätt. |
| Emmett et al.  2006  [25] | One felt that health professionals had ignored her repeated requests for an elective caesarean section. | En omföderska med tidigare erfarenhet av kejsarsnitt upplevde att hennes återkommande önskemål om kejsarsnitt ignorerades av personalen. |
| Emmett et al.  2006  [25] | Ignored requests for an elective caesarean section and that she would be forced to have a VBAC. | Det av personalen ignorerade önskemålet om kejsarsnitt upplevdes av omföderskan med tidigare erfarenhet av kejsarsnitt som att bli tvingad att föda vaginalt. |
| Emmet, et al.  2006  [25] | However, several women felt that health professionals really prefer women to try to deliver vaginally. | Många omföderskor med tidigare erfarenhet av kejsarsnitt upplevde att personalen ansåg att kvinnor borde försöka föda vaginalt. |
| Emmett et al.  2006  [25] | In contrast to the majority, two participants felt they were not allowed to choose their delivery method. | I kontrast till de flesta informanter, så tilläts inte två gravida kvinnor välja förlossningssätt. |
| Fenwick et al.  2010  [27] | Women reported that doctors made no enquiry about why they had made this request. | Kvinnor rapporterade att läkarna inte efterfrågade deras orsaker för önskemål om kejsarsnitt. |
| Fenwick et al.  2010  [27] | Participants perceived that the doctor was covering themselves legally (by CS). | Kvinnorna ansåg att läkare skyddade sig själva lagligt genom att samtycka till kejsarsnitt. |
| Fenwick et al.  2010  [27] | Participants perceived that it (CSMR) was convenient for them (doctors). | Förstföderskor uppfattade att kejsarsnitt utan medicinsk indikation var bekvämt för läkarna. |
| Fenwick et al.  2010  [27] | There was also a perception that it was good business. | Kvinnor uppfattade att kejsarsnitt utan medicinsk indikation var en bra ekonomisk affär för läkarna. |
| Fenwick et al.  2010  [27] | Women reported that their doctors readily acceded to the request for a caesarean section in their first pregnancy. | Kvinnor rapporterade att läkarna lätt accepterade deras krav på kejsarsnitt. |
| Fenwick et al.  2010  [27] | Women reported that their doctors were even encouraging of the choice for a caesarean section. | Förstföderskor med önskemål om kejsarsnitt utan medicinsk indikation rapporterade att läkarna till och med uppmuntrade deras val av kejsarsnitt. |
| Kenyon et al.  2016  [37] | If women were sent to discuss their decision with the Consultant Midwife when they were already sure that they had made the right decision, they could find the consultation upsetting if they felt that they were being pressurized into changing their mind, rather than the supportive process that was intended. | Att bli remitterad till (konsult)barnmorska för diskussion om önskemålet om kejsarsnitt upplevdes upprörande när kvinnorna ansåg att de redan tagit ett väl avvägt beslut. Kvinnorna upplevde sig pressade att ändra uppfattning istället för att få ett avsett stöd i beslutsprocessen. |
| Kenyon et al.  2016  [37] | Women felt that those health care professionals were, at all stages, judging or stereotyping them, when in fact their decision was a carefully thought through, and sometimes very difficult, decision. | Kvinnor upplevde sig bli dömda och stereotypt behandlade trots att deras beslut om kejsarsnitt var noga genomtänkt och ibland svårt att ta. |
| Kenyon et al.  2016  [37] | Some felt that their midwife was judging them for their decision, which compromised the relationship. | Några kvinnor upplevde barnmorskan som fördömande gällande deras beslut om kejsarsnitt vilket ledde till ett komprometterat förhållande. |
| Kenyon et al.  2016  [37] | The feelings of being judged for their decision continued after the birth in interactions with health care professionals. | En känsla av att bli dömda för beslutet om kejsarsnitt fortsatte även efter förlossningen. i möten med vårdpersonal. |
| Kenyon et al.  2016  [37] | The effect a delayed decision had on experience of  pregnancy was to cause unnecessary anxiety. | Kvinnor upplevde att ett sent beslut om förlossningssätt under graviditet skapade onödig oro inför förlossningen. |
| Kenyon et al.  2016  [37] | Only one participant was referred to the mental health services and she felt that it was an inappropriate referral. | Att bli remitterad till mental health services (stöd vid mental ohälsa) upplevdes olämpligt när kvinnan var säker i sitt beslut om kejsarsnitt. |
| Kenyon et al.  2016  [37] | They also felt that long term risk information was used to ram home risks. | Kvinnor upplevde att personalen ville skrämmas då de informerade om risker för långtidskonsekvenser med kejsarsnitt. |
| Kenyon et al.  2016  [37] | If women were sent to discuss their decision with the Consultant Midwife when they were already sure that they had made the right decision, they could find the consultation unnecessary, rather than the supportive process that was intended. | Att bli remitterad till en barnmorska för diskussion om önskemålet om kejsarsnitt upplevde kvinnorna som onödigt när de var säkra på deras beslut var rätt. Fick detta istället för att få stöd i beslutsprocessen som var avsikten med att bli remitterad. |
| Kenyon et al.  2016  [37] | Other themes that emerged from the discussion included the way that a woman’s decision to have caesarean section was recorded (…) the lack of detailed recording of discussion between women and health care professionals when requesting caesarean section. | Det fanns en brist på detaljerad dokumentation av diskussionerna mellan kvinnan och vårdpersonalen. |
| Kornelsen et al.  2010  [28] | Ultimately, what was most important to the participants was the sense of support they felt for their decision to have a caesarean section. | I slutändan, så var det viktigaste för förstföderskor med önskemål om planerat kejsarsnitt att få stöd av personalen i sitt beslut om att få ett planerat kejsarsnitt. |
| Kornelsen et al  2010  [28] | Many spoke positively of their care providers’ support for the decision, which was described in terms of reassurance and cooperation. | Personalens stöd för förstföderskors önskemål om planerat kejsarsnitt beskrevs positivt av kvinnorna såsom att de fick uppmuntran och att de hade ett gott samarbete. |
| Kornelsen et al.  2010  [28] | Although all participants felt well-supported by their obstetricians, about their desire to give birth by caesarean section. | Alla deltagare upplevde gott stöd av förlossningsläkarna i sin önskan om planerat kejsarsnitt. |
| Kornelsen et al.  2010  [28] | Some participants received support for caesarean section from their care providers, on the basis of non-indicated physiological conditions. | Några deltagare fick stöd från personal för deras önskemål om planerat kejsarsnitt utan medicinsk indikation. |
| Kornelsen et al.  2010  [28] | For all participants, the informed consent process was confirmatory in their decision-making process. | Alla förstföderskorna med önskemål om planerat kejsarsnitt ansåg att samtyckesprocessen bekräftade deras önskemål. |
| Kornelsen et al.  2010  [28] | Some women noted that care providers’ positive descriptions of the surgery reinforced their decision to give birth by PIECS. | Personalens positiva beskrivningar av kejsarsnittsoperationen förstärkte några av förstföderskornas beslut om planerat kejsarsnitt. |
| Kornelsen et al.  2010  [28] | Participants felt that physicians who declined to support delivery by PIECS were obstructive. | Läkare som nekade kejsarsnitt upplevdes ohjälpsamma |
| Kornelsen et al.  2010  [28] | Participants felt that physicians who declined to support delivery by PIECS unsupportive. | Läkare som nekade kejsarsnitt upplevdes icke-stödjande. |
| Kornelsen et al.  2010  [28] | Others noted that their physicians were reluctant to support a caesarean section on the basis of non-indicated physiological conditions. | Andra deltagare noterade att läkare var motvilliga till att stödja kejsarsnitt utan medicinsk indikation. |
| Kornelsen et al.  2010  [28] | Others noted their (family) physician’s reluctance to condone the procedure in the absence of reasons for it, causing conflict in the informed consent process. | Familjeläkares motvillighet att agera utifrån förstföderskornas önskemål om kejsarsnitt orsakade konflikt i beslutsprocessen av förlossningssätt. |
| Kornelsen et al.  2010  [28] | Their reluctance to condone an elective Caesarean section without a medical indication was perceived as obstructive. | Familjeläkares motvillighet att agera genom att remittera utifrån förstföderskornas önskemål om kejsarsnitt upplevdes hindrande. |
| Kornelsen et al.  2010  [28] | Several noted the lack of support within the decision-making process they felt from their family physicians about their desire to give birth by caesarean section. | Flera deltagare upplevde bristande stöd av familjeläkare i sin önskan om kejsarsnitt. |
| Kornelsen et al.  2010  [28] | For some respondents the informed consent process was less rigorous than they anticipated. | Beslutsprocessen om förlossningssätt upplevdes mindre rigorös än förväntat av några deltagare. |
| Kornelsen et al.  2010  [28] | One compared it to the more stringent process of consenting to an amniocentesis and noted her surprise over the lack of comparable decision points. | En deltagare blev förvånad över att samtyckesprocessen för att genomgå fostervattenprov var mer stringent än denna process. |
| Kornelsen et al.  2010  [28] | The perceived lack of rigour in the informed consent process may have been due in part to the high level of awareness that all participants had regarding caesarean section. | Den upplevda avsaknaden av noggrannhet i det informerade valet (beslutsprocessen) förklarade förstföderskorna med deras medvetenhet om kejsarsnitt. |
| Kornelsen et al.  2010  [28] | For most of the participants the process of decision making was marked by informed consent discussions with their care provider(s), understanding the evidence through research, and support from their physicians. For all participants in this study the informed consent process was confirmatory in their decision-making process, as opposed to challenging. | Samtyckesprocessen var för samtliga deltagare bekräftande i sina beslut, dvs den utmanade inte deltagarna i deras beslut. |
| McGrath et al.  2009  [29] | Participants made clear statements that their GPs took time to discuss the birthing choice and were supportive of their decisions. | Kvinnor med tidigare erfarenhet av kejsarsnitt och som hade önskemål om planerat kejsarsnitt upplevde att allmänläkaren tog sig tid att diskuskutera förlossningssätt och att de gav stöd i deras önskemål om kejsarsnitt. |
| McGrath et al.  2009  [29] | However, even when the GP provided information on both options it was the support for the mother’s pre-determined choice that was considered most important. | Kvinnor med tidigare erfarenhet av kejsarsnitt och hade önskemål om planerat kejsarsnitt ansåg det viktigast att få stöd av allmänläkaren i sitt förutbestämda önskemål om förlossningssätt än att få information om båda förlossningssätten (vaginal förlossning och kejsarsnittsförlossning). |
| McGrath et al  2009  [29] | Support from the GP for a birthing option could be expressed in statements indicating that an EC was the only option. | Kvinnor med tidigare erfarenhet av kejsarsnitt och hade önskemål om planerat kejsarsnitt upplevde att de fick stöd i önskemålet av sin allmänläkare när kejsarsnitt framställdes som det enda alternativet. |
| McGrath et al. 2009  [29] | The mothers expressed satisfaction with this support for their pre-determined choice and expressed that it was this support, rather than information or discussing on different risks for alternative options, that was sought from the GP. | Kvinnor med tidigare erfarenhet av kejsarsnitt och hade önskemål om planerat kejsarsnitt var nöjda med att få stöd i sin förutbestämda åsikt av allmänläkaren, istället för att få information eller diskutera kring risker med olika förlossningssätt. |
| McGrath et al.  2009  [29] | Central was the obstetrician’s acceptance of the mother’s choice of EC. | Kvinnor ansåg att det viktigaste var att obstetrikern accepterade önskemålet om kejsarsnitt. |
| McGrath et al.  2009  [29] | Overall, the midwives were seen as supportive of the mother’s desire for an EC. | Omföderskor med tidigare erfarenhet av kejsarsnitt och hade önskemål om planerat kejsarsnitt ansåg att barnmorskorna gav stöd för deras önskemål om kejsarsnitt. |
| McGrath et al. 2009  [29] | Some of the midwives even used their own experience to affirm the choice of an EC. | Några barnmorskor bekräftade kvinnornas önskemål om kejsarsnitt genom att beskriva personliga preferenser för kejsarsnitt. |
| McGrath et al.  2009  [29] | One mother feared her request for an EC would not be heeded because of a previous difficult experience at another hospital. | En omföderska med tidigare erfarenhet av kejsarsnitt upplevde rädsla för att önskemålet om kejsarsnitt inte skulle beaktas av personalen. |
| McGrath et al.  2009  [29] | Alternatively, the GP could project their personal preferences onto the mother in a way that was deemed supportive by the mother. | Kvinnor upplevde att allmänläkaren projicerade personliga åsikter som stöd för deras val av kejsarsnitt. |
| McGrath et al. 2009  [29] | A few of the participants noted that the hospital obstetricians did present the option of VBAC positively and sensitively. | Ett fåtal deltagare upplevde att obstetrikerna presenterade vaginal förlossning på ett positivt och sympatiskt sätt. |
| McGrath et al.  2009  [29] | There is ample evidence that the midwives are proactive in positing the idea of the value of attempting a VBAC without unduly pressuring the mothers. | Kvinnor upplevde att barnmorskor tydligt uppmuntrade vaginal förlossning utan tvång. |
| McGrath et al.  2009  [29] | …advice against a VBAC was couched in positive terms of the convenience of an EC. | Kvinnor ansåg att personalens avrådan inför vaginal födsel var beskrivet i termer av bekvämlighet av kejsarsnitt |
| Ramvi et al.  2011  [30] | She made an appointment with a gynecologist to help make the decision but was told that she could not have a cesarean section. | En förstföderska önskade samtal med gynekolog för att få hjälp med beslut om förlossningssätt men blev enbart tillsagd att kejsarsnitt inte var aktuellt. |
| Ramvi et al.  2011  [30] | She wrote a letter to the hospital requesting one, but the application was rejected. She felt that she was not taken seriously by the gynaecologist. | En förstföderska med önskemål om kejsarsnitt upplevde sig inte bli tagen på allvar av gynekolog då kejsarsnitt inte beviljades. |
| Ramvi et al  2011  [30] | She wondered if she would be able to have more children in Norway due to the fact that she wanted a cesarean section. | Hon ifrågasatte sig själv, efter förlossningen, om det var möjligt att föda fler barn då inte kejsarsnitt beviljades. |
| Ramvi et al.  2011  [30] | During her pregnancy, she was referred to the Team Midwifery project because she wanted to have a cesarean section. Had the understanding that she would not be forced to give birth and she upheld her request for a cesarean section. | En kvinna blev hänvisad till stödsamtal med barnmorska på grund av önskan om kejsarsnitt. Hon hade inte förväntat sig tvång till vaginal födsel. |
| Ramvi et al.  2011  [30] | The women’s experience of infringement in their relationships with professionals. They all experienced a lack of respect and mutuality. | Kvinnor upplevde sig bli kränkta i mötet med personalen. Kvinnorna upplevde brist på respekt för önskemålet om kejsarsnitt och samförstånd med personalen. |
| Ramvi et al.  2011  [30] | Kristine began to think that a vaginal birth was possible if I could have it on my own terms, have the same midwife present during the birth. | Kristine började tro att vaginal födsel var möjligt om detta kunde ske på egna premisser, genom att få samma barnmorska närvarande under födandet. |
| Ramvi et al.  2011  [30] | And have a set date for the birth. All of this was crucial to say that she had remembered the birth and that she had had a wonderful birth. | En kvinna började tro att vaginal födsel var möjligt om detta kunde ske på egna premisser, att få ett planerat datum för födseln. Allt detta var avgörande för positiv förlossningsupplevelse. |
| Sahlin et al.  2013  [31] | The vast majority of the women described that they received a good response from the health­care providers during the current experience of the decision making in pregnancy. | De allra flesta förstföderskorna beskrev att de fick bra respons från vårdpersonalen i beslutsprocessen under graviditeten. |
| Sahlin et al.  2013  [31] | They feel that they got support in their wish for a caesarean section. | Deltagarna kände att de fick stöd i sina önskemål om kejsarsnitt. |
| Thirukumar et al. 2021 [32] | Another common concern regarded insufficient time during medical consultations. | En vanligt förekommande oro handlade om otillräcklig tid erbjudet för medicinska konsultationer. |
| Thirukumar et al. 2021 [32] | Women frequently compared midwifery appointments to doctor's appointments and said they felt more comfortable to have in-depth discussions and ask questions in the former (n = 6). | Kvinnorna ofta jämförde barnmorskebesök med läkarbesök. De beskrev sig mer bekväma att diskutera och ställa frågor till barnmorskor. |
| Thirukumar et al. 2021 [32] | Women suggested that communication might be improved through enhanced medical continuity of care (n = 9). | Kvinnor föreslog att kommunikationen skulle kunna bli bättre om kontinuitet i vården erbjöds. |
| Thirukumar et al. 2021 [32] | They reported that the medical staff were often unfamiliar with their situation and that that they had to repeat their story each visit. | Kvinnorna beskrev att personalen oftast inte kände till deras situation, därmed var de tvungna att upprepa deras berättelse vid varje sjukvårdskontakt. |
| Thirukumar et al. 2021 [32] | They suggested that if medical continuity of care was not possible, doctors' should "look at the file properly before seeing the patient:' | Om kontinuitet i vården inte var möjlig så föreslog kvinnorna att läkare skulle adekvat läsa journalanteckningarna inför mötet med patienter. |
| Weaver et al.  2007  [38] | It was clear from some of their comments about the reactions of health care professionals that the doctors and midwives caring for them did not always share their interpretation that their fear of vaginal birth was clinically justified. | Kvinnor upplevde att personalen inte alltid delade deras åsikt om att rädsla för vaginal förlossning var berättigat för att genomgå ett kejsarsnitt. |
| **Sub-theme 3: If women’s requests or wishes were denied or neglected, they developed strategies to obtain their CS anyways, through increased determination or repeating and defending their requests. Women with a previous CS sometimes became more open, sometimes more ambivalent toward CS in connection with their next pregnancy [25-28,30,35,37].** | | |
| **Author**  **Year**  **[Reference]** | **Meaning unit** | **Swedish translation** |
| Eide et al.  2019  [35] | Several women described being reluctant to become pregnant again after the last birth experience. | Omföderskor med önskemål om kejsarsnitt utan medicinsk indikation hade tvekat att bli gravida igen efter den tidigare förlossningsupplevelsen. |
| Eide et al.  2019  [35] | Several women described delaying a new pregnancy for many years. | Flera kvinnor med önskemål om kejsarsnitt utan medicinsk indikation hade senarelagt graviditet på grund av den tidigare förlossningsupplevelsen. |
| Eide et al.  2019  [35] | Several women described having received assurance of a planned CS prior to getting pregnant. | Flera kvinnor hade fått löfte om kejsarsnitt innan de planerade att bli gravida. |
| Emmett et al.  2006  [25] | Negotiation was typically with the hospital doctor, and women described either telling the doctor what they wanted at the outset or the doctor presenting the options and asking the woman what she wanted to do. | Kvinnor upplevde att de förhandlade med förlossningsläkaren, antingen berättade kvinnorna vad de önskade eller så presenterade förlossningsläkaren förlossningsalternativen och frågade vad kvinnan ville. |
| Fenwick et al.  2006  [26] | The analysis revealed that expectations about childbirth were reconstructed for the majority of women who had initially expected to have a vaginal birth but experienced a CS. | Majoriteten av kvinnorna uppgav att förväntningar på en vaginal förlossning hade omvärderats efter det första kejsarsnittet. |
| Fenwick et al.  2006  [26] | CS an acceptable option (after a previous CS). | Omföderskor med tidigare kejsarsnitt ansåg kejsarsnitt nu vara ett acceptabelt förlossningssätt. |
| Fenwick et al.  2006  [26] | Some women expressed uncertainty about subsequent birth options. | Några kvinnor uttryckte nu osäkerhet gällande kommande förlossningssätt. |
| Fenwick et al.  2006  [26] | Some women expressed uncertainty whether they would ever choose to fall pregnant again. | Några kvinnor uttryckte osäkerhet gällande om de någonsin skulle vilja bli gravida igen. |
| Fenwick et al.  2006  [26] | Women who had previously held firm views, for example, on birthing without intervention, now commented that such beliefs *had gone out the window*. | Omföderskor med tidigare kejsarsnitt uttryckte att deras tidigare syn på förlossning utan interventioner hade ändrats. |
| Fenwick et al.  2006  [26] | Women described being more open to, and understanding of, the need for CS. | Kvinnor uttryckte ökad förståelse och var mer öppna för kejsarsnitt. |
| Fenwick et al.  2006  [26] | There was also a sense in the data that for some women their previously held views on vaginal birth had been somewhat misplaced or incorrect. | Några kvinnor ansåg att deras åsikt om vaginal födsel hade varit inkorrekt. |
| Fenwick et al.  2006  [26] | Some of these women went as far as commenting that they would now recommend having a CS to their friends. | Några kvinnor med tidigare kejsarsnitt uttryckte att de nu skulle rekommendera kejsarsnitt till sina vänner. |
| Fenwick et al.  2006  [26] | The second identified shift in expectations reflects what women described as a more realistic approach to childbirth. | Kvinnor uttryckte att de nu hade en mer realistisk syn på barnafödande. |
| Fenwick et al.  2006  [26] | Women talked about the importance of now expecting the unexpected. | Omföderskor med tidigare kejsarsnitt talade om vikten att förvänta det oväntade. |
| Fenwick et al.  2006  [26] | Women talked about being more flexible in attitudes and beliefs. | Kvinnor med tidigare kejsarsnitt uttryckte att de var mer flexibla i attityder och föreställningar gällande barnafödande. |
| Fenwick et al.  2010  [27] | Some women disclosed non-clinical reasons that they perceived contributed to their doctor's acquiescence to their request for a caesarean section. | Några kvinnor ansåg att deras beskrivning av deras icke-medicinska orsaker bidrog till att läkare samtyckte till deras önskemål om kejsarsnitt. |
| Fenwick et al.  2010  [27] | During interactions with doctors, postscripts and/or after-thoughts (examples of non-medical reasons such as to be an older mother, bladder problems after vaginal birth) were frequently used to reinforce and validate the woman's decision to have a caesarean section. | Under möten med läkare användes icke-medicinska orsaker för att förstärka beslut om kejsarsnitt utan medicinsk indikation. |
| Fenwick et al.  2010  [27] | Denying a request for a caesarean section, would encourage women to find another doctor who would perform the procedure. | Avslag på kejsarsnittsönskan gjorde att kvinnor sökte annan läkare som accepterade kejsarsnitt. |
| Kenyon et al.  2016  [37] | Women felt they had to continually repeat and defend their decision to each different healthcare professional they saw and that *no one was listening*. | Kvinnorna upplevde att de behövde återkommande upprepa och försvara beslutet om kejsarsnitt inför personal som inte lyssnade. |
| Kenyon et al.  2016  [37] | Other themes that emerged from the discussion included […] the need for women to repeat reason for the caesarean section request to each health care professionals who cares for them. | Kvinnorna behövde upprepa önskemålet om kejsarsnitt till varje ny vårdpersonal de träffade. |
| Kenyon et al.  2016  [37] | During the antenatal care the women received, they found that they had to repeatedly come out as having requested a caesarean section and felt that they were required to defend that decision repeatedly. | De gravida kvinnorna upplevde att de var tvungna att upprepade gånger försvara deras beslut om kejsarsnitt. |
| Kenyon et al.  2016  [37] | If a woman was given an appointment with one of the BWNFT consultants who was not personally supportive of maternal request for caesarean section, the experience could be frustrating and distressing. The women often had to ask for *a second opinion*. | Vid samtal med personalen som inte gav stöd till deras beslut om kejsarsnitt upplevde kvinnorna frustration och oro, vilket ledde till en önskan om en *second opinion.* |
| Kornelsen et al.  2010  [28] | The consent process itself functioned as a way to gain *buy-in* from their care provider. | Förstföderskorna använde samtyckesprocessen som ett sätt för att *sälja in* sina önskemål om kejsarsnitt. |
| Kornelsen et al.  2010  [28] | The *buy-in*, in the consent process was required to secure a referral to the obstetrical care required for the procedure. | Det var ibland nödvändigt att *sälja in* sina önskemål o kejsarsnitt för att säkra en remiss till förlossningsvården. |
| Kornelsen et al.  2010  [28] | Many noted a willingness to pay for the procedure, despite the lack of a mechanism to allow for this. | Många kvinnor uppgav att de var villiga att betala för kejsarsnittet även om det inte fanns någon möjlighet till det. |
| Kornelsen et al.  2010  [28] | Several others expressed a willingness to leave the country to secure access to the procedure had that been necessary. | Flera kvinnor var beredda att lämna landet för att kunna få ett kejsarsnitt. |
| Kornelsen et al.  2010  [28] | Family physician’s reluctance led to participants invoking strategies (like the use of existing medical conditions) and, ultimately, adopting a sense of determination. | Familjeläkares motvillighet att agera utifrån önskemål om kejsarsnitt ledde till att kvinnorna blev påstridiga. |
| Ramvi et al.  2011  [30] | She received a date for the planned cesarean section and said that this promise was the reason why she was able to manage the pregnancy. | En omföderska med tidigare negativ förlossningsupplevelse och med önskan om ett planerat kejsarsnitt kunde hantera graviditeten genom att bli lovad ett datum för kejsarsnitt. |
