# Supplementary material for: Conflicting attitudes between clinicians and women regarding maternal requested caesarean section: a qualitative evidence synthesis
Source: BMC Pregnancy Childbirth. 2023 Mar 28;23:210. doi: 10.1186/s12884-023-05471-2 (PMC10044365; doi:10.1186/s12884-023-05471-2)
Supplement: Supplementary file 7 — Appendix VII. Data analysis process for clinicians’ perspectives [file 12884_2023_5471_MOESM7_ESM.docx]

| **Theme 1:**  **Clinicians were concerned about health risks associated with CS** | | |
| --- | --- | --- |
| ***Clinicians were concerned about the higher health risks associated with CS delivery compared to giving birth vaginally, and they were worried about the increasing prevalence of CS. In the decision-making process, they felt it was important to include considerations of health risks/consequences and to share these with the women.*** | | |
| **Sub-theme 1: Clinicians had concerns about the increased prevalence of non-medical CS, because it was regarded as involving greater risk of complications compared to a vaginal birth [33,34], and the increased number of CS reduced the capacity for treating other obstetrical and gynecological conditions [36].** | | |
| **Author,**  **Year [Reference]** | **Meaning unit** | **Swedish translation** |
| Eide et al., 2020 [36] | Some obstetricians saw it as […] especially in low-risk pregnancies where the evidence suggest a VD was undeniably the safest option for mother and child. | Några obstetriker ansåg att, speciellt vid lågriskgraviditeter, förespråkar evidensen otvetydigt en vaginal förlossning som det säkraste förlossningssättet för mamma och barn. |
| Eide et al., 2020 [36] | Obstetricians were concerned that a rise in CS rates would mean a reduction in surgery capacity for other gynecologic conditions. | Obstetrikerna var oroade för att den ökade kejsarsnittsfrekvensen kunde innebära en minskad kapacitet gällande kirurgi för andra gynekologiska tillstånd. |
| Kamal et al., 2005 [33] | Concerns around rising repeat caesarean rates were prominent in participants' accounts | Barnmorskor och läkare var oroade över ökningen av upprepade kejsarsnitt |
| Kamal et al., 2005 [33] | Most professionals suggested that vaginal birth was to be preferred because it poses fewer risks to mother and child and allows better maternal functioning in the weeks after birth | Barnmorskor och läkare ansåg att vaginal födsel innebar lägre risk för komplikationer och bidrar till snabbare återhämtning |
| Kamal et al., 2005 [33] | Most midwives and doctors felt that, notwithstanding they acknowledged benefits of vaginal birth | De flesta barnmorskor och läkare framhöll fördelarna med vaginal födsel |
| Kamal et al., 2005 [33] | The key distinction, in all accounts, between decision making for someone who had not had a previous caesarean and someone who had had a previous caesarean was the appropriate management of the presence of the scar from the previous section. Considerable concern was expressed about the risks posed by the scar during delivery | Barnmorskor och läkare uttryckte betydande oro för de risker ett ärr i livmodern efter kejsarsnitt medför under vaginal förlossning |
| Kamal 2005 [33] | Most midwives and doctors felt that, notwithstanding the acknowledged benefits of vaginal birth, there is a low threshold among obstetricians for performing caesarean, and that rates were too high. | De flesta deltagarna tyckte att bortsett från de bekräftade fördelarna med vaginal förlossning [VF], var tröskeln för låg bland förlossningsläkare att utföra KS, och att kejsarsnittsfrekvensen är för hög. |
| Karlström et al., 2009  [34] | The doctors were also concerned about the medical consequences of a high CS rate in terms of more complicated births in the future | Läkarna var oroade över medicinska konsekvenser av kejsarsnitt för mer komplicerade förlossningar i framtiden |
| Karlström 2009 [34] | Furthermore, the obstetricians noted that they were more likely now than before to accept women's request for CS rather than dealing with a "worst case scenario" if they promoted a vaginal birth. | Förlossningsläkarna noterade att de i större utsträckning nu än tidigare accepterade kvinnans önskemål hellre än att hantera ett scenario med sämsta möjliga utfall efter en VF som de själva hade förordat. |
| **Sub-theme 2: Clinicians were of the opinion that the consequences and risks of CS must be considered and must be communicated with women, without violating their integrity, during the decision-making process [34,36].** | | |
| **Author**  **Year**  **[Reference]** | **Meaning unit** | **Swedish translation** |
| Eide et al., 2020 [36] | Some obstetricians saw it as their main responsibility to inform the patient and help the patient make an informed choice about mode of delivery. | Några obstetriker ansåg att deras yttersta ansvar var att informera kvinnan som önskar kejsarsnitt utan medicinsk indikation och bistå med ett informerat val gällande förlossningssätt. |
| Karlström et al., 2009  [34] | The obstetricians described an obligation to clarify the medical risks with a surgical delivery. | Obstetriker ansåg att det var en skyldighet att förtydliga de medicinska riskerna med kejsarsnitt |
| Karlström et al., 2009  [34] | The obstetricians described an obligation to clarify the medical risks with a surgical delivery without violating her integrity. | Obstetriker ansåg att det var en skyldighet att förtydliga de medicinska riskerna med kejsarsnitt utan att kränka den gravida kvinnans integritet |
| Karlström et al., 2009  [34] | Participants also discussed the responsibility of both caregivers and expecting parents to consider the medical implications on an increasing CS rate | Barnmorskor och förlossningsläkare ansåg att det är ett ansvar för både personalen och blivande föräldrar att överväga medicinska konsekvenser av den ökade förekomsten av kejsarsnitt |
| Karlström et al., 2009  [34] | The professional starting point in discussions with women about mode of delivery should be that CS without medical indications is connected with more risks for both mother and child, today and in the future | Barnmorskor och förlossningsläkare ansåg det viktigt att förmedla att kejsarsnitt utan medicinsk indikation är förenat med risker för både mamma och barn |

| **Theme 2:**  **Demanding experience to consult women with a CS request** | | |
| --- | --- | --- |
| ***Women’s requests for CS were regarded by clinicians as based on misunderstandings about the advantages and disadvantages of a CS. Clinicians perceived it to be a demanding and time-consuming experience to handle a woman’s CS request. Clinicians felt that a high workload often led to complications during childbirth or negative birth experiences, that—when combined with deficient postnatal care—often led women to request delivery by CS in their next pregnancy.*** | | |
| **Sub-theme 1: Clinicians described that it was demanding to handle a woman’s request for a CS, and they could be resistant to or reluctant about the request. They believed it was a balancing act to direct women’s expectations with regard to the professionals’ and society’s expectations and the preference of a vaginal birth [34-38]. Clinicians regarded the consultations in which women requested CS as time-consuming and mentally exhausting [36]. Consultant midwives experienced their role as involving conflict when they had to persuade women to undergo a vaginal birth, instead of supporting their mode of birth preference [36].** | | |
| **Author**  **Year**  **[Reference]** | **Meaning unit** | **Swedish translation** |
| Eide et al., 2020 [36] | They […] did not see it worth their time and resources when it came to a patient dispute which was regarded time-consuming and mentally exhausting. | Obstetriker och barnmorskor ansåg att det är tidskrävande och mentalt utmattande att hamna i dispyt med kvinnor som önskar kejsarsnitt utan medicinsk indikation. |
| Eide et al., 2020 [36] | Several doctors had developed strategies to avoid making the situation more tense, by avoiding a negotiation table, *facilitating a shared decision-making process* and acknowledging mental health problems. | Flera av obstetrikerna hade utvecklat strategier för att undvika en spänd stämning vid konsultationen med kvinnor med önskemål om kejsarsnitt utan medicinsk indikation. Detta genom att förhandla, att gynna ett gemensamt beslut om förlossningssätt och att ta hänsyn till mental ohälsa. |
| Eide et al., 2020 [36] | There was a challenge of identifying which women were capable of coping with a new vaginal birth experience. | Obstetriker och barnmorskor ansåg det utmanande att identifiera vilka kvinnor som kunde hantera en upprepad vaginal förlossning. |
| Eide et al., 2020 [36] | Evaluating the woman’s mental health was regarded as highly subjective and difficult: | Obstetriker och barnmorskor ansåg det vara subjektivt och svårt att bedöma den mentala hälsan för de kvinnor som önskar kejsarsnitt utan medicinsk indikation. |
| Kenyon et al., 2016 [37] | The conflicting role of *Consultant Midwife* [that had her] persuading women to change their minds and try for a vaginal birth vs the need to support women in their choice. | Samtalsbarnmorskan ansågs ha en konfliktfylld roll i att övertyga kvinnor att ändra önskemål om kejsarsnitt till en vaginal förlossning istället för att stödja kvinnans förutbestämda önskemål |
| Weaver et al., 2007 [38] | Some obstetricians thought that many of these women did not truly want a caesarean section but saw this as the only way to guarantee the safety of the baby or to avoid a repeat of their previous frightening experience | Några obstetriker ansåg att kvinnorna egentligen inte önskade kejsarsnitt utan enbart ett sätt för att garantera säkerhet eller undvika upprepning av tidigare skrämmande upplevelse |
| Eide 2019 [35] | Willingness to comply with such requests [CS without medical reasons] was lower. | Förlossningsläkarna var mindre villiga att tillmötesgå sådana önskemål [om KS utan medicinsk orsak]. |
| Eide 2019 [35] | Willingness to spend time and effort on them [women who requested CS without medical reasons] varied. | Förlossningsläkarnas vilja att spendera tid och energi på dessa kvinnor [som ville ha KS utan medicinsk orsak] varierade. |
| Karlström 2009 [34] | Primiparaes requesting CS without medical reasons, however, caused strong reactions and met resistance. | Förstagångsföderskor som önskade ett KS utan medicinska grunder orsakade dock starka reaktioner och mötte motstånd. |
| Karlström 2009 [34] | However, the participants in the FGs viewed first-time mothers requesting a CS as provocative and difficult to understand. | Deltagarna såg dock förstföderskors önskemål om KS som provocerande och svårförståeliga. |
| Eide et al., 2020 [36] | Caregivers struggled between the responsibility for the individual woman and the responsibility towards the profession and society. | Obstetrikerna och barnmorskorna slets mellan ansvaret för den individuella kvinnan med önskemål om kejsarsnitt utan medicinsk indikation och gentemot professionen samt samhället. |
| Eide et al., 2020 [36] | Obstetricians balanced the responsibility towards the individual patient and the responsibility towards society during decision making. | I samband med beslutsprocessen för kvinnors önskemål om kejsarsnitt utan medicinsk indikation balanserade obstetrikerna ansvaret gentemot den individuella kvinnan och ansvaret gentemot samhället. |
| Eide et al., 2020 [36] | Caregivers found themselves pulled between the expectations of their profession and the intention to do good for the individual patient: | Obstetriker och barnmorskor drogs mellan förväntningarna från deras profession och deras intention att göra gott för den individuella kvinnan. |
| **Sub-theme 2: Clinicians found it to be difficult to inform women about the health risks of CS without frightening them [34,35]. Clinicians perceived women’s information as inconsistent [37] and not always based on scientific evidence [34,36,37]. Women’s requests for CS were believed to be based on misunderstandings about advantages and disadvantages of a CS [34-37].** | | |
| **Author**  **Year**  **[Reference]** | **Meaning unit** | **Swedish translation** |
| Karlström et al., 2009 [34] | The participants believed that the maternal request for CS was caused by women’s misunderstanding about the pros and cons of the operation. | Barnmorskor och förlossningsläkare ansåg att kvinnors önskan om kejsarsnitt var grundat i missförstånd om för- och nackdelar med kejsarsnitt |
| Karlström et al., 2009 [34] | It was considered difficult to inform women about risks as the participants did not want to frighten them. | Barnmorskor och förlossningsläkare upplevde svårigheter att ge information då de inte ville skrämma kvinnorna |
| Kenyon et al., 2016 [37] | The interviews identified variation in verbal information given to women (alongside the standard leaflets): It was agreed that information was inconsistent | Personalen ansåg att inkonsekvent information gavs till kvinnorna |
| Kenyon et al., 2016 [37] | It was agreed that the discussion sometimes lacked research information | Personalen ansåg att informationen som gavs inte alltid var evidensbaserad |
| Eide et al., 2019  [35] | Midwives acknowledged the difficult balance of providing enough information without creating unnecessary fear | Barnmorskor identifierade en svår balans mellan att ge tillräcklig information utan att skapa rädsla |
| Eide et al., 2019  [35] | Communication between caregivers was also described as giving rise to fear and misunderstanding. | Barnmorskor ansåg att [bristfällig] kommunikation mellan personal orsakade rädsla och missförstånd hos de gravida [gällande risker och fördelar med respektive förlossningssätt]. |
| Eide et al., 2020 [36] | Caregivers were concerned about how media and trends in society influenced women’s perception of need for CS. | Obstetriker och barnmorskor var oroade över hur media och sociala trender påverkade kvinnors uppfattning för behovet av att bli förlöst med kejsarsnitt. |
| Eide et al., 2020 [36] | several obstetricians mentioned a shift and a positive trend over the last few years after bloggers and celebrities had advocated for own vaginal birth experiences in media. | Flera obstetriker nämnde ett trendskifte och en positiv trend över de senaste åren där bloggare och kändisar förespråkade egna upplevelser av vaginal förlossning i media. |
| Eide et al., 2020 [36] | Many caregivers were concerned about the free access to unfiltered information on the internet, which was particularly unfortunate reading for women who were prone to anxiety. | Många av obstetrikerna och barnmorskorna var oroade över den fria tillgången till icke faktagranskad information på Internet. Denna information ansågs inverka negativt för kvinnor som har tendens till ångest. |
| **Sub-theme 3: Clinicians concluded that a heavy workload made it difficult to create a supportive and safe environment for women in labor [33,34,37], with the consequences of complicated childbirths [34,38], negative birth experiences [34], insufficient time for postnatal debriefing with women [33], and thus maternal requests for CS in subsequent pregnancies [33,34].** | | |
| **Author**  **Year**  **[Reference]** | **Meaning unit** | **Swedish translation** |
| Kenyon et al., 2016 [37] | This […] felt frustrating to health care professionals in terms of the extent to which they were able to provide good care | Barnmorskor och obstetriker upplevde det frustrerande att inte alltid få erbjuda god vård för kvinnor som önskar kejsarsnitt. |
| Karlström 2009 [34] | Working conditions were identified as a hindrance to provide high quality care | Arbetsförhållanden identifierades som ett hinder att ge högkvalitativ vård. |
| Karlström 2009 [34] | Obstetricians and midwives concluded that a heavy workload made it difficult to provide a supportive and safe environment. | Förlossningsläkare och barnmorskor konstaterade att en hög arbetsbelastning gjorde det svårt att erbjuda en stödjande och trygg miljö. |
| Karlström 2009 [34] | The participants also discussed the link between dissatisfaction with intrapartum care, negative birth experiences and a request for CS. | Deltagarna diskuterade också sambandet mellan otillfredsställelse med förlossningsvård, negativa förlossningsupplevelse och önskemål om KS. |
| Karlström 2009 [34] | There was a general opinion in the FGs that a strained situation in the delivery units had an impact on the birth outcome. | Den övergripande åsikten i fokusgrupperna var att den ansträngda situationen på förlossningsenheterna påverkade utfallet av förlossningarna. |
| Karlström 2009 [34] | When midwives had to be responsible for several women in active labour and doctors felt the stress of heavy workload, they found it difficult to provide high quality care. | När barnmorskor behövde vara ansvarig för flera födande kvinnor och läkarna upplevde stress pga hög arbetsbelastning, tyckte de det var svårt att erbjuda högkvalitativ vård. |
| Karlström 2009 [34] | Such stress in the intrapartum care was perceived to complicate births, leave the woman with a negative birth experience and be the cause of maternal request in a subsequent pregnancy. | En sådan stress i förlossningsvården uppfattades komplicera förlossningar, gav kvinnan en negativ förlossningsupplevelse och var orsaken till (KS) på moderns önskemål i samband med en kommande graviditet. |
| Weaver 2007 [38] | some doctors pointed out that finding the time to give to such women in their busy schedule could be extremely difficult | Vissa förlossningsläkare påpekade att det kunde vara mycket svårt att ge denna tid till dessa kvinnor på grund av deras pressade scheman. |
| Kamal 2005 [33] | Some obstetricians suggested that obstetricians did not have sufficient opportunity to discuss options with women who have had a previous section. | Vissa förlossningsläkare påpekade att de inte hade tillräckliga möjligheter att diskutera val med kvinnor som tidigare hade genomgått ett KS. |
| Kamal et al., 2005 [33] | Some participants also reported a view that women who do not receive adequate postnatal debriefing following their first caesarean are more likely to opt for a repeat caesarean. | Några barnmorskor och obstetriker rapporterade att otillräcklig *debriefing* efter det första kejsarsnittet ökar sannolikheten för att kvinnor väljer kejsarsnitt för nästa barns födelse. |
| Kamal et al., 2005 [33] | Some obstetricians suggested that obstetricians did not have sufficient opportunity to discuss options with women who have had a previous section | Några förlossningsläkare uppgav att det inte fanns tillräckligt många tillfällen att diskutera förlossningsalternativ med kvinnor som haft tidigare kejsarsnitt |
| Eide et al., 2019 [35] | The main challenge was to identify the women that needed such follow-up. | Obstetriker och barnmorskor ansåg det vara en utmaning att identifiera vilka kvinnor som behöver uppföljningssamtal efter förlossning. |

| **Theme 3:**  **Conflicting attitudes about women’s rights to choose a CS** | | |
| --- | --- | --- |
| ***Clinicians hold conflicting attitudes about women’s right to choose CS for their mode of birth. The clinicians had different beliefs about the definition of a medically indicated CS, including whether childbirth fear was a medical or a non-medical indication for CS. Clinicians felt it was important to have scientific evidence for the decision-making process. Health care organization and the capacity of the health care system were believed to influence a CS decision.*** | | |
| **Sub-theme 1: Clinicians hold conflicting attitudes about women’s right to choose CS for their mode of birth [33,34,36]. The clinicians felt that a woman has the right to choose CS if she is well informed [33,36] or that a woman has no right to choose CS [33].** | | |
| **Author**  **Year**  **[Reference]** | **Meaning unit** | **Swedish translation** |
| Eide et al., 2020 [36] | Obstetricians revealed different opinions on the appropriateness of CSMR | Obstetrikerna hade olika åsikter om lämpligheten till kejsarsnitt utifrån kvinnors eget önskemål. |
| Eide et al., 2020 [36] | different degree involved women in the actual decision. | Kvinnor med önskemål om kejsarsnitt utan medicinsk indikation involverades i olika omfattning i beslutet om förlossningssätt. |
| Eide et al., 2020 [36] | Most caregivers believed the medical responsibility of the final decision should be held by the obstetrician. | De flesta av obstetrikerna och barnmorskorna ansåg att det medicinska ansvaret för det slutgiltiga beslutet om förlossningssätt vid kvinnors önskemål om kejsarsnitt utan medicinsk indikation ska tas av obstetrikern. |
| Eide et al., 2020 [36] | Patient autonomy with regards to delivery mode was usually interpreted as a right to say no to treatment, but not the right to demand an intervention without a medical indication. | Kvinnans autonomi i relation till förlossningssätt ansågs oftast som kvinnans rättighet till att neka behandling och inte en rättighet att kräva en intervention utan medicinsk indikation. |
| Eide et al., 2020 [36] | Professional autonomy and the right to refuse to operate on a healthy woman was mentioned. | Professionell autonomi och rättighet att vägra utföra operationer på friska kvinnor nämndes av obstetriker och barnmorskor. |
| Eide et al., 2020 [36] | They regarded it as wrong to force a woman into a VD against her will | Obstetrikerna och barnmorskorna ansåg det fel att tvinga en kvinna att föda vaginalt mot hennes vilja. |
| Eide et al., 2020 [36] | Some caregivers usually let the women decide. This could provide trust and allow for a better dialogue. | Några av obstetrikerna och barnmorskorna lät kvinnor med önskemål om kejsarsnitt utan medicinsk indikation själva besluta om förlossningssätt. Detta förhållningssätt kunde förmedla tilltro och ge en bättre grund för dialog. |
| Eide et al., 2020 [36] | These caregivers believed that most women still chose a vaginal birth plan. | Obstetriker och barnmorskor ansåg att de flesta kvinnor föredrar en vaginal förlossning. |
| Eide et al., 2020 [36] | In some situations, caregivers regarded planned CS to be an appropriate option for the individual woman. | I visa situationer ansåg obstetrikerna och barnmorskorna att planerat kejsarsnitt är ett bra alternativ för kvinnor med önskemål om kejsarsnitt utan medicinsk indikation. |
| Eide et al., 2020 [36] | While some midwives thought fear of birth had become an increasing problem over past decades, | En del av barnmorskorna ansåg att förlossningsrädsla hade blivit ett större problem över de senaste årtiondena. |
| Eide et al., 2020 [36] | Obstetricians expressed varying practices when it came to declining requests. | Obstetrikerna beskrev olika förhållningssätt för att neka till kvinnors önskemål om kejsarsnitt utan medicinsk indikation. |
| Eide et al., 2020 [36] | Some obstetricians saw it as the right thing to do, or their duty, to decline a request if a woman came with a non-medical indication. | Några obstetriker ansåg att det var det rätta eller deras uppgift att neka kvinnor till kejsarsnitt utan medicinsk indikation. |
| Eide et al., 2020 [36] | Midwives highlighted that a forced delivery was a very bad starting point for a birth experience, which again could influence the attachment between mother and child. | Barnmorskor uppmärksammade att tvinga kvinnor som önskar kejsarsnitt utan medicinsk indikation till vaginal förlossning är en dålig start för deras förlossningsupplevelse. En negativ förlossningsupplevelse ansåg vidare kunna påverka anknytningen mellan mamman och barnet. |
| Eide et al., 2020 [36] | Some obstetricians saw it as their main responsibility to inform the patient and help the patient make an informed choice about mode of delivery. If she were able to make an informed choice, her choice should be respected. | Om kvinnan därefter, efter att hon har fått information, kan göra ett informerat val, ska hennes önskemål respekteras. |
| Eide et al., 2020 [36] | obstetricians expressed varying practices when it came to declining requests. | Obstetrikerna beskrev olika förhållningssätt för att neka till kvinnors önskemål om kejsarsnitt utan medicinsk indikation. |
| Kamal et al., 2005 [33] | Three distinct strategies for managing discussions with women could be identified from participants' accounts: consumerist; mutualistic; and paternalistic. | Barnmorskor och obstetriker beskrev tre distinkta strategier för samtal med kvinnor med tidigare kejsarsnitt om beslut av upprepat kejsarsnitt. Dessa strategier beskrevs som konsument-, överenskommelse- och paternalistisk strategi. |
| Kamal et al., 2005 [33] | The consumerist strategy involved giving information to women and encouraging them to make the decision. The choice must be fully informed, but the decision is always theirs. | Barnmorskor och obstetriker beskrev att konsumentstrategin uppmuntrade kvinnan att ta beslut om förlossningssätt efter att ha blivit fullt informerad. |
| Kamal et al., 2005 [33] | The consumerist approach allowed them to transfer the responsibility to the patient. | Barnmorskor och obstetriker beskrev att konsumentstrategin betydde att personalen överlät ansvaret för beslutet om förlossningssätt till kvinnan. |
| Kamal et al., 2005 [33] | The consumerist approach was attractive as a means of avoiding subsequent litigation where there was uncertainty of benefit. | Barnmorskor och obstetriker uppgav att konsumentstrategin innebar undvikande av åtal, när osäkerhet om fördelar med kejsarsnitt fanns i beslutet. |
| Kamal et al., 2005 [33] | The mutualistic strategy involved a more joint approach to decision making. | Barnmorskor och obstetriker förklarade att överenskommelsestrategin innebar en gemensam strategi i beslutsfattandet om förlossningssätt. |
| Kamal et al., 2005 [33] | With an apparently mutualistic negotiation, however, participants reported using strategies to guide women towards making professional preferred choices, for example emphasizing the risks associated with various options. | Överenskommelsestrategin innebar enligt barnmorskor och obstetriker att kvinnor styrdes till att ta det beslut som vårdgivarna föredrog, till exempel genom att beskriva risker med olika förslag. |
| Kamal et al., 2005 [33] | The paternalistic strategies involved highly directive styles. | Den paternalistiska beslutsstrategin för förlossningssätt innebar att i stor utsträckning ge direktiv. |
| Kamal et al., 2005 [33] | In the paternalistic strategies, women were not encouraged to see themselves as having choices. | I den paternalistiska strategin hade kvinnor inga valmöjligheter enligt barnmorskor och obstetriker. |
| Kamal et al., 2005 [33] | Nineteen participants proposed that women did have a right to choose the mode of delivery as long as they fully understood the issues. | 19 deltagare menade att kvinnor har rätt att välja förlossningssätt om de fullt förstår situationen. |
| Kamal et al., 2005 [33] | …..while two felt that women had an unconditional right to choose. | Två deltagare menade att kvinnor har ovillkorlig rätt att välja förlossningssätt. |
| Kamal et al., 2005 [33] | … two suggested that they (women) should have no rights. | Två deltagare menade att kvinnor inte har några rättigheter att välja förlossningssätt. |
| Kamal et al., 2005 [33] | Most midwives in the sample suggested that any woman who wanted to have a repeat caesarean should be able to do so as long as her decision has been adequately informed, locating their arguments for this within a discourse of 'choice': The pros and cons have to be clearly explained and then the woman yes she is allowed to choose. | De flesta barnmorskor ansåg att kvinnor som önskade ett upprepat kejsarsnitt skulle få det om de hade blivit välinformerade. Barnmorskornas argument för detta utkristalliserats inom diskursen val (rätten till val). För- och nackdelar måste förklaras tydligt och därefter ska kvinnan tillåtas välja. |
| Kamal et al., 2005 [33] | Contingency in this context refers to professionals' own history of managing women in labour. | Barnmorskors och obstetrikers tidigare upplevelser av förlossning påverkade beslutsfattandet om förlossningssätt. |
| Kamal et al., 2005 [33] | Participants emphasized that decisions about elective repeat caesarean section were not made solely by professionals but were instead joint decisions negotiated between women and professionals. | Barnmorskors och obstetrikers menade att beslut om upprepat kejsarsnitt togs gemensamt av kvinnan och personal. |
| Kamal et al., 2005 [33] | However, how professionals chose to manage consultations about these decisions varied widely. | Barnmorskors och obstetrikers menade att hur personalen hanterade konsultationer om beslut av förlossningssätt varierade stort. |
| Kamal et al., 2005 [33] | Different strategies were used depending on their level of agreement with what they perceived to be a woman's preference. | Barnmorskors och obstetrikers menade att olika beslutstrategier användes beroende på i vilken utsträckning de höll med om att kvinnans önskemål var det lämpliga förlossningssättet. |
| Kamal et al., 2005 [33] | Different strategies were used depending on how strong they perceived this preference to be. | Beslutstrategier baserades på grad av styrkan i kvinnors önskemål om förlossningssätt. |
| Karlström et al., 2009  [34] | Furthermore, the obstetricians noted that they …. accept[ed] women's request for CS rather than dealing with a "worst case scenario" if they promoted a vaginal birth. | Förlossningsläkarna noterade att de accepterade kvinnans önskemål hellre än att hantera ett scenario med sämsta möjliga utfall efter en VF som de själva hade förordat. |
| **Sub-theme 2: Clinicians felt it was important to have standardized definitions for medical and non-medical CS [33], but these are absent [33]. Some clinicians regarded childbirth fear as an indication for CS, while others did not [38]. Clinicians regarded it as important that the decision about mode of birth was made based on the individual woman’s unique circumstances rather than on a strict protocol [33].** | | |
| **Author**  **Year**  **[Reference]** | **Meaning unit** | **Swedish translation** |
| Kamal et al., 2005  [33] | The role of clinical indications in influencing whether a woman would have a repeat caesarean section was very prominent in participants' accounts. | Barnmorskor och läkare lyfte fram kliniska indikationer som viktigt för beslut om upprepat kejsarsnitt. |
| Kamal et al., 2005  [33] | Nevertheless, there was considerable variation as to which indications were identified as absolute indications, with the greatest consensus emerging in relation to fetal distress and breech presentations. | Det fanns betydande variation angående vad som identifierades som absoluta indikationer för kejsarsnitt där störst konsensus uppnåddes i relation till fetal distress och sätesändlägen. |
| Kamal et al., 2005  [33] | Accounts distinguished between indications for emergency and elective caesarean section. | Barnmorskor och läkare skilde mellan indikationer för planerade och akuta kejsarsnitt. |
| Kamal et al., 2005  [33] | Accounts distinguished between indications for indications that are identical to those for performing primary caesarean and those that apply only to women who have already had a caesarean. | Barnmorskor och läkare skilde mellan indikationer för primära och upprepade kejsarsnitt. |
| Kamal et al., 2005 [33] | Accounts identified two different categories of clinical indications for repeat caesarean section. One category involved lists of *absolute* clinical indications that were seen as necessitating a repeat caesarean. These indications were presented in participants' accounts as being inarguable: they were 'hard and fast'. The second category involved less absolute indications that might play a role in the decision to operate, including poor fetal growth and pre-eclampsia. | Barnmorskor och läkare identifierade två kategorier av kliniskt motiverade upprepade kejsarsnitt. En kategori involverade absoluta kliniska indikationer vilka ansågs nödvändiga för upprepat kejsarsnitt. Dessa absoluta indikationer för upprepade kejsarsnitt var odiskutabla och ansågs vara tydliga. Den andra kategorin involverade mindre absoluta indikationer vilka kunde ha betydelse för beslut att genomföra kejsarsnitt, vilka inkluderade tillväxthämning och preeklampsi. |
| Kamal et al., 2005 [33] | Participants indicated that if the same clinical indication that had prompted a previous caesarean arose in a subsequent birth, it would be logical to perform caesarean again. | Om samma kliniska indikation fortfarande fanns kvar som tidigare ansåg barnmorskor och läkare att det var logiskt med ett upprepat kejsarsnitt. |
| Kamal et al., 2005 [33] | Although a role for guidelines and protocols was recognised, caesarean section was identified in participants' accounts as an area that was especially ill-suited to being managed by strict protocols that would apply in each and every situation | Barnmorskor och läkare ansåg att strikta protokoll inte var lämpligt som underlag för beslut om kejsarsnitt utan att beslutet hellre ska vara individanpassat |
| Eide et al., 2020 [36] | Other obstetricians did not feel comfortable denying a woman a CS if she was completely reluctant towards giving birth, even in cases where fear was not prominent. | Andra obstetriker kände sig inte bekväma med att neka kvinnor att genomgå kejsarsnitt om kvinnan tydligt motsade sig en vaginal förlossning, även om inte förlossningsrädsla var uppenbart. |
| Eide et al., 2020 [36] | A previous traumatic birth experience and severe fear of childbirth were acknowledged as legitimate indications by several obstetricians: | En tidigare traumatisk förlossningsupplevelse och uttalad förlossningsrädsla ansågs av flertalet obstetriker som legitima skäl för kejsarsnitt utan medicinsk indikation. |
| Eide et al., 2020 [36] | Although planned CS could be advisable for women with a severe fear of childbirth, it was not regarded as a treatment for anxiety. | Obstetrikerna och barnmorskorna ansåg att planerat kejsarsnitt kunde vara lämpligt för kvinnor med uttalad förlossningsrädsla, men det ansågs inte vara en behandling för ångest. |
| Kamal et al., 2005 [33] | Others, however, argued that the decision should be made on 'clinical' grounds only. | Andra informanter (personal) menade att val av förlossningssätt enbart skulle göras på kliniska grunder. |
| Kamal 2005 [33] | There was considerable variation, for example, in the legitimacy given to 'psychological' reasons for requests for repeat caesarean section | Det fanns stora variationer i hur deltagarna såg på legitimiteten vad gäller "psykologiska" orsaker till önskemål om upprepat KS. |
| Kamal 2005 [33] | These accounts suggested that a repeat caesarean may be warranted for a woman who has no physical indications but is psychologically traumatised and dreads labour. | Dessa uttalanden föreslog att upprepat KS kan vara indicerat för en kvinna som inte har några fysiska indikationer (för KS) men är psykologiskt traumatiserad och som fruktar en förlossning. |
| Karlström 2009 [34] | A previous negative birth experience was respected and accepted as reason for elective CS. | En tidigare negativ förlossningsupplevelse respekterades och accepterades som orsak till elektivt KS. |
| Weaver 2007 [38] | Although doctors recognized women’s fears, some did not see these as constituting a clinical indication | Trots att förlossningsläkarna erkände kvinnornas rädslor, tyckte vissa av läkarna att rädslorna inte utgjorde en klinisk indikation. |
| **Sub-theme 3: Scientific evidence was described as an influential factor in the decision of mode of birth, but not the only factor in a CS decision. Clinicians argued whether available scientific evidence was of high methodological quality or not; sometimes, the evidence was difficult to assess and judge. Other influential factors in a CS decision were described as professional boundaries, content of care, structure of maternity care organization, and financial aspects [33,36,37].** | | |
| **Author**  **Year**  **[Reference]** | **Meaning unit** | **Swedish translation** |
| Kamal et al., 2005  [33] | Most participants identified 'evidence' as having an important role in relation to decision making for repeat caesarean, referring for example to systematic reviews, research, trials, studies and guidelines. | De flesta av barnmorskorna och läkarna ansåg att evidens spelade en viktig roll för beslut om upprepat kejsarsnitt med hänvisning till vetenskaplig litteratur och riktlinjer |
| Kamal et al., 2005  [33] | However, most accounts also emphasised that 'evidence' was not the sole basis for decision making | Barnmorskor och läkare ansåg också att enbart evidens var inte den enda grunden för beslut om förlossningssätt |
| Kamal et al., 2005  [33] | Concerns were expressed about the quality of evidence in this area | Läkare hyste oro för kvalitén på evidensen i området |
| Kamal et al., 2005  [33] | Midwives also described difficulties in accessing evidence or appraising it | Barnmorskor beskrev svårigheter med att få tag i och bedöma evidens |
| Kamal et al., 2005 [33] | It was clear from participants' accounts that organization of care was a key influence on repeat caesarean section. | Barnmorskor och obstetriker var tydliga med att vårdens organisation hade stor inverkan på andelen upprepade kejsarsnitt. |
| Kamal et al., 2005 [33] | Issues of professional boundaries were considered to be a key influence on repeat caesarean section. | Professionella gränser ansågs också påverka upprepade kejsarsnitt. |
| Kamal et al., 2005 [33] | In particular, how women were looked after antenatally was seen to be important. | Barnmorskor och obstetriker menade att vården under graviditeten hade betydelse för andelen kejsarsnitt. |
| Kamal et al., 2005 [33] | .. and by whom women were looked after antenatally was seen to be important. | Barnmorskor och obstetriker menade att vilken vårdgivare som vårdade den gravida kvinnan hade betydelse för andelen kejsarsnitt. |
| Kamal et al., 2005 [33] | Contingency in this context refers to the conditionality of action on features of the case, organisation of care, external contexts and aspects of the professional-patient relationship. | I detta sammanhang (beslutsprocessen om förlossningssätt) anses vidtagna åtgärder vara beroende på vårdens organisation, yttre faktorer samt relation mellan personal och patient. |
| Kamal et al., 2005 [33] | Participants' accounts described making decisions in the context of external pressures. For example, the conditions (e.g. availability of beds) of the unit in which professionals are working | Barnmorskor och obstetriker menade att yttre påverkande faktorer till exempel tillgängliga vårdplatser påverkade beslut om kejsarsnitt. |
| Kamal et al., 2005 [33] | Cost of caesarean section relative to vaginal deliveries all affected their decision making. | Kostnader för kejsarsnitt jämfört med vaginal födsel påverkade beslut om förlossningssätt. |
| Kenyon et al., 2016 [37] | The role of the Consultant Midwife was discussed and the part she played in the process explored. She was seen as the centre of the system as obstetricians refer onto her when a woman requested a caesarean section. | Personalen ansåg att samtalsbarnmorskan har en central roll i beslutsprocessen om förlossningssätt och är den profession obstetriker hänvisade till när kvinnor önskar kejsarsnitt. |
| Eide et al., 2020 [36] | The health budget for delivery clinics is performance based and paradoxically pays more for a CS than a VD. This was not regarded as an incentive among obstetricians for increasing CSMR. The clinics’ capacity for surgery was otherwise fixed. | Ekonomin för förlossningsvården var paradoxalt baserat på en högre ersättning för kejsarsnitt än för vaginal förlossning. Men detta var inte ansett som incitament för det ökade antalet kejsarsnitt utan medicinsk indikation. Förlossningsvårdens kapacitet för kirurgi var i övrigt fastlagt. |

| **Theme 4:**  **The importance of respectful and constructive dialogue about birthing options** | | |
| --- | --- | --- |
| ***The clinicians believed that support in the decision-making process involves engaging in respectful and constructive dialogue about birthing options. They considered it important to give women different kinds of support when consulting with them about a CS request. Support was preferably provided through evidence-based information, consultation in early pregnancy, and showing respect and understanding for the women’s request. Including a discussion about CS alternatives in the consultation was regarded as significant by the clinicians. Supporting women was regarded as enhancing women’s ability give birth vaginally, reducing women’s CS requests, and lower the prevalence of CS.*** | | |
| **Sub-theme 1: The clinicians found it important to support women before and during their pregnancy as well as postnatally [33,34,36-38]. It was important to offer women different kinds of support [34], such as evidence-based information [34] and consultations with women early in pregnancy. Professional support enhanced clinicians’ ability to give women the confidence to give birth vaginally [34].** | | |
| **Author**  **Year**  **[Reference]** | **Meaning unit** | **Swedish translation** |
| Eide et al., 2020 [36] | There was a prominent preference for VD as the outcome of counseling among all caregivers. | Bland alla obstetriker och barnmorskor så eftersträvades en önskan om vaginal förlossning som ett resultat av rådgivningen till kvinnor med önskemål om kejsarsnitt utan medicinsk indikation. |
| Karlström et al., 2009  [34] | the participants believed that various methods of support before, during and after birth would enhance and strengthen women’s abilities to give birth, and reduce the request. | Barnmorskor och förlossningsläkare ansåg att olika typer av stöd före, under och efter förlossning kan bidra till att stärka kvinnors förmåga att föda barn vaginalt, och kan bidra till att minska önskemålen om kejsarsnitt. |
| Karlström et al., 2009  [34] | The participants perceived birth preparation and antenatal classes as important tools in reducing the rate of CS. | Barnmorskor och förlossningsläkare ansåg att förberedelse och föräldrautbildning är viktiga redskap för att minska förekomsten av kejsarsnitt |
| Karlström et al., 2009  [34] | It was argued that giving birth is a natural process and a strong but painful experience that women must prepare for | Barnmorskor och förlossningsläkare argumenterade för att en vaginal förlossning är en naturlig smärtsam process som kräver förberedelse |
| Eide et al., 2019 [35] | Caregivers also emphasized the importance of a postpartum follow-up appointment after birth. This would be an opportunity for debriefing, answering questions and clearing up misunderstandings. | Obstetriker och barnmorskor betonade vikten av uppföljningssamtal med kvinnor som fött barn. Detta kunde ses som en möjlighet till *debriefing* samt ett tillfälle att svara på deras frågor och reda ut missförstånd. |
| Eide et al., 2020 [36] | Several caregivers pointed out the importance of primary care midwives in preparing women for their births. | Flera av obstetrikerna och barnmorskorna framhöll vikten av att mödrahälsovårdsbarnmorskor ska förbereda gravida kvinnor inför förlossning. |
| Eide et al., 2020 [36] | Midwives spent time exploring women’s fear and re-establishing safety and trust in order to help the woman find the best solution for herself. | Barnmorskor ägnade tid åt att utröna gravida kvinnors förlossningsrädsla och för att kvinnorna ska återfå trygghet och tillit, i syfte att bistå kvinnan till att finna den bästa lösningen för henne själv. |
| Eide et al., 2020 [36] | Through conversations, they guided the woman to find the right solution for her. | Genom samtal guidade barnmorskor kvinnor att finna den rätta lösningen för dem. |
| Karlström et al., 2009 [34] | There was a strong support in the FGs for special consultations for women who experience fear and anxiety when facing birth. | Barnmorskor och förlossningsläkare förordade att erbjuda stödsamtal för kvinnor med rädsla och oro inför förlossningen. |
| Eide et al., 2020 [36] | They spent time making a birth plan, which was a document providing safety for the woman. | Barnmorskorna tog sig tid till att upprätta en förlossningsplan. Planen ingav den gravida kvinnan en trygghet inför födandet. |
| Eide et al., 2020 [36] | Their goal was to follow the women, guide them through a thought process and deliver them as confident as possible to the delivery situation, irrespective of mode. | Barnmorskornas mål var att bistå kvinnor, guida dem genom beslutsprocessen och bistå dem med trygghet under deras förlossning, oavsett förlossningssätt |
| Kamal et al., 2005 [33] | Their accounts recognise that achieving the trust of the mother requires spending time talking to her, a role that was seen as being particularly suitable for midwives. | Barnmorskor och obstetriker ansåg det viktigt att tillbringa tid med kvinnan för att erhålla hennes förtroende, där barnmorskor ansågs särskilt lämpade. |
| Kamal et al., 2005 [33] | Participants reported that it is easier to convince women who trust their professionals completely to try for a vaginal delivery | Barnmorskor och läkare ansåg att det var lättare att övertyga kvinnor som litade på vårdgivarna, att prova vaginal födsel |
| Karlström et al., 2009 [34] | The midwife’s role was highlighted [decision making]. | Barnmorskans roll för stöd i beslutsprocessen om förlossningssätt ansågs viktig av personalen. |
| Weaver et al., 2007 [38] | Several obstetricians discussed the significance of women’s fears and the importance of taking the time to talk to women about these fears | Flera obstetriker ansåg det viktigt att ta sig tid till att prata med kvinnor om deras förlossningsrädsla |
| Weaver et al., 2007 [38] | Many of the doctors stressed the importance of taking time with women to find out what was behind the request | Många läkare ansåg det viktigt att ta sig tid med kvinnor för att förstå vad som ligger till grund för önskemålet om kejsarsnitt |
| Karlström et al., 2009  [34] | It was considered important to give evidence-based knowledge about vaginal birth and CS | Barnmorskor och förlossningsläkare ansåg det viktigt att ge evidensbaserad information om olika förlossningssätt |
| Kenyon et al., 2016  [37] | It was agreed that the discussion should include quality of information | Personalen ansåg att samtalet med kvinnan ska innefatta evidensbaserad information |
| Karlström et al., 2009  [34] | Participants believed that information at an early stage and to a sufficient degree could change women’s preferences towards vaginal birth | Barnmorskor och förlossningsläkare ansåg att information i tidigt skede och i tillräcklig omfattning kunde medföra att kvinnor ändrar önskemålet om kejsarsnitt till vaginal förlossning |
| Eide et al., 2020 [36] | They called for better access to and earlier appointments with midwives in pregnancy. | Obstetriker och barnmorskor efterfrågade för kvinnor bättre tillgång och större möjlighet till tidigare möte med barnmorskor under deras graviditet. |
| Eide et al., 2020 [36] | Midwives believed that early exploration of thoughts about birth could help pregnant women normalize fear and avoid medicalization. | Barnmorskor framhöll vikten av att tidigt under graviditet utröna kvinnors tankar om barnafödandet, vilket skulle kunna bidra till att normalisera kvinnors förlossningsrädsla och till att undvika medikalisering av födandet. |
| Eide et al., 2020 [36] | Caregivers emphasized the importance of getting into dialogue with women early on in pregnancy and giving the process time to mature and follow its course. | Obstetriker och barnmorskor framhöll vikten av att ha en dialog med kvinnor i tidig graviditet för att kunna få tillräckligt med tid för att processa deras önskemål om kejsarsnitt utan medicinsk indikation. |
| Kenyon et al., 2016 [37] | The health care professionals also spoke about the importance of timing in relation to when to talk to women about their choices and options | Barnmorskor och obstetriker ansåg att tidpunkten för samtal om förlossningssätt med kvinnor om deras val och alternativ är viktigt |
| Kenyon et al., 2016 [37] | Inconsistency of timing of information, when discussion is begun in late pregnancy, the rush to make suitable preparation for birth and the need for early discussion and so [delayed timing] time to plan for appropriate referrals where necessary and for birth | Barnmorskor och obstetriker ansåg tidpunkten för information till kvinnor var viktig. Att diskutera med kvinnor i sen graviditet leder till brådska med förlossningsplanering. Det är viktigt med tidig diskussion under graviditet så att tid finns för lämplig remittering för förlossningsplanering om behov så finns |
| **Sub-theme 2: It was important to provide women with enough consulting time [38], respect, and understanding [34,36,38]. Promoting a constructive dialogue [36] and including discussion of a vaginal birth alternative were also important in the consulting process [34,36,38]. Professional support enhanced clinicians’ ability to strengthen women’s confidence in giving birth vaginally [34], decreased women’s CS requests [34], and lowered the prevalence of CS [34].** | | |
| **Author**  **Year**  **[Reference]** | **Meaning unit** | **Swedish translation** |
| Eide et al., 2020 [36] | Achieving a good dialogue was important. | Barnmorskorna ansåg det viktigt att uppnå en god dialog med den gravida kvinnan som önskar kejsarsnitt utan medicinsk indikation. |
| Eide et al., 2020 [36] | Both midwives and obstetricians highlighted the advantage of midwives, without mandate to make the final decision, to promote a constructive dialogue. | Både barnmorskor och obstetriker lyfte fram fördelen med att barnmorskor inte ansvarar för det slutgiltiga beslutet för förlossningssätt, och vikten av att främja en konstruktiv dialog med den gravida kvinnan som önskar kejsarsnitt utan medicinsk indikation. |
| Karlström et al., 2009 [34] | The respondents stressed the importance of having a dialogue with the woman. | Barnmorskor och förlossningsläkare ansåg det viktigt att ha en dialog med kvinnor som önskar kejsarsnitt utan medicinsk indikation. |
| Eide et al., 2020 [36] | Midwives working with counselling described how they invested time and effort in establishing a good dialogue with women. | Barnmorskor som arbetade med samtalsstöd för gravida kvinnor beskrev hur de investerade i tid och kraft för att etablera en bra dialog med kvinnorna. |
| Karlström et al., 2009 [34] | The woman with tocophobia should be met with respect and understanding. | Barnmorskor och förlossningsläkare ansåg att kvinnor med förlossningsrädsla ska mötas med respekt och förståelse i beslutsprocessen om förlossningssätt. |
| Karlström et al., 2009 [34] | Midwives and obstetricians emphasized that to be professional implied commitment and authority without getting into conflicts with the patients. | Barnmorskor och obstetriker ansåg att vara professionell inbegriper engagemang och auktoritet utan att komma i konflikt med patienterna. |
| Karlström et al., 2009 [34] | The question of autonomy, when it came to mode of delivery, was most frequently discussed in the FGs where obstetricians took part. | Obstetriker beskrev att det handlar om att respektera kvinnans autonomi i förhållande till förlossningssätt. |
| Eide et al., 2020 [36] | Showing respect and taking women seriously often helped them re-establish trust, which had commonly been lost in an earlier birth experience | Genom att barnmorskorna visar respekt och genom att ha seriösa samtal med kvinnor hjälper detta dem till att återfå tillit, vilket vanligtvis hade gått förlorat i samband med tidigare förlossning. |
| Karlström et al., 2009  [34] | Requests for CS should be respected but alternatives must always be discussed | Barnmorskor och förlossningsläkare ansåg att kvinnors önskemål om kejsarsnitt ska respekteras men alternativ måste alltid diskuteras |
| Weaver et al., 2007  [38] | Many of the doctors stressed the importance of taking time with women to find out what was behind the request, and where appropriate, exploring safe and acceptable alternatives | Många läkare ansåg det viktigt att ta sig tid för att utröna orsaken till önskemålet, och när lämpligt  utröna säkra och acceptabla alternativ |
