# Supplementary material for: Conflicting attitudes between clinicians and women regarding maternal requested caesarean section: a qualitative evidence synthesis
Source: BMC Pregnancy Childbirth. 2023 Mar 28;23:210. doi: 10.1186/s12884-023-05471-2 (PMC10044365; doi:10.1186/s12884-023-05471-2)
Supplement: Supplementary file 8 — Appendix VIII. Excluded studies [file 12884_2023_5471_MOESM8_ESM.docx]

| **Excluded studies** | **Reason for exclusion** |
| --- | --- |
| Abbaspoor Z, Moghaddam-Banaem L, Ahmadi F, Kazemnejad A. Iranian mothers' selection of a birth method in the context of perceived norms: a content analysis study. Midwifery, 2014; 30 (7): 804-9. | Wrong context |
| Akgun M, Boz I. Women's decision-making processes and experiences of vaginal birth after caesarean birth: A phenomenological study. International Journal of Nursing Practice, 2019; 25 (6): e12780. | Wrong phenomena |
| Akhter S, Schech S. Choosing caesareans? The perceptions and experiences of childbirth among mothers from higher socio-economic households in Dhaka. Health Care for Women International, 2018; 39 (11): 1177-92. | Wrong context |
| Arthur D, Payne D. Maternal request for an elective caesarean section. New Zealand College of Midwives Journal, 2005; (33): 17-20. | Wrong publication type |
| Bagheri A, Masoudi Alavi N, Abbaszadeh F. Iranian obstetricians' views about the factors that influence pregnant women's choice of delivery method: a qualitative study. Women & Birth: Journal of the Australian College of Midwives, 2013; 26 (1): e45-9. | Wrong phenomena |
| Begum T, Ellis C, Sarker M, Rostoker JF, Rahman A, Anwar I, et al. A qualitative study to explore the attitudes of women and obstetricians towards caesarean delivery in rural Bangladesh. BMC Pregnancy & Childbirth, 2018; 18 (1): 368. | Wrong phenomena |
| Bluml V, Stammler-Safar M, Reitinger AK, Resch I, Naderer A, Leithner K. A qualitative approach to examine women's experience of planned cesarean. JOGNN - Journal of Obstetric, Gynecologic, & Neonatal Nursing, 2012; 41 (6): E82-90. | Wrong study design |
| Boucherie A-S, Girault A, Berlingo L, Goffinet F, Ray CL. Cesarean delivery on maternal request: How do French obstetricians feel about it? | Wrong study design |
| Boz I, Teskereci G, Akman G. How did you choose a mode of birth? Experiences of nulliparous women from Turkey. Women & Birth: Journal of the Australian College of Midwives, 2016; 29 (4): 359-67. | Wrong phenomena |
| Bringedal H, Aune I. Able to choose? Women's thoughts and experiences regarding informed choices during birth. Midwifery, 2019; 77123-29. | Wrong phenomena |
| Campo-Engelstein L, Howland LE, Parker WM, Burcher P. Scheduling the Stork: Media Portrayals of Women's and Physicians' Reasons for Elective Cesarean Delivery. Birth, 2015; 42 (2): 181-8. | Wrong study design |
| Chadwick RJ, Foster D. Technologies of gender and childbirth choices: Home birth, elective caesarean and white femininities in South Africa. Feminism and Psychology, 2013; 23 (3): 317-38. | Wrong phenomena |
| Chan MH, Hauck Y, Kuliukas L, Lewis L. Women's experiences of their involvement around care decisions during a subsequent pregnancy after a previous caesarean birth in Western Australia. Women & Birth: Journal of the Australian College of Midwives, 2020; 2828. | Wrong population - not CSMR |
| Chen SW, Hutchinson AM, Nagle C, Bucknall TK. Women's decision-making processes and the influences on their mode of birth following a previous caesarean section in Taiwan: a qualitative study. BMC Pregnancy & Childbirth, 2018; 18 (1): 31. | Wrong context |
| Cheung NF, Mander R, Cheng L, Chen VY, Yang X. Caesarean decision-making: negotiation between Chinese women and healthcare professionals. Evidence Based Midwifery, 2006; 4 (1): 24-30. | Wrong context |
| Chigbu CO, Ezeome IV, Iloabachie GC. Cesarean section on request in a developing country. International Journal of Gynaecology & Obstetrics, 2007; 96 (1): 54-6. | Wrong study design |
| Chinkam S, Mezwa K, Pierre KA, Steer-Massaro C, Herbey I, Louis KR, Mars D, Shorten A. Listening to Haitian women and their health care providers: insight into shared | Wrong population |
| Cindoglu D, Sayan-Cengiz F. Medicalization discourse and modernity: Contested meanings over childbirth in contemporary Turkey. Health Care for Women International, 2010; 31 (3): 221-43. | Wrong phenomena |
| Colomar M, Cafferata ML, Aleman A, Castellano G, Elorrio EG, Althabe F, et al. Mode of childbirth in low-risk pregnancies: Nicaraguan physicians' viewpoints. Maternal & Child Health Journal, 2014; 18 (10): 2382-92. | Wrong phenomena |
| Copelli FHS, Rocha L, Zampieri MFM, Gregório VRP, Custódio ZAO. Determinants of women’s preference for cesarean section. Texto e Contexto Enfermagem, 2015; 24 (2): 336-43. | Wrong phenomena |
| Dahlen HG, Homer CS. 'Motherbirth or childbirth'? A prospective analysis of vaginal birth after caesarean blogs. Midwifery, 2013; 29 (2): 167-73. | Wrong phenomena |
| David S, Fenwick J, Bayes S, Martin T. A qualitative analysis of the content of telephone calls made by women to a dedicated 'Next Birth After Caesarean' antenatal clinic. Women & Birth: Journal of the Australian College of Midwives, 2010; 23 (4): 166-71. | Wrong phenomena |
| Douche J, Carryer J. Caesarean section in the absence of need: a pathologising paradox for public health? Nursing Inquiry, 2011; 18 (2): 143-53. | Wrong phenomena |
| Edmonds JK, Jones EJ. Intrapartum nurses' perceived influence on delivery mode decisions and outcomes. JOGNN - Journal of Obstetric, Gynecologic, & Neonatal Nursing, 2013; 42 (1): 3-11. | Wrong phenomena |
| Faisal I, Matinnia N, Hejar AR, Khodakarami Z. Why do primigravidae request caesarean section in a normal pregnancy? A qualitative study in Iran. Midwifery, 2014; 30 (2): 227-33. | Wrong context |
| Feely C, Down S, Thomson G.'Stories of distress versus fulfilment': A narrative inquiry of midwives' experiences supporting alternative birth choices in the UK National Health Service. | Wrong population |
| Fleming V, Meyer Y, Frank F, van Gogh S, Schirinzi L, Michoud B, et al. Giving birth: Expectations of first time mothers in Switzerland at the mid point of pregnancy. Women & Birth: Journal of the Australian College of Midwives, 2017; 30 (6): 443-49. | Wrong phenomena |
| Galvao R, Hawley NL, da Silva CS, Silveira MF. How obstetricians and pregnant women decide mode of birth in light of a recent regulation in Brazil. Women & Birth: Journal of the Australian College of Midwives, 2018; 31 (5): e310-e17. | Wrong population |
| Goodall KE, McVittie C, Magill M. Birth choice following primary Caesarean section: mothers' perceptions of the influence of health professionals on decision-making. Journal of Reproductive & Infant Psychology, 2009; 27 (1): 4-14. | Wrong phenomena |
| Greer J, Lazenbatt A, Dunne L. ‘Fear of childbirth’ and ways of coping for pregnant women and their partners during the birthing process: a salutogenic analysis. Evidence Based Midwifery, 2014; 12 (3): 95-100. | Wrong phenomena |
| Gu C, Zhu X, Ding Y, Setterberg S, Wang X, Tao H, et al. A qualitative study of nulliparous women's decision making on mode of delivery under China's two-child policy. Midwifery, 2018; 626-13. | Wrong phenomena |
| Guittier M-J, Bonnet J, Jarabo G, Boulvain M, Irion O, Hudelson P. Breech presentation and choice of mode of childbirth: A qualitative study of women’s experiences. Midwifery, 2011; 27 (6): e208-13. | Wrong phenomena |
| Guzikowski W, Motak-Pochrzêst H, Kudaś D. Opinions of parturient women on the possibility of choice of the cesarean section without medical indications - Only at own request. Ginekologia i Poloznictwo, 2010; 17 (3): 53-58. | Wrong language |
| Halvorsen L, Nerum H, Oian P, Sorlie T. Giving birth with rape in one's past: a qualitative study. Birth, 2013; 40 (3): 182-91. | Wrong phenomena |
| Hatamleh R, Abujilban S, Al-Shraideh AJ, Abuhammad S. Maternal request for cesarian birth without medical indication in a group of healthy women: A qualitative study in Jordan. Midwifery, 2019; 79102543. | Wrong phenomena |
| Hofberg K, Brockington I. Tokophobia: an unreasoning dread of childbirth. A series of 26 cases. British Journal of Psychiatry, 2000; 17683-5. | Wrong study design |
| Huang SY, Sheu SJ, Tai CJ, Chiang CP, Chien LY. Decision-making process for choosing an elective cesarean delivery among primiparas in Taiwan. Maternal & Child Health Journal, 2013; 17 (5): 842-51. | Wrong context |
| Kabakian-Khasholian T. 'My pain was stronger than my happiness': experiences of caesarean births from Lebanon. Midwifery, 2013; 29 (11): 1251-6. | Wrong context |
| Karlstrom A, Nystedt A, Johansson M, Hildingsson I. Behind the myth--few women prefer caesarean section in the absence of medical or obstetrical factors. Midwifery, 2011; 27 (5): 620-7. | Wrong phenomena |
| Kennedy HP, Grant J, Walton C, Sandall J. Elective caesarean delivery: a mixed method qualitative investigation. Midwifery, 2013; 29 (12): e138-44. | Serious methodological flaws |
| Kingdon C, Neilson J, Singleton V, Gyte G, Hart A, Gabbay M, et al. Choice and birth method: mixed-method study of caesarean delivery for maternal request. BJOG: An International Journal of Obstetrics & Gynaecology, 2009; 116 (7): 886-95. | Wrong phenomena |
| Kurtoglu E, Arpaci H, Temur M. Family physicians' views on caesarean delivery on maternal request. Journal of Clinical and Analytical Medicine, 2013; 4 (1): 44-47. | Wrong study design |
| Kurtz Landy C, Sword W, Kathnelson JC, McDonald S, Biringer A, Heaman M, et al. Factors obstetricians, family physicians and midwives consider when counselling women about a trial of labour after caesarean and planned repeat caesarean: a qualitative descriptive study. BMC Pregnancy & Childbirth, 2020; 20 (1): 367. | Wrong population - not CSMR |
| Latifnejad-Roudsari R, Zakerihamidi M, Merghati-Khoei E, Kazemnejad A. Cultural perceptions and preferences of Iranian women regarding cesarean delivery. Iranian Journal of Nursing and Midwifery Research, 2014; 19 (7): S28-36. | Wrong phenomena |
| Lavender T, Kingdon C. Primigravid women's views of being approached to participate in a hypothetical term cephalic trial of planned vaginal birth versus planned cesarean birth. Birth, 2009; 36 (3): 213-9. | Wrong phenomena |
| Lee LY, Holroyd E, Ng CY. Exploring factors influencing Chinese women's decision to have elective caesarean surgery. Midwifery, 2001; 17 (4): 314-22. | Wrong phenomena |
| Lewis L, Hauck YL, Ritchie S, Barnett L, Nunan H, Rivers C. Australian women's perception of their preparation for and actual experience of a recent scheduled caesarean birth. Midwifery, 2014; 30 (3): e131-6. | Wrong phenomena |
| Litorp H, Mgaya A, Kidanto HL, Johnsdotter S, Essen B. 'What about the mother?' Women's and caregivers' perspectives on caesarean birth in a low-resource setting with rising caesarean section rates. Midwifery, 2015; 31 (7): 713-20. | Wrong phenomena |
| Liu NH, Mazzoni A, Zamberlin N, Colomar M, Chang OH, Arnaud L, et al. Preferences for mode of delivery in nulliparous Argentinean women: a qualitative study. Reproductive Health, 2013; 10 (1): 2. | Wrong phenomena |
| Malacrida C, Boulton T. The best laid plans? Women's choices, expectations and experiences in childbirth. Health: an Interdisciplinary Journal for the Social Study of Health, Illness & Medicine, 2014; 18 (1): 41-59. | Wrong phenomena |
| Manesh MJ, Jouybari L, Fatemeh Oskouie S, Sanagoo A. How do women's decisions process to elective cesarean?: A qualitative study. Australian Journal of Basic and Applied Sciences, 2011; 5 (6): 210-15. | Wrong phenomena |
| McGrath P, Ray-Barruel G. The easy option? Australian findings on mothers' perception of elective Caesarean as a birth choice after a prior Caesarean section. International Journal of Nursing Practice, 2009; 15 (4): 271-9. | Wrong phenomena |
| Moffat MA, Bell JS, Porter MA, Lawton S, Hundley V, Danielian P, et al. Decision making about mode of delivery among pregnant women who have previously had a caesarean section: A qualitative study. BJOG: An International Journal of Obstetrics & Gynaecology, 2007; 114 (1): 86-93. | Wrong population |
| Munro S, Janssen P, Corbett K, Wilcox E, Bansback N, Kornelsen J. Seeking control in the midst of uncertainty: Women's experiences of choosing mode of birth after caesarean. Women & Birth: Journal of the Australian College of Midwives, 2017; 30 (2): 129-36. | Wrong phenomena |
| Munro S, Kornelsen J, Corbett K, Wilcox E, Bansback N, Janssen P. Do Women Have a Choice? Care Providers' and Decision Makers' Perspectives on Barriers to Access of Health Services for Birth after a Previous Cesarean. Birth, 2017; 44 (2): 153-60. | Wrong phenomena |
| Munro S, Kornelsen J, Hutton E. Decision making in patient-initiated elective cesarean delivery: the influence of birth stories. Journal of Midwifery & Women's Health, 2009; 54 (5): 373-79. | Wrong phenomena |
| Murray-Davis B, McVittie J, Barrett JF, Hutton EK, Twin Birth Study Collaborative G. Exploring Women's Preferences for the Mode of Delivery in Twin Gestations: Results of the Twin Birth Study. Birth, 2016; 43 (4): 285-92. | Wrong phenomena |
| Panda S, Daly D, Begley C, Karlstrom A, Larsson B, Back L, et al. Factors influencing decision-making for caesarean section in Sweden - a qualitative study. BMC Pregnancy & Childbirth, 2018; 18 (1): 377. | Serious methodological flaws |
| Penna L, Arulkumaran S. Cesarean section for non-medical reasons. International Journal of Gynaecology & Obstetrics, 2003; 82 (3): 399-409. | Wrong study design |
| Petrovska K, Watts N, Sheehan A, Bisits A, Homer C. How do social discourses of risk impact on women’s choices for vaginal breech birth? A qualitative study of women’s experiences. Health, Risk & Society, 2017; 19 (1): 19-37. | Wrong phenomena |
| Puia D. A Meta-Synthesis of WOMEN'S Experiences of CESAREAN BIRTH. MCN: The American Journal of Maternal Child Nursing, 2013; 38 (1): 41-47. | Wrong study design |
| Quiroz LH, Blomquist JL, Macmillan D, McCullough A, Handa VL. Maternal goals for childbirth associated with planned vaginal and planned cesarean birth. American Journal of Perinatology, 2011; 28 (9): 695-702. | Wrong study design |
| Rees KM, Shaw AR, Bennert K, Emmett CL, Montgomery AA. Healthcare professionals' views on two computer-based decision aids for women choosing mode of delivery after previous caesarean section: a qualitative study. BJOG: An International Journal of Obstetrics & Gynaecology, 2009; 116 (7): 906-14. | Wrong phenomena |
| Regan M, McElroy KG, Moore K. Choice? Factors That Influence Women's Decision Making for Childbirth. Journal of Perinatal Education, 2013; 22 (3): 171-80. | Wrong phenomena |
| Rietveld AL, de Groot CJM, Teunissen PW. Decision-making during trial of labour after caesarean; a qualitative study with gynaecologists. PLoS ONE [Electronic Resource], 2018; 13 (7): e0199887. | Wrong phenomena |
| Rietveld AL, van Exel NJA, Cohen de Lara MC, de Groot CJM, Teunissen PW. Giving birth after caesarean: Identifying shared preferences among pregnant women using Q methodology. Women & Birth: Journal of the Australian College of Midwives, 2020; 33 (3): 273-79. | Wrong study design |
| Sanders RA, Crozier K. How do informal information sources influence women's decision-making for birth? A meta-synthesis of qualitative studies. BMC Pregnancy and Childbirth, 2018; 18 (1). | Wrong study design |
| Sapountzi-Krepia D, Tsaloglidou A, Psychogiou M, Lazaridou C, Vehvilainen Julkunen K. Mothers' experiences of pregnancy, labour and childbirth: A qualitative study in Northern Greece. International Journal of Nursing Practice (John Wiley & Sons, Inc.), 2011; 17 (6): 583-90. | Wrong phenomena |
| Schantz C, Sim KL, Petit V, Rany H, Goyet S. Factors associated with caesarean sections in Phnom Penh, Cambodia. Reproductive Health Matters, 2016; 24 (48): 111-21. | Wrong phenomena |
| Shahoei R, Riji HM, Saeedi ZA. 'Safe passage': pregnant Iranian Kurdish women's choice of childbirth method. Journal of Advanced Nursing (John Wiley & Sons, Inc.), 2011; 67 (10): 2130-38. | Wrong phenomena |
| Shahoei R, Rostami F, Khosravi F, Ranayi F, Hasheminasab L, Hesami K, et al. Women lived experience of choice of cesarean delivery: A phenomenology study. Iranian Journal of Obstetrics, Gynecology and Infertility, 2014; 17 (104): 1-10. | Wrong language |
| Shahraki Sanavi F, Rakhshani F, Ansari-Moghaddam A, Edalatian M. Reasons for Elective Cesarean Section amongst Pregnant Women; A Qualitative Study. Journal of Reproduction & Infertility, 2012; 13 (4): 237-40. | Wrong phenomena |
| Shorten A, Shorten B, Kennedy HP. Complexities of choice after prior cesarean: a narrative analysis. Birth, 2014; 41 (2): 178-84. | Wrong phenomena |
| Silva GPS, de Jesus MCP, Merighi MAB, Domingos SRdF, Oliveira DMd. The experience of women regarding cesarean section from the perspective of social phenomenology. Online Brazilian Journal of Nursing, 2014; 13 (1): 5-14. | Wrong publication type |
| Tadevosyan M, Ghazaryan A, Harutyunyan A, Petrosyan V, Atherly A, Hekimian K. Factors contributing to rapidly increasing rates of cesarean section in Armenia: a partially mixed concurrent quantitative-qualitative equal status study. BMC Pregnancy & Childbirth, 2019; 19 (1): N.PAG-N.PAG. | Wrong context |
| Takegata M, Haruna M, Morikawa M, Yonezawa K, Komada M, Severinsson E. Qualitative exploration of fear of childbirth and preferences for mode of birth among Japanese primiparas. Nursing & Health Sciences, 2018; 20 (3): 338-45. | Wrong phenomena |
| Takegata M, Smith C, Nguyen HAT, Thi HH, Thi Minh TN, Day LT, et al. Reasons for Increased Caesarean Section Rate in Vietnam: A Qualitative Study among Vietnamese Mothers and Health Care Professionals. Healthcare, 2020; 8 (1): 21. | Wrong context |
| Tully KP, Ball HL. Misrecognition of need: women's experiences of and explanations for undergoing cesarean delivery. Social Science & Medicine, 2013; 85103-11. | Wrong phenomena |
| Wang E. Requests for cesarean deliveries: The politics of labor pain and pain relief in Shanghai, China. Social Science & Medicine, 2017; 1731-8. | Wrong phenomena |
| Wang E, Hesketh T. Large reductions in cesarean delivery rates in China: a qualitative study on delivery decision-making in the era of the two-child policy. BMC Pregnancy & Childbirth, 2017; 17 (1): 405. | Wrong phenomena |
| Wittmann-Price RA, Fliszar R, Bhattacharya A. Elective Cesarean births: are women making emancipated decisions? Applied Nursing Research, 2011; 24 (3): 147-52. | Wrong phenomena |
| York S, Briscoe L, Walkinshaw S, Lavender T. Why women choose to have a repeat caesarean section. British Journal of Midwifery, 2005; 13 (7): 440-45. | Wrong publication type |
| Zakerihamidi M, Roudsari RL, Khoei EM. Vaginal Delivery vs. Cesarean Section: A Focused Ethnographic Study of Women's Perceptions in The North of Iran. International Journal of Community Based Nursing & Midwifery, 2015; 3 (1): 39-50. | Wrong phenomena |
